# Supplementary material for: Pleistocene chronology and history of hominins and fauna at Denisova Cave
Source: Nat Commun. 2025 May 21;16:4738. doi: 10.1038/s41467-025-60140-6 (PMC12095498; doi:10.1038/s41467-025-60140-6)
Supplement: Supplementary file 1 — Supplementary Information [file 41467_2025_60140_MOESM1_ESM.pdf]

SUPPLEMENTARY METHODS, DISCUSSION and SUPPLEMENTARY FIGURES

SUPPLEMENTARY SECTION 1 | Stratigraphy, sedimentology, micromorphology, archaeology and palaeontology

1.1 Stratigraphy and sedimentology of Pleistocene deposits in South Chamber……………… 2
1.2 Micromorphology of sediment blocks from South and East Chambers…………………... 5
Supplementary Figure 1……………………………………………………………….. 5
Supplementary Figure 2……………………………………………………………….. 7
Supplementary Figure 3……………………………………………………………….. 8
Supplementary Figure 4……………………………………………………………….. 9
1.3 Palaeolithic artefacts in South Chamber……………………………………………….... 11
Supplementary Figure 5……………………………………………………………….. 13
Supplementary Figure 6……………………………………………………………….. 14
Supplementary Figure 7……………………………………………………………….. 15
Supplementary Figure 8……………………………………………………………….. 16
Supplementary Figure 9……………………………………………………………….. 17
Supplementary Figure 10……………………………………………………………… 18
1.4 Pleistocene record of large mammals in South Chamber………………………………… 19
Supplementary Figure 11……………………………………………………………… 21
1.5 Pleistocene record of small fauna in South Chamber…………………………………... 22

SUPPLEMENTARY SECTION 2 | Optical dating of sediments in South Chamber

2.1 Sampling strategy and sample descriptions……………………………………………….. 24
2.2 Sample preparation and analytical facilities……………………………………………. 25
2.3 Equivalent dose (D_e) estimation……………………………………………………….... 26
2.4 Environmental dose rate determination………………………………………………… 29
2.5 Age estimates and Bayesian models A and B…………………………………………... 34
Supplementary Figure 12……………………………………………………………… 37
Supplementary Figure 13……………………………………………………………… 41
Supplementary Figure 14……………………………………………………………… 42
Supplementary Figure 15……………………………………………………………… 43
Supplementary Figure 16……………………………………………………………… 44
Supplementary Figure 17……………………………………………………………… 45
Supplementary Figure 18……………………………………………………………… 46
Supplementary Figure 19……………………………………………………………… 47
Supplementary Figure 20……………………………………………………………… 48
Supplementary Figure 21……………………………………………………………… 49
Supplementary Figure 22……………………………………………………………… 50
Supplementary Figure 23……………………………………………………………… 52
Supplementary Figure 24……………………………………………………………… 53
Supplementary Figure 25……………………………………………………………… 54

SUPPLEMENTARY SECTION 3 | Sediment DNA of ancient hominins and fauna in South Chamber

3.1 Analysis of ancient mammalian mtDNA in sediments …………………………………… 55
3.2 Identification of ancient hominin mtDNA in sediments…………………………………... 56
Supplementary Figure 26……………………………………………………………… 57
Supplementary Figure 27……………………………………………………………… 59
Supplementary Figure 28……………………………………………………………… 61
Supplementary Figure 29……………………………………………………………… 63
Supplementary Figure 30……………………………………………………………… 64

SUPPLEMENTARY REFERENCES ……………………………………………………..65

SUPPLEMENTARY SECTION 1 | Stratigraphy, sedimentology, micromorphology, archaeology and palaeontology

1.1 Stratigraphy and sedimentology of Pleistocene deposits in South Chamber (Vladimir A. Uliyanov and Maxim B. Kozlikin)

Summary descriptions of the Pleistocene deposits in South Chamber are given in Supplementary Data 1. The layer numbering scheme follows that currently applied to the layers in South Chamber, with those used previously[1,2] shown in parentheses. As in Main and East Chambers[1], the deposits in South Chamber were excavated using a rectangular Cartesian coordinate system, with a total station used to measure and record the three-dimensional (X, Y, Z) locations of objects and features revealed during excavation. The location data were referenced to a datum point established outside of the excavated areas, which were divided into 1 × 1 m squares. The deposits exposed in the excavated sections were separated into lithostratigraphic units (layers) based on differences in granulometric composition and density, colour and tint. Distinctive inclusions, recent formations, textural and structural features, distinct or diffuse layer boundaries, unconformities due to gaps in sedimentation, and traces of biogenic and human activities were also documented. Visual descriptions of the stratigraphic sections were followed by further analyses, including micromorphology, to refine the lithostratigraphic units tentatively recognized in the field.

We reconstructed the sequence of sediment deposition and deformation in South Chamber based on assessments of the spatial relations of the stratigraphic units and the processes involved in the accumulation, erosion and post-depositional alterations of the cave sediments. We consider vertical subsidence associated with the slow, viscoplastic flow of clastic sediments as the main process responsible for deformation, with the uppermost Pleistocene layers also affected by phosphatization due to degradation of bat guano in the overlying Holocene deposits[3,4]. Animal burrowing is also responsible for some of the post-depositional features observed close to the boundary between layer 19 and the dMP deposits and in other Pleistocene layers, especially near the cave walls (as noted previously in East Chamber[1]). Anthropogenic disturbance is likely also a contributory factor to the deformation of sediments close to the Pleistocene–Holocene interface.

Earthquake-induced disturbances associated with ancient seismic events[5,6] may have influenced the rates of subsidence at different locations within the chamber, and the creation or enlargement of crevices next to the cave walls and the central ridge. Given the rheological properties of the sediments, however, we do not consider episodic earthquakes as the primary cause of deformation, but they may be responsible for some of the sharp boundaries observed in the sediments that infilled these crevices in stages. They may also have triggered sliding of coarser grained, more consolidated deposits (e.g., layer 11) over underlying finer-grained deposits (e.g., layer 12), resulting in the reorientation of flattened rock fragments along the sliding surface and the formation of soil flow textures, such as those preserved at the boundary between layers 12 and 11[6]. Micromorphological evidence of slippage and shear zones has previously been observed in sediments from Main Chamber (e.g., layers 22.1 and 20) and East Chamber (e.g., layers 11.4 and 11.3, and between layers 17 and 16)[7].

From analyses of the macrostratigraphic features of the South Chamber sequence, the angular unconformities between layers, and the sediment texture of each layer, we have reconstructed several stages in the deposition of the cave sediments and their subsequent deformation (Stages 1–6 below). The initial hypsometric positions of some layers can be established from remnants preserved in cracks and niches on the cave walls and on the surfaces of large blocks of spalled rock, and in the sediment infilling the spaces between the rocks that form a ridge along the central axis of the chamber. The total amplitude of subsidence of a layer since it was originally deposited can be estimated from the hypsometric position of its lower boundary. Based on observations of profiles exposed between 2018 and 2023 in the southwestern part of the excavation, the upper part of layer 19 has subsided by at least 3 m, the base of layer 16 by at least 1.3 m, and the base of layer 15 by at least 0.8 m, reflecting an increase in the extent of subsidence with age.

Progressive subsidence of the sediments filling the chamber led to the formation of fissures and expansion of cavities adjacent to the cave walls, into which loose sediment was then deposited. The resulting wedge-shaped deposits may consist of younger material deposited in association with the overlying layer, or coeval or older material redeposited from higher hypsometric levels in neighbouring or other parts of the chamber. This process explains the commonly observed decrease in hypsometric position of layer boundaries in the near-wall zones; the gradual constriction of layers at the contact with the cave walls; and the increase in thickness of layers closer to the central axis of the chamber. The depth of penetration of the wedge-shaped deposits is determined by the rate of growth of the side cavities relative to the rate of subsidence of main sediment body, with episodic earthquakes possibly contributing to differential subsidence rates.

As a consequence of subsidence and the central ridge of spalled rocks, the uppermost Pleistocene layers consist of a core of rocks and older sediments (e.g., layer 12) flanked by wedge-shaped infills of younger sediment located next to the cave walls (e.g., layer 11).

Reconstructed sequence of sediment accumulation and post-depositional deformation in South Chamber:

- Stage 1. Deposition of layer 19 in phreatic conditions, ending with a subhorizontal to horizontal upper surface at ~2 m depth below the modern cave datum (i.e., 1–3 m above its current position in Profiles A and D; Fig. 2c,d). Erosion of this surface likely occurred during the subsequent several tens of thousands of years, as indicated by the sharp boundary between layer 19 and the overlying deposits.
- Stage 2. Accumulation of layers 18, 17 and lower part of layer 16. Layers 18 and 17 were deposited before layer 19 was affected significantly by subsidence, based on their relatively consistent but limited thickness across the width of the chamber (e.g., Fig. 2d). By the time that layer 16 had begun to accumulate, however, subsidence of layers 19–17 had led to the formation of diapirs at the boundary of layer 19 and the overlying sediments. The convoluted geometry of layers 19–17 in Profile D (Fig. 2d) also reflects the chaotic, three-dimensional arrangement of spalled rocks in the centre of the chamber and the order in which they were deposited.
- Stage 3. Further subsidence during the period of accumulation of the upper part of layer 16 is indicated by its lens-shaped nature and variable thickness across the width of the chamber. The magnitude of subsidence, in addition to the deposition of sediments in the narrow, winding crevices formed next to the cave walls and along the central ridge of rocks, resulted in lateral compression of the deposits and further deformation of layers 19–17, leading to the overturning of folds and the pulling apart and rupture of these layers where they contacted the cave walls.
- Stage 4. A period of relatively intense erosion, as indicated by layer 15 and, in some areas, layer 14 truncating the boundaries of the underlying layers and the deformation structures developed during stages 1–3. Truncation of layer 15 by layer 14 (e.g., Fig. 2d) indicates that a period of erosion occurred between the deposition of these layers, which ended with the formation of a subhorizontal surface inclined slightly towards the cave walls.
- Stage 5. Deposition of layers 15–11 was accompanied by continued subsidence at variable rates across the chamber, but highest along both flanks of the central ridge to accommodate the accumulation of successive layers. The original hypsometric positions of the upper surfaces of these layers would have been slightly higher than their final elevations, reflecting the impact of subsidence and erosional processes. Owing to the presence of the ridge, sediment layers dip at angles of up to ~20° towards the cave walls (e.g., Fig. 2d), where cavities also formed and were subsequently infilled. Areas close to the walls may also have served as dens for cave-dwelling animals. In layer 11, for example, ~25% of the total number of identifiable bones of large mammals are of brown bear, wolf, red fox and cave hyaena (Supplementary Data 3) and the sediment DNA samples from this layer are likewise dominated by the mtDNA of ursids and canids (Supplementary Fig. 27a).

- Stage 6. Following formation of layer 11, sediment accumulation was replaced by a period of steady erosion through to the end of the Pleistocene, resulting in the eroded sediments from layers 12 and 11 being reworked and redeposited as layer 9. Cavities along the cave walls were also infilled by these mixed-age sediments. Layer 9 and large parts of layer 12 were subsequently affected by phosphate mineralization (pdd-9 and -12, respectively), in addition to disturbance by burrowing animals, cryoturbation (Supplementary Fig. 2) and possibly anthropogenic activities during the Holocene.

1.2 Micromorphology of sediment blocks from South and East Chambers (Paul Goldberg)

Sediment blocks were collected, prepared for analysis, examined with stereoscopic and petrographic microscopes at high magnification, and the micromorphological features described using procedures documented previously[7].

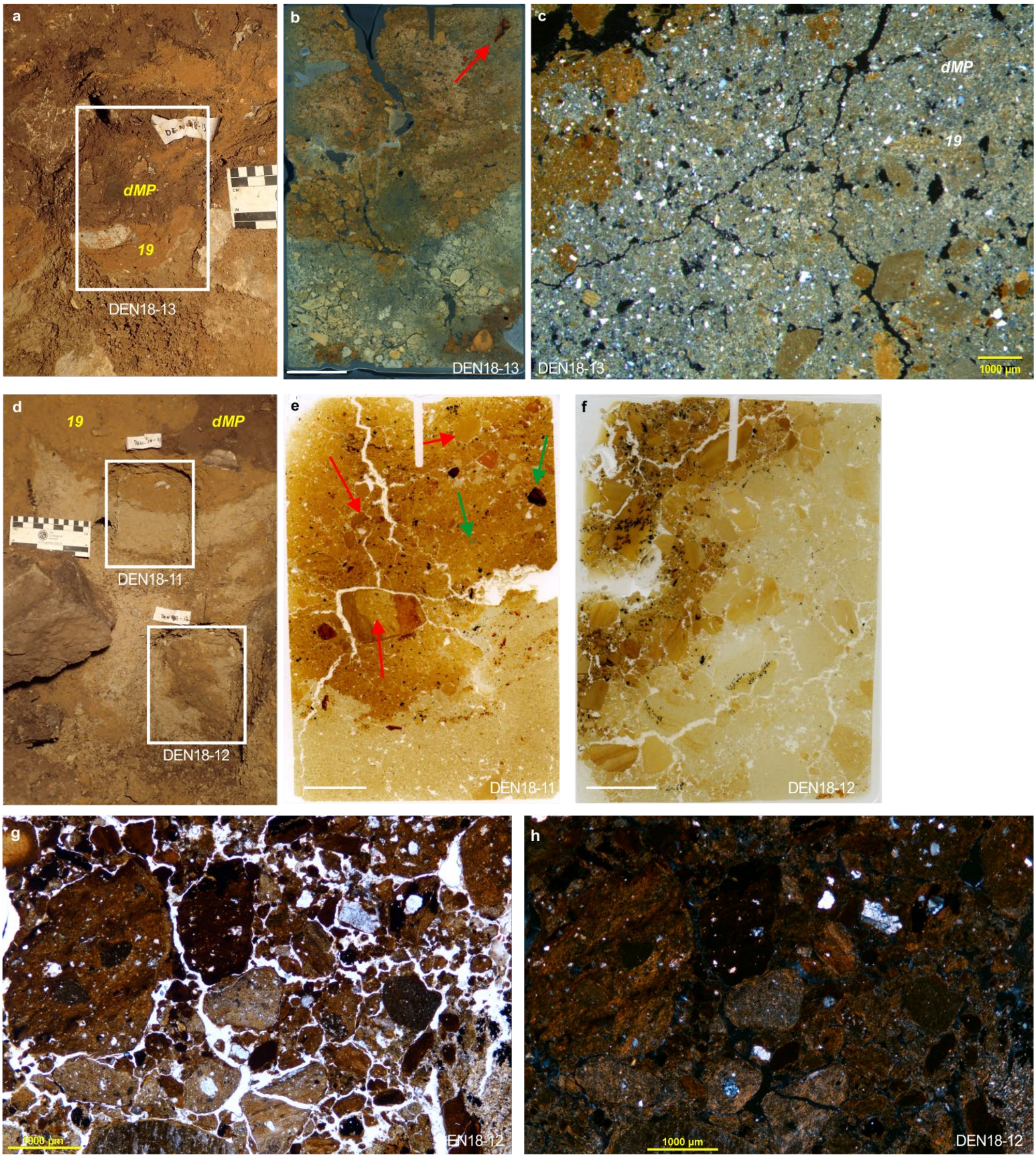

Supplementary Fig. 1 | Micromorphology of samples DEN18-11, -12 and -13 from South Chamber. a, Sample DEN18-13 (white rectangle) was collected from the lower southwest face of Profile A (Fig. 3a) and spans the upper part of layer 19 and the lower part of the dMP deposits. Stratigraphic layers are indicated in yellow text. **b**, Darkfield scan of thin section of DEN18-13 showing angular and rounded clasts of reworked phreatic sediment and a broken clast of iron pisolite (red arrow) in layer 19 (scale, 1 cm). The sediment is similar throughout the slide, with the colour

difference due to iron mobilization (gleying) in which the lighter area is deferrified. **c**, Photomicrograph (in XPL) of thin section of DEN18-13 at higher magnification showing the colour contrast between the lighter, deferrified sediments (centre) and the orange, iron-rich sediments (top left) in layer 19 (scale, 1 mm). Rip-up clasts of phreatic silty clay are visible at lower right. **d**, Samples DEN18-11 and -12 (white rectangles) were collected from the lower southeast face of Profile A (Fig. 3a). Stratigraphic layers are indicated in yellow text. **e**, Scan of thin section of DEN18-11 showing the contact between layer 19 and incorporated dMP deposits, which contain a mixture of clasts of phreatic sediment (red arrows) and rock and mineral grains (green arrows) in a reddish-brown silty clay matrix (scale, 1 cm). **f**, Scan of thin section of DEN18-12 showing the contact between layer 19 (right) and incorporated dMP deposits, which include manganese-stained clasts of reworked phreatic material (scale, 1 cm). **g**, Photomicrograph (in PPL) of thin section of DEN18-12 showing angular clasts of bedded, slightly silty clays (scale, 1 mm). The variation in colour from reddish-brown to pale yellow is due to iron depletion associated with oxidation/reduction. **h**, Same as **g** but in XPL, showing clearly the fine lamination within the clayey clasts, which are reworked from older phreatic sediments within the cave system (scale, 1 mm). Clasts of redeposited phreatic sediment are ubiquitous in layer 19 but also occur in most of the micromorphological samples examined from all three chambers at Denisova Cave.

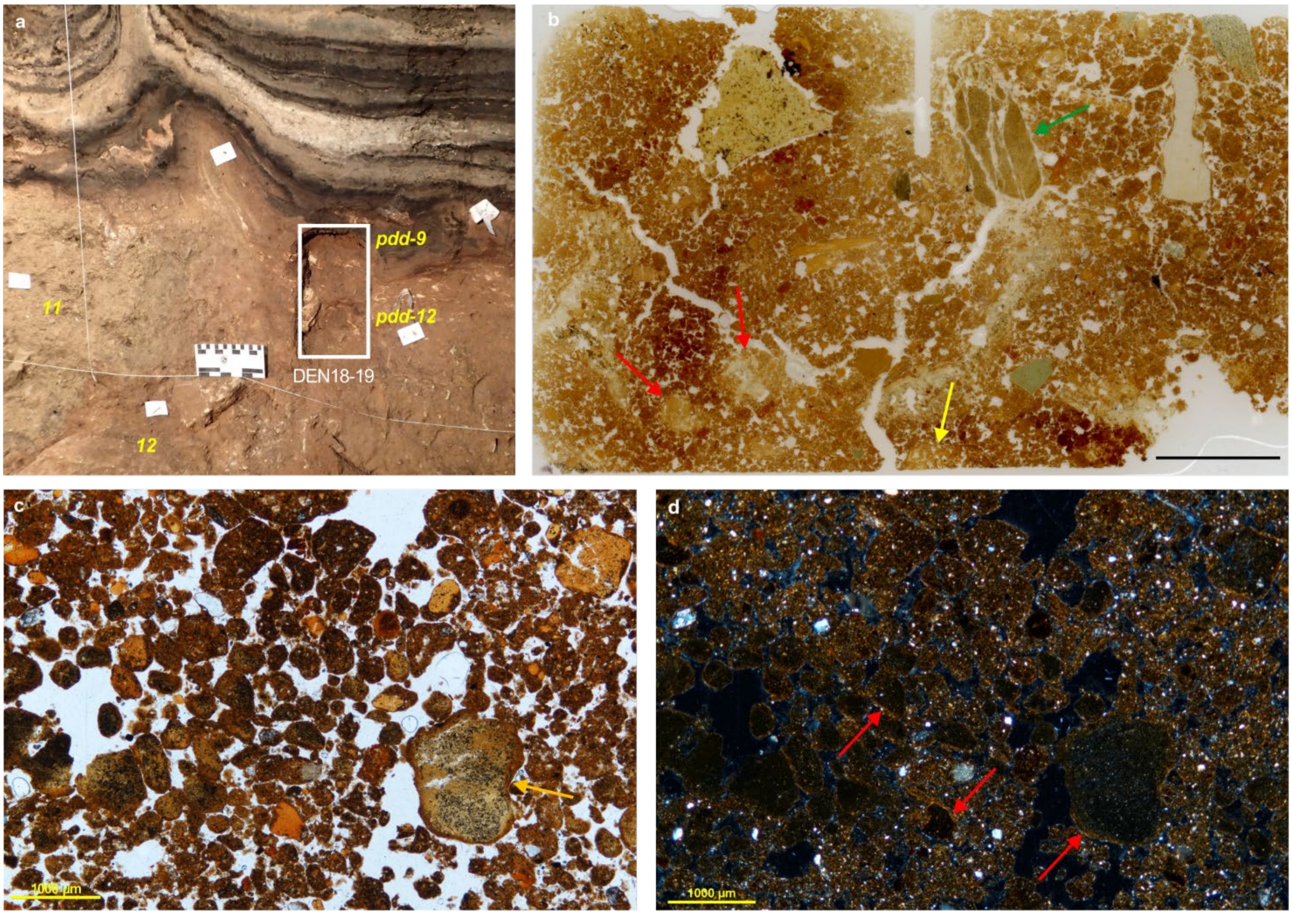

Supplementary Fig. 2 │ Micromorphology of sample DEN18-19 from South Chamber. a, This sediment block (white rectangle) was collected from the uppermost Pleistocene deposits in the southeast face of Profile C, directly beneath the organic-rich Holocene layers (Fig. 3e), and spans pdd-9 and -12. Stratigraphic layers are indicated in yellow text. **b**, Scan of thin section showing fragment of altered bone (yellow arrow), schist clast with apatite veins (green arrow) and compound phosphate nodules (red arrows) (scale, 1 cm). Note the rounded nature of the fine matrix material, due to cryoturbation. **c**, Photomicrograph (in PPL) of thin section from upper part of the sampled block showing cryoturbated, rounded grains of brown silty and coarser yellow brown coprolites, with a large example at lower right (orange arrow) (scale, 1 mm). **d**, Same as **c** but in XPL (scale, 1 mm). Note the yellow clay coatings around some of the grains (red arrows) produced by cryoturbation.

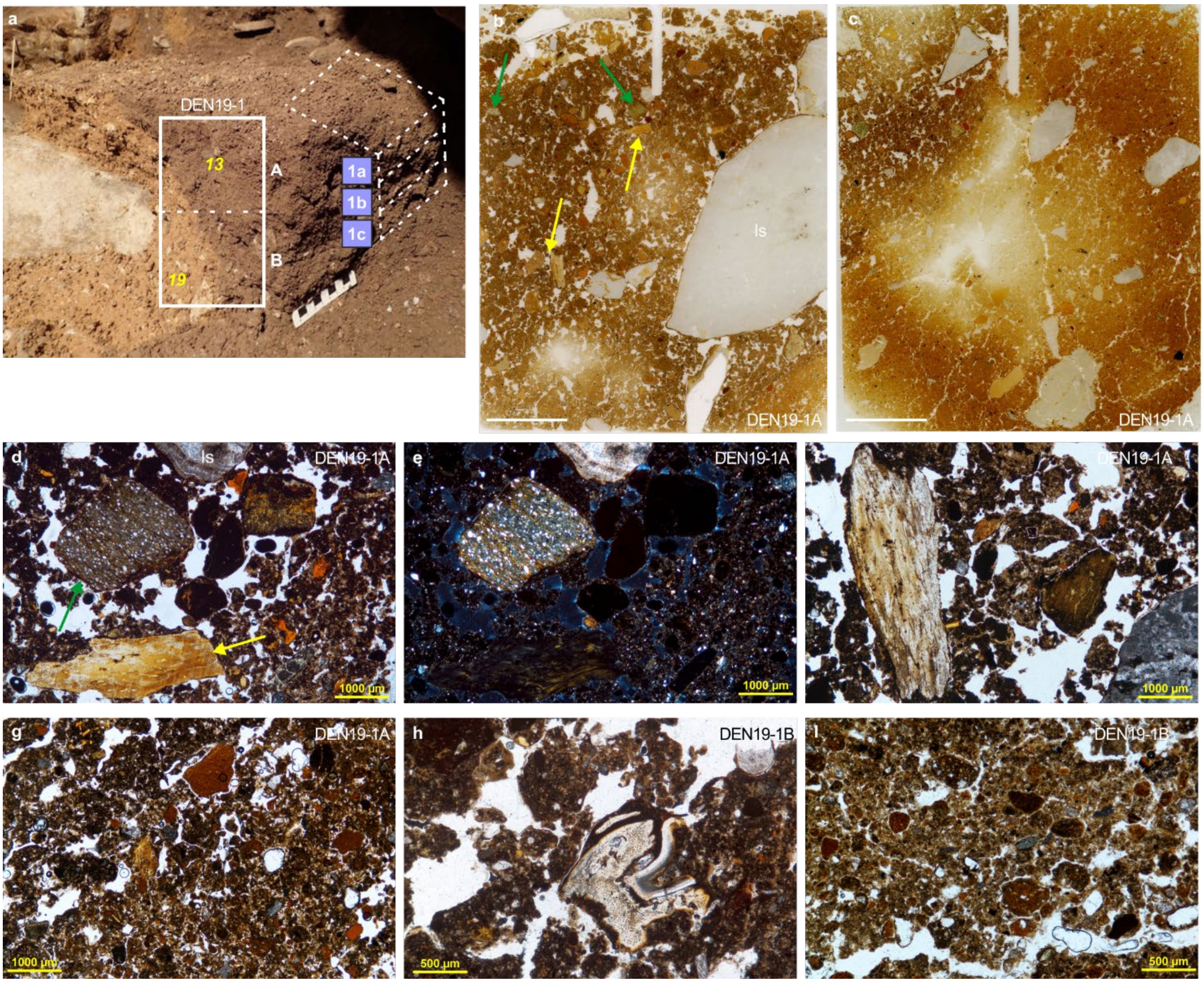

Supplementary Fig. 3 | Micromorphology) of sample DEN19-1 from South Chamber. a, This sediment block (A, upper part; B, lower part) was collected from the deposits adjacent to Denisova 22 and spans the upper part of layer 19 and the overlying sediments (layer 13). Stratigraphic layers are indicated in yellow text. The upper part of the block (A) consists mostly of layer 13 sediments, whereas the lower part (B) is bisected diagonally by the unconformable contact between layers 19 and 13. The coloured squares and dashed lines indicate the locations of optical dating samples DCS19-1a (top), -1b (middle) and -1c (bottom). **b**, Scan of thin section of part A showing a mixture of sand, silt and clay, with inclusions of limestone (ls), bone (yellow arrows), clasts of schist and siltstone (green arrows), and rounded aggregates of sediment from layer 19 (scale, 1 cm). **c**, Scan of thin section of part B showing a variety of clast types, including bone with differing extents of weathering, aggregates of bedded clay, fresh limestone and siltstone, and rare coprolites (scale, 1 cm). **d**, Photomicrograph (in PPL) of thin section from part A showing coarse fragments of iron-stained bone (yellow arrow), schist (green arrow) and limestone (ls) in a crumbly silty clayey matrix (scale, 1 mm). **e**, Same as **d** but in XPL (scale, 1 mm). **f**, **g**, Photomicrographs (in PPL) of thin sections from part A showing iron-stained bone in **f** and numerous aggregates and clasts, including bone, clay and silty clay, in **g** (scale, 1 mm). **h**, **i**, Photomicrographs (in PPL) of thin sections from part B showing weathered tooth fragment in aggregated silty clay (layer 13) in **h** and bioturbated and compacted pellets of varying compositions (upper part of layer 19) in **i** (scale, 500 µm).

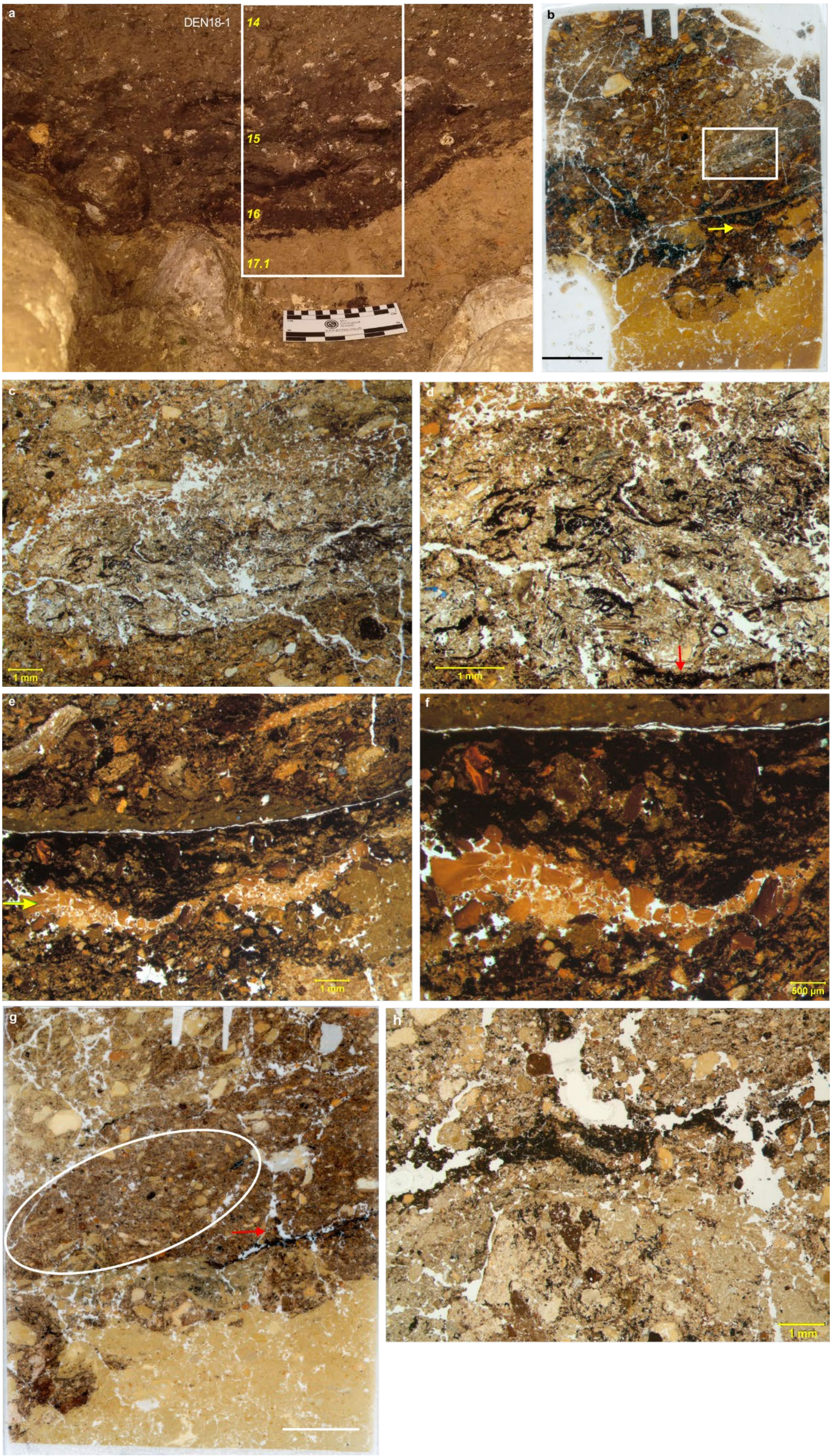
a
DEN18-1
14
15
16
17.1
b
c
1 mm
d
1 mm
e
1 mm
f
500 μm
g
h
1 mm

Supplementary Fig. 4 | Micromorphology of sample DEN18-1 from East Chamber. a, Location of sediment block (white rectangle) in the lower part of the middle section of the northwest profile showing the sharp, erosional contact between layer 17.1 (yellowish-brown silty clay) and layer 16 (dark, organic-rich sediment). The block extends beyond the top of the photograph. Stratigraphic layers are indicated in yellow text. **b**, Scan of thin section of lower part of block spanning the unconformable contact between layers 17.1 and 16 (scale, 1 cm). Pocket of fine charcoal and phytoliths (white rectangle) is shown at higher magnifications in **c** and **d**. Layer of burned, crushed bone (yellow arrow) is shown at higher magnifications in **e** and **f**. **c**, Photomicrograph (in PPL) of pocket of fine organic matter, phytoliths and yellow phosphatized ashes (scale, 1 mm). **d**, Detail of left-hand part of phosphatized ashy pocket in **c**. Note the fine charcoal with possible phytolith at the base (red arrow). **e**, Photomicrograph (in PPL) of layer 16 with burned and crushed bone indicated by yellow arrow (scale, 1 mm). **f**, Detail of crushed bone in **e** (scale, 500 µm). **g**, Scan of a different thin section of lower part of block showing the sharp contact between layers 17.1 and 16 (scale, 1 cm) and deposits composed of rounded aggregates of charcoal, fine organic matter and yellowish-brown silt clasts (white oval). Layer of dense organic matter (red arrow) shown at higher magnification in **h**. **h**, Photomicrograph (in PPL) of bedded organic matter (possibly mixed with manganese) at the contact between layers 17.1 and 16 (scale, 1 mm).

1.3 Palaeolithic artefacts in South Chamber (Michael V. Shunkov and Maxim B. Kozlikin)

Lithic and bone artefacts from South Chamber were collected during excavations in 2017–2022. The oldest lithic artefacts belong to the early Middle Palaeolithic and were found in layers 18 and 17[8,9]. The primary technology in this assemblage was aimed at obtaining mainly shortened and short flakes from radial cores (Supplementary Fig. 5: *3*) on pebbles or large, massive flakes. Parallel flaking characterizes individual specimens of single platform cores (Supplementary Fig. 5: *13*) on large pebbles. Single blades are also present in the assemblage (Supplementary Fig. 5: *1*). Tools are mainly represented by ventrally thinned (Supplementary Fig. 5: *4, 10–12*) and basally truncated (Supplementary Fig. 5: *7*) flakes. The set of side-scrapers includes samples with straight (Supplementary Fig. 5: *9*), diagonal (Supplementary Fig. 5: *6*) and transverse (Supplementary Fig. 5: *5*) shapes, including Quina-like retouch (Supplementary Fig. 5: *8*). A variety of denticulate, notched (Supplementary Fig. 5: *2*) and spur-like tools, modified by heavy retouch, are common.

The further development of Middle Palaeolithic assemblages is reflected in the lithic industries from layers 16 to 12[9–13]. Most of the finds are associated with layers 13 and 12, with the fewest artefacts found in layer 14 (Supplementary Data 2). Overall, in terms of the set of cores, these industries are characterized by a combination of developed parallel and Levallois flaking, as well as active removal of blanks from radial cores (Supplementary Fig. 6: *3*). Parallel cores are represented by unidirectional (Supplementary Fig. 7, *13*) or bidirectional specimens, formed on large pebbles and less often on massive flakes. Levallois cores are intended for obtaining flakes (Supplementary Fig. 6: *1, 2*) and points (Supplementary Fig. 7: *12*). Radial cores are represented by residual, mostly unifacial, items.

The blanks are dominated by flakes, which are characterized by shortened or short proportions, and smooth or natural residual striking platforms. The dorsal scar patterns on the flakes are predominantly longitudinal unidirectional or orthogonal. The proportion of blades among the blanks is 4.5–4.9% in the collections from layers 15, 13, and 12, and 8.2% in layer 14. Blades (Supplementary Fig. 7: *2, 11*) most often have faceted residual striking platforms and are longitudinal unidirectional or bidirectional, while orthogonal dorsal scar patterns are less common.

The tools in the collections from layers 16 and 15 are mainly based on ventrally thinned (Supplementary Fig. 7: *9*) and basally truncated (Supplementary Fig. 7: *3*) flakes. To a lesser extent, side-scrapers and denticulate-notched forms are represented and there are Levallois points. Higher in the section, side-scrapers become the predominant component among the tools, the largest number of which was recorded in the collection from layer 12. Items of this category are distinguished by typological diversity: single or double straight, diagonal (Supplementary Fig. 7: *10*), convergent symmetrical and *déjeté*-like types (Supplementary Fig. 7: *7*), transverse (Supplementary Fig. 7: *5*) and semi-circular with different working edge shapes. Large whole or fragmented flakes were used as blanks. Side-scrapers with basal thinning or with truncation, as well as backed forms, are also present.

Individual specimens in the collections from layers 14 to 12 include Levallois (Supplementary Fig. 7: *1*) and Mousterian (Supplementary Fig. 7: *8*) points. A large, subtriangular biface found in layer 14 (Supplementary Fig. 6: *4*) has no analogue in Denisova Cave. Denticulate, notched and spur-like tools of various types are common. Artefacts typical of the Upper Palaeolithic were found mainly in the assemblage from layer 12, where they are represented by an end-scraper (Supplementary Fig. 7: *6*), burins (Supplementary Fig. 7: *4*) and truncated-faceted flakes or blades. Layer 12 also contains the largest collection of bone retouchers in the cave, including tools with varying degrees of shaping and utilization.

The Upper Palaeolithic finds in South Chamber are associated with layer 11[9–11,13,14]. Large, elongated blanks (Supplementary Fig. 8: *8, 10, 12*) in this assemblage were obtained from parallel unidirectional or bidirectional (Supplementary Fig. 8: *11*) cores on pebbles. Radial cores are rare. Small blades were flaked from subprismatic cone-shaped cores (Supplementary Fig. 8: *1*). The collection of flakes is dominated by specimens with smooth, residual striking platforms and longitudinal dorsal scar patterns. The proportion of blades in the Upper Palaeolithic assemblage is

14%, which is significantly higher than in the Middle Palaeolithic collections. Among the blades, very elongated specimens (l:m = 5:1) (Supplementary Fig. 8: *9*; Supplementary Fig. 9: *31*) were most likely made using a soft hammer. Bladelets and microblades are also present in the collection.

A fairly large proportion of the Upper Palaeolithic collection of tools consists of side-scrapers of single or double straight (Supplementary Fig. 8: *4*, *7*; Supplementary Fig. 9: *23*), diagonal, transverse and convergent (Supplementary Fig. 8: *3, 5*) types. These are associated mainly with the lower part of layer 11, as are several atypical Levallois points (Supplementary Fig. 8: *6*). The expressive Upper Palaeolithic component of the industry is represented by end-scrapers (Supplementary Fig. 9: *19–21, 28, 29*) on flakes or blades, burins on blades (Supplementary Fig. 8: *2*; Supplementary Fig. 9: *24, 27*), retouched blades (Supplementary Fig. 9: *30*) and splintered pieces (Supplementary Fig. 9: *26*). Biconvex bifacial points are represented by several specimens (Supplementary Fig. 9: *18, 22*) and denticulate, notched and spur-like tools are also present in the collection. The upper part of layer 11 yielded a high concentration of bladelets (Supplementary Fig. 9: *12–17*) and microblades (Supplementary Fig. 9: *1, 2, 6, 8, 10, 25*), including backed specimens (Supplementary Fig. 9: *3–5, 7, 9*). Thus, the lithic industry in layer 11 reflects the dynamics of the Upper Palaeolithic complex during the early and middle stages of this archaeological phase, consistent with the diachronous history of formation of this layer indicated by the geochronological data.

In addition to lithic artefacts, bone tools[15–18] were also found in layer 11. Unshaped tools are represented by retouchers, pressure flaking tools and smoothers. Formal implements include a needle with a drilled eye, a spatula and awls (Supplementary Fig. 10: *22, 23*).

More than 60 non-utilitarian items made from animal teeth and bones, mammoth tusk, soft stone (serpentine, chrysotile, talc, marble), ostrich eggshell and freshwater mollusc shell have been collected from layer 11[15,19–24]. These ornaments include beads (Supplementary Fig. 10: *1–4, 10*), pendants (Supplementary Fig. 10: *5–7, 9, 13–16*), tubular beads (Supplementary Fig. 10: *8, 11*), rings (Supplementary Fig. 10: *12, 19*), fragments of a diadem (Supplementary Fig. 10: *20*) and bracelet (Supplementary Fig. 10: *17*), and a perforated plate (Supplementary Fig. 10: *21*). Figurative art is represented by a zoomorphic sculpture (Supplementary Fig. 10: *18*), most likely of a predator in the cat family, but lacking the head and forelimbs[23]. The figurine is ornamented on all sides with uniform rows of four short notches, within which the remains of a red mineral pigment are preserved.

Many of the bone tools and ornaments were recovered from the lower part of layer 11, which allows us to attribute them, by analogy with the lithic industry, to the Early Upper Palaeolithic.

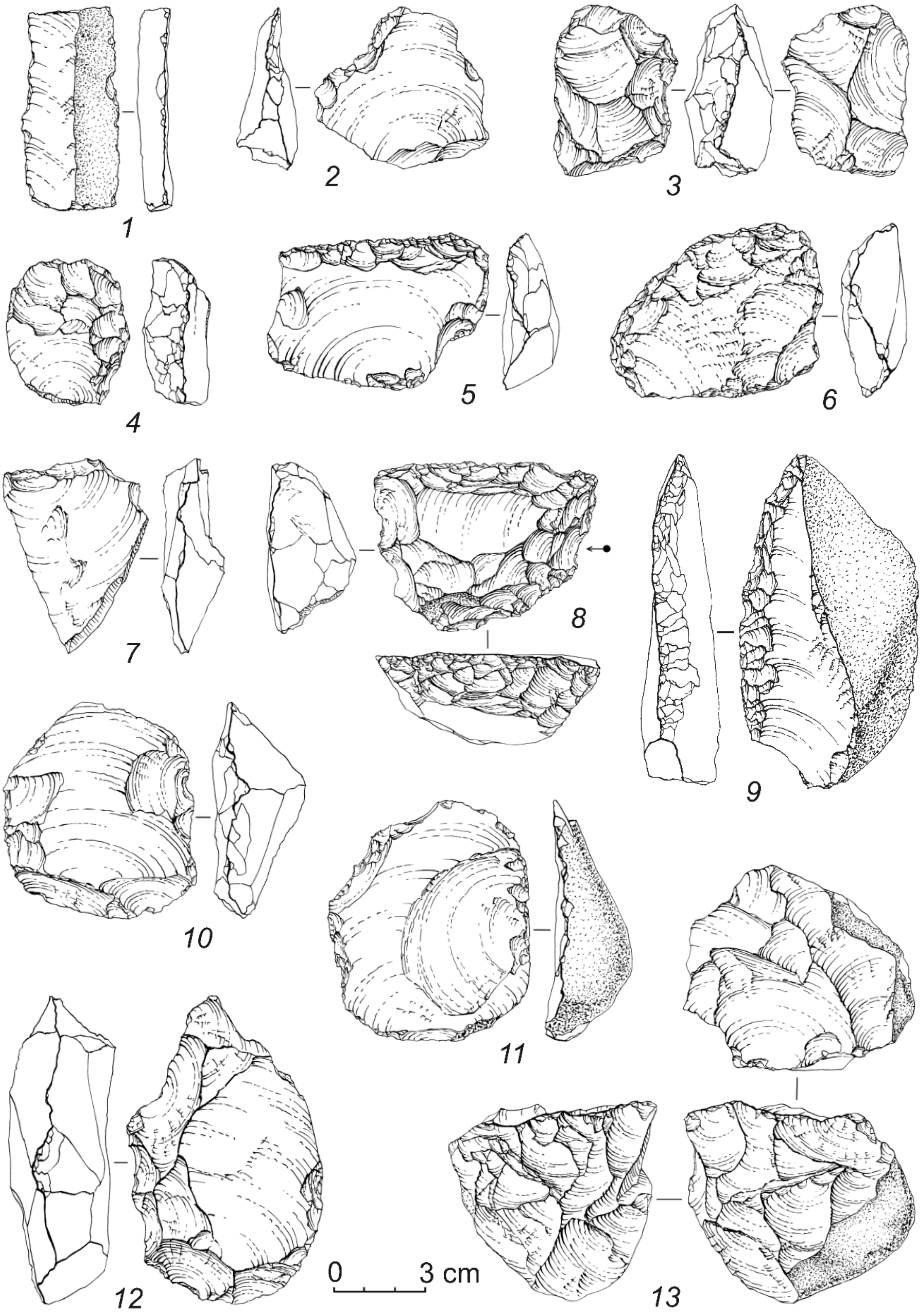

Supplementary Fig. 5 | Lithic artefacts (early Middle Palaeolithic) from layers 18 and 17 in South Chamber. *1* – blade; *2* – notched tool; *3, 13* – cores; *4, 10–12* – ventrally thinned flakes; *5, 6, 8, 9* – side-scrapers; *7* – basally truncated flakes. Reproduced with permission from the Institute of Archaeology and Ethnography of the Siberian Branch of the Russian Academy of Sciences.

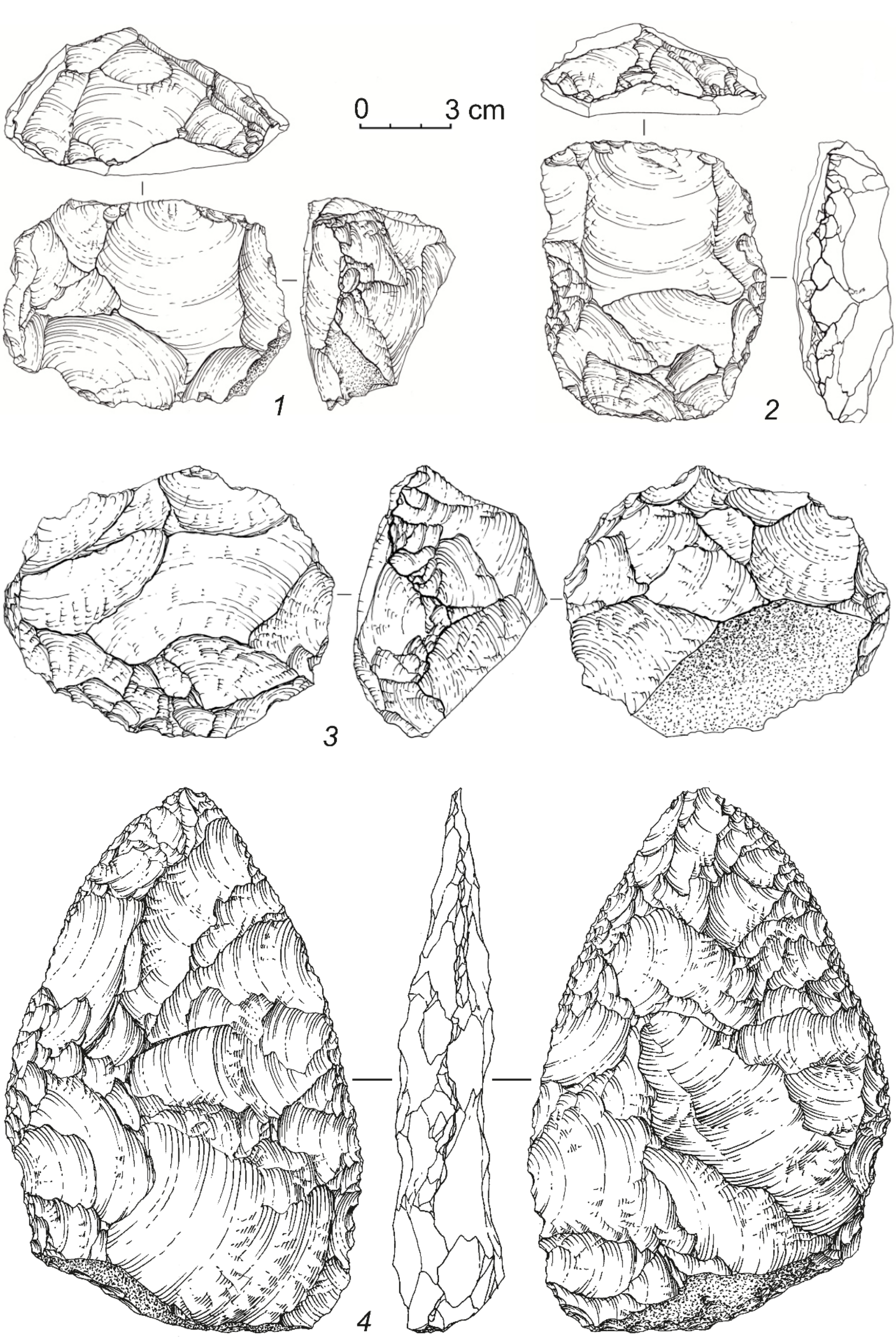

Supplementary Fig. 6 | Lithic artefacts (middle Middle Palaeolithic) from layers 16–12 in South Chamber. *1–3* – cores; *4* – biface. Reproduced with permission from the Institute of Archaeology and Ethnography of the Siberian Branch of the Russian Academy of Sciences.

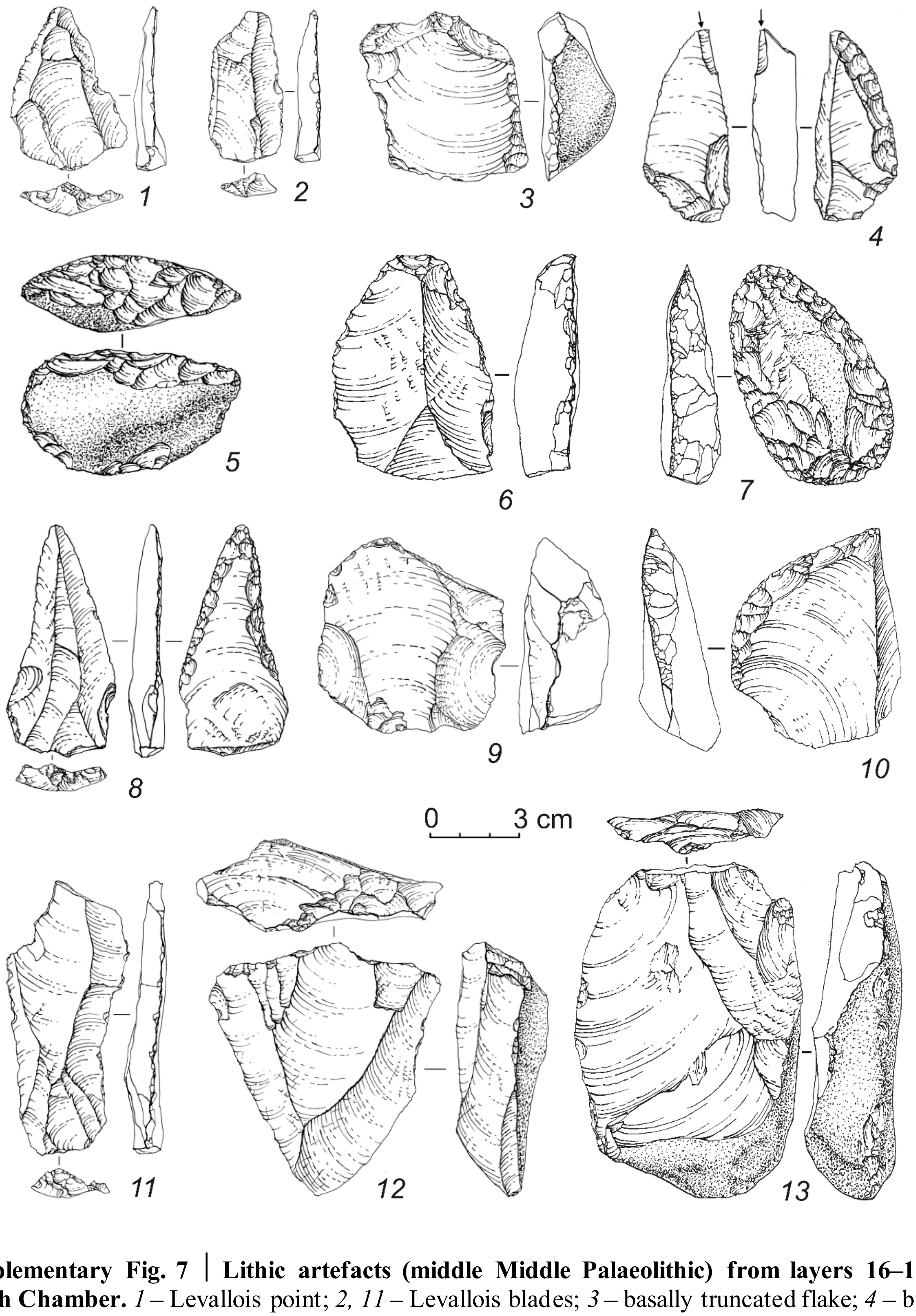

Supplementary Fig. 7 | Lithic artefacts (middle Middle Palaeolithic) from layers 16–12 in South Chamber. *1* – Levallois point; *2, 11* – Levallois blades; *3* – basally truncated flake; *4* – burin; *5, 7, 10* – side-scrapers; *6* – end-scraper; *8* – Mousterian point; *9* – ventrally thinned flake; *12, 13* – cores. Reproduced with permission from the Institute of Archaeology and Ethnography of the Siberian Branch of the Russian Academy of Sciences.

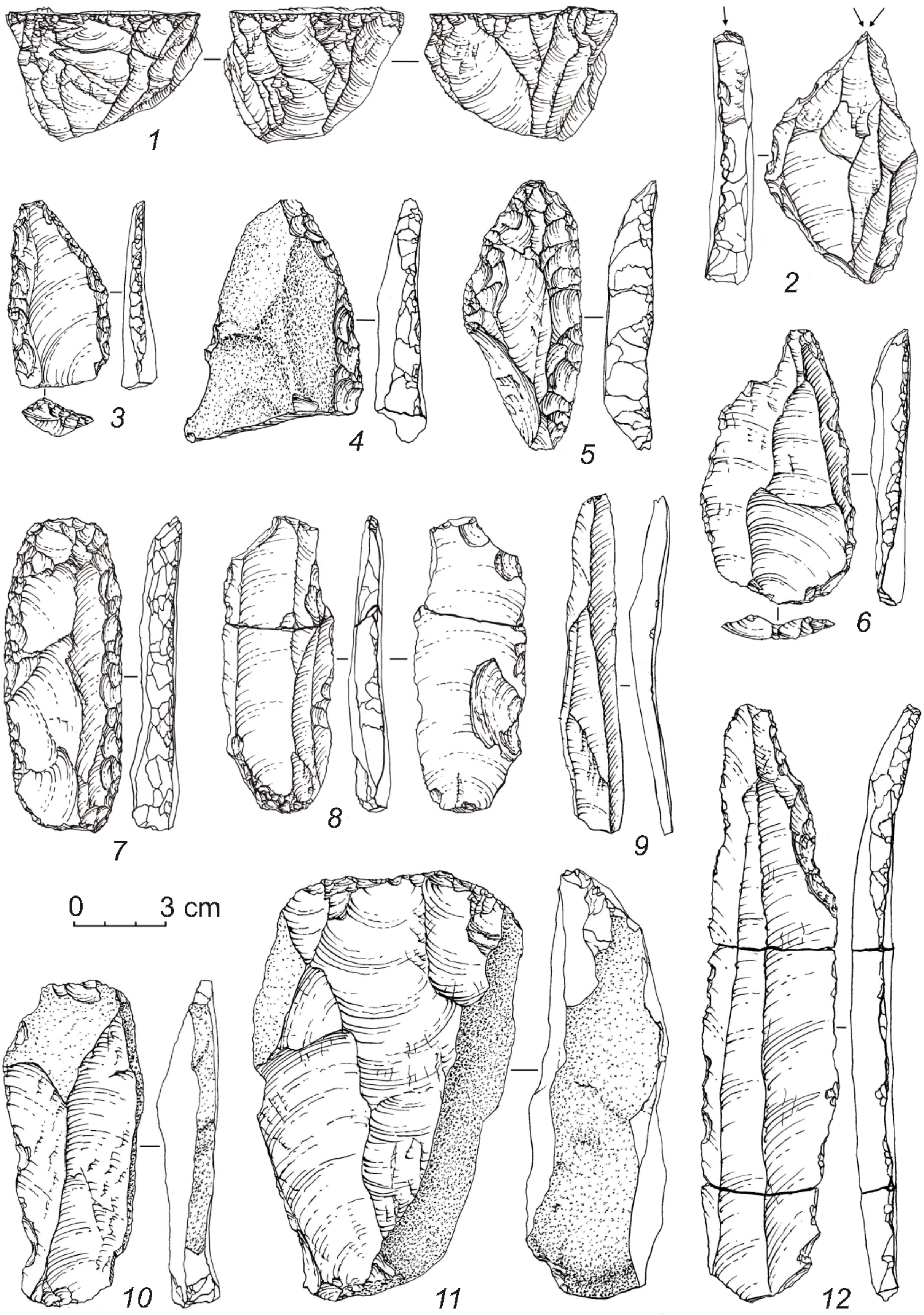

Supplementary Fig. 8 | Lithic artefacts (Upper Palaeolithic) from layer 11 in South Chamber. *1, 11* – cores; *2* – burin; *3–5, 7* – side-scrapers; *6* – Levallois point; *8–10, 12* – blades. Reproduced with permission from the Institute of Archaeology and Ethnography of the Siberian Branch of the Russian Academy of Sciences.

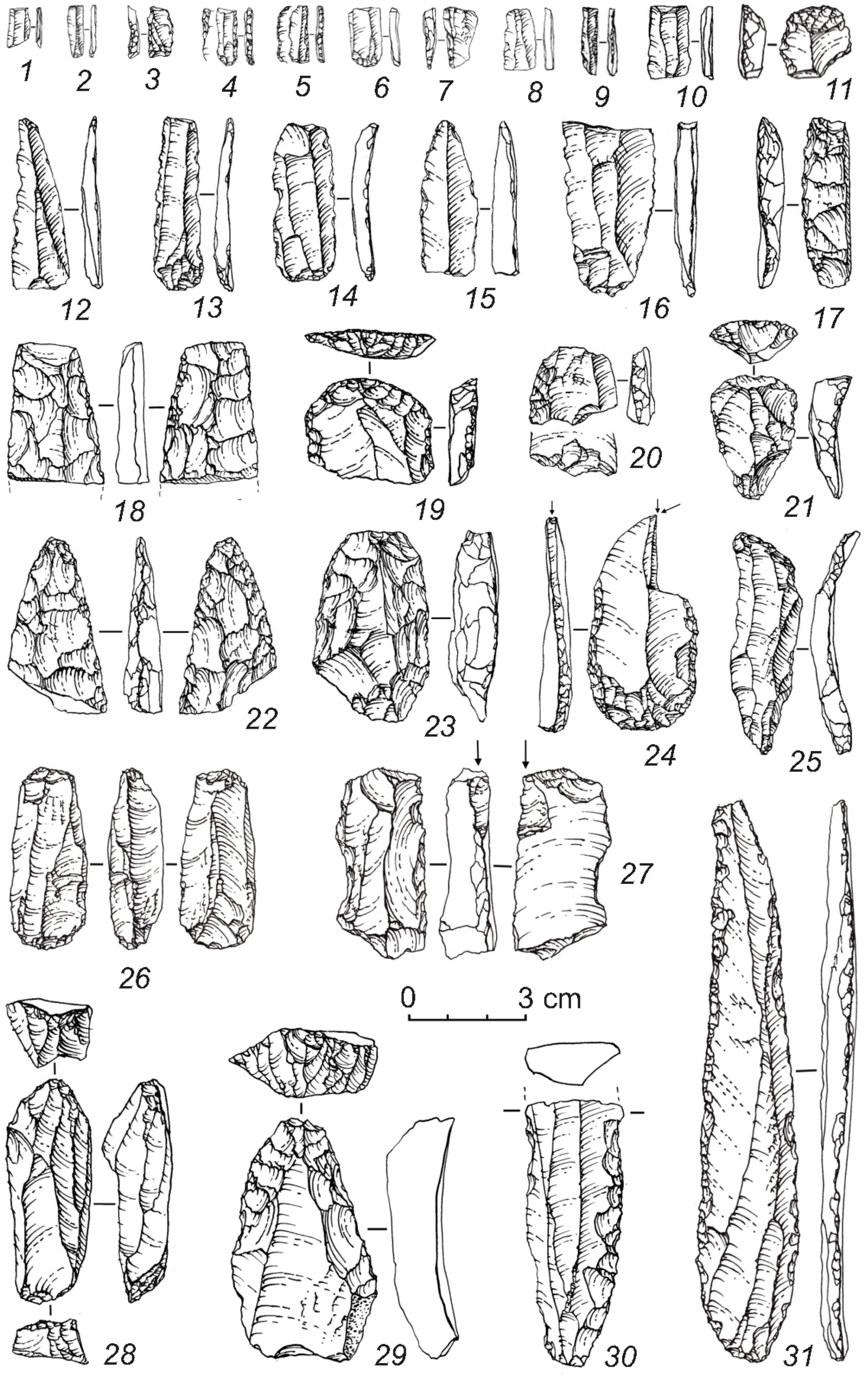

Supplementary Fig. 9 | Lithic artefacts (Upper Palaeolithic) from layer 11 in South Chamber. *1, 2, 6, 8, 10* – microblades; *3–5, 7, 9* – backed microblades; *11, 19–21, 28, 29* – end–scrapers; *12–17, 25* – blades; *18, 22* – fragments of bifacial points; *23* – side-scraper; *24, 27* – burins; *26* – splintered piece; *30, 31* – retouched blades. Reproduced with permission from the Institute of Archaeology and Ethnography of the Siberian Branch of the Russian Academy of Sciences.

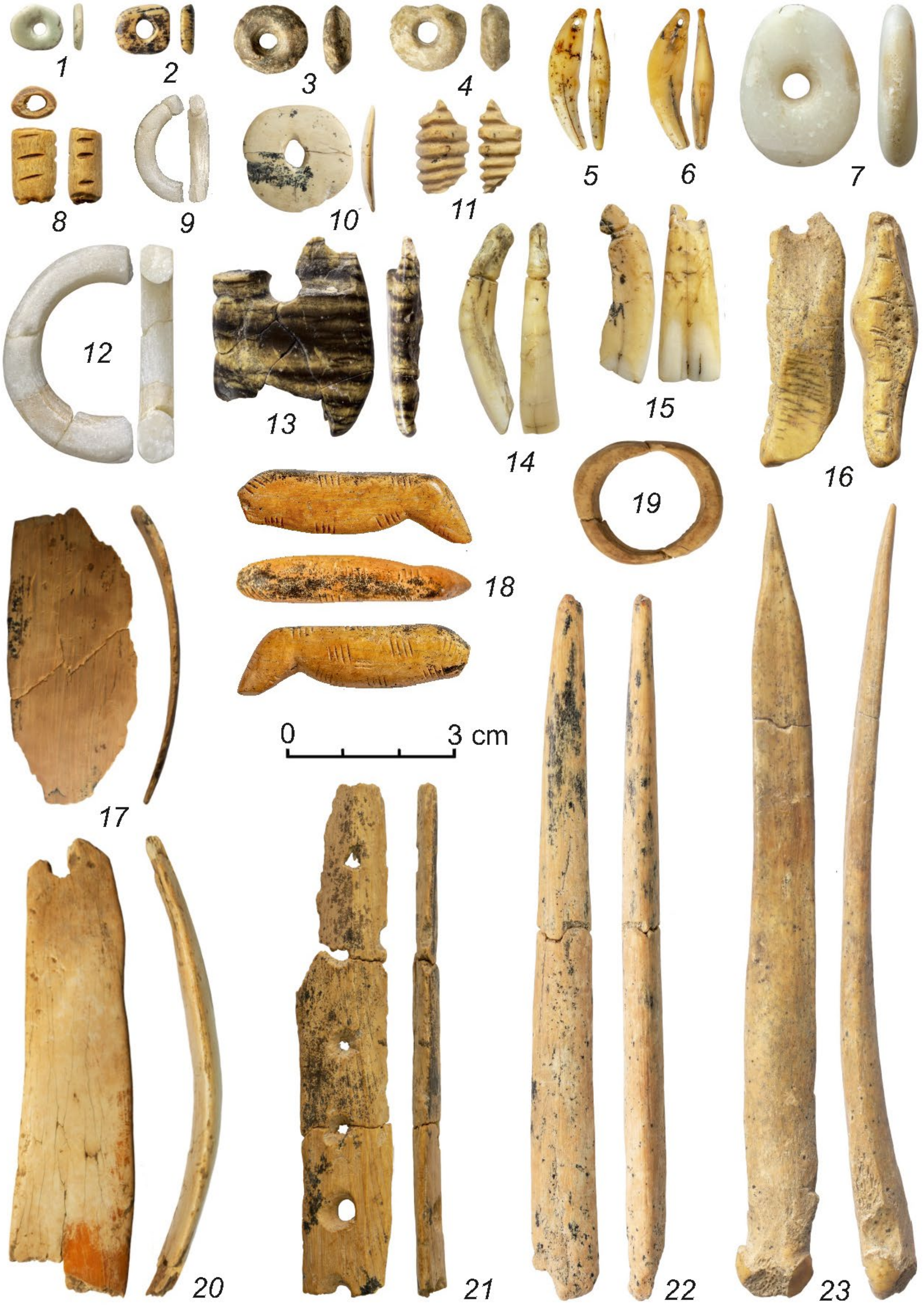

Supplementary Fig. 10 | Ornaments and bone tools (Upper Palaeolithic) from layer 11 in South Chamber (photos by A. Yu. Fedorchenko). *1–4, 10* – beads; *5–7, 9, 13–16* – pendants; *8, 11* – tubular beads; *12, 19* – rings; *17* – fragment of a bracelet; *18* – zoomorphic sculpture; *20* – fragment of a diadem; *21* – perforated plate; *22, 23* – awls. These artefacts were made from marble (*1, 7, 9, 12*), mammoth tusk (*2, 10, 17–21*), talc (*3, 4*), animal teeth (*5, 6, 14, 15*), bone (*8, 11, 16, 22, 23*) and chrysotile (*13*). Reproduced with permission from the Institute of Archaeology and Ethnography of the Siberian Branch of the Russian Academy of Sciences.

1.4 Pleistocene record of large mammals in South Chamber (Sergei K. Vasiliev, Michael V. Shunkov and Maxim B. Kozlikin)

Excavations of Pleistocene deposits in South Chamber in 2019–2021 yielded more than 130,000 bone remains, of which 7,925 specimens could be taxonomically identified, representing at least 43 species of large mammals (Supplementary Data 3)[25–27].

The proportion of identifiable remains varies from 12.2% (layer 19) to 3.2% (layer 17), and averages 6% across all Pleistocene layers. Bones and teeth are mostly fragmentary, ranging in size from 1–2 cm (66% of the Pleistocene assemblage, averaged across all layers) to 2–5 cm (32% of the assemblage, on average). Larger fragments are much less abundant, comprising just 2.0% (5–10 cm) and 0.2% (longer than 10 cm) of the identifiable remains. The number of small fragments increases slightly down the section, especially in layers 17 and 18. In general, fragments of teeth and distal limb bones predominate among the identifiable remains. The larger the animal, the higher the proportion of remains of its teeth or tooth fragments.

The bones are structurally dense and strong, they lack signs of cracking, peeling or weathering and are very well preserved throughout the section (Supplementary Fig. 11). Bone surfaces are smooth and shiny, with black manganese dendrites commonly present. The degree of fossilization is weak and hygroscopicity is practically absent, even for bones from the oldest layers.

Traces of carnivore activity by large predators, primarily cave hyaenas, are clearly visible on bone and tooth remains (Supplementary Fig. 11: *11*), especially those from the upper part of the section (layers 11 and 12). Some fragments of bones and teeth bear traces of digestion (Supplementary Fig. 11: *1*), formed in the gastrointestinal tract of these predators. Most of the large bone fragments show evidence of severe gnawing. Evidence of human impact on bone remains is greatest in the collections from layers 17 and 18, which are associated with early Middle Paleolithic occupation. These collections contain bone fragments and, in rare cases, whole burnt bones (Supplementary Fig. 11: *8*), specimens with numerous or single cutmarks (Supplementary Fig. 11: *4–7*) and traces of meat cutting, split and chipped bones (Supplementary Fig. 11: *10, 12*), and bone flakes (Supplementary Fig. 11: *2, 3*) with a platform and a bulb of percussion.

The large mammal remains make it possible to ascertain, with a degree of reliability dependent on sample size, the dynamics of change in the natural environment during the accumulation of layers 19–11. We identified four biotope groups of large mammals (steppe, forest-steppe, forest and rocky), as well as five intrazonal species (fox, grey wolf, wolverine, cave lion and mammoth). Steppe biotope species include corsac, steppe polecat, small cave bear, cave hyaena, woolly rhinoceros, Ovodov's horse, giant deer (Supplementary Fig. 11: *13*), Baikal yak, bison, Kyakhta antelope, gazelle, saiga and argali. The forest-steppe biotope group includes dhole, large caballoid horse, elk and red deer. Forest biotope species include sable, brown bear (Supplementary Fig. 11: *9*), lynx, roe deer and beaver, while the rocky biotope group includes Siberian ibex, snow leopard and mountain weasel.

The large mammal remains in layers 19 and 18 are dominated by the forest (34–38%) and steppe (31%) biotope groups (Fig. 10d; Supplementary Data 4). The proportion of steppe biotope species increases to 50% in layer 17, while the proportion of forest biotope species falls to 13%. The forest-steppe and rocky biotope groups represent 18–21% and 12–17%, respectively, of the large mammal collections in layers 19–17. In layers 16 and 15, the proportion of steppe biotope species increases further, to 64–65%, with the forest-steppe and forest biotope groups accounting for 10–14%. In layer 14, the steppe biotope group represents a smaller, but still dominant, component of the large mammal collection (46%). The proportion of rocky biotope species increases to 34%, while that of forest-steppe and steppe biotope groups remains largely unchanged (10%). The steppe biotope group continues to dominate the large mammal collections in layers 13–11 (49–62%), followed by rocky biotope species (27–36%) and a small proportion of forest-steppe and forest biotope species (4–14%).

The remains of Siberian ibex are most abundant in layers 14–11. The rocky biotope group is most prominent during the accumulation of these layers, while the forest-steppe and forest biotope groups comprise the smallest proportions (Fig. 10d; Supplementary Data 4). The sole representative

of the conditionally tundra biotope group is the reindeer, the remains of which occur only in layers 16, 12 and 11 (Supplementary Data 3).

The presence of these biotope groups during the formation of the Pleistocene layers in South Chamber is consistent with a landscape mosaic typical of mountainous regions, but with a lower proportion of open meadow-steppe and rocky landscapes before ~170 ka (layers 19–17) than after ~80 ka (layers 13–11).

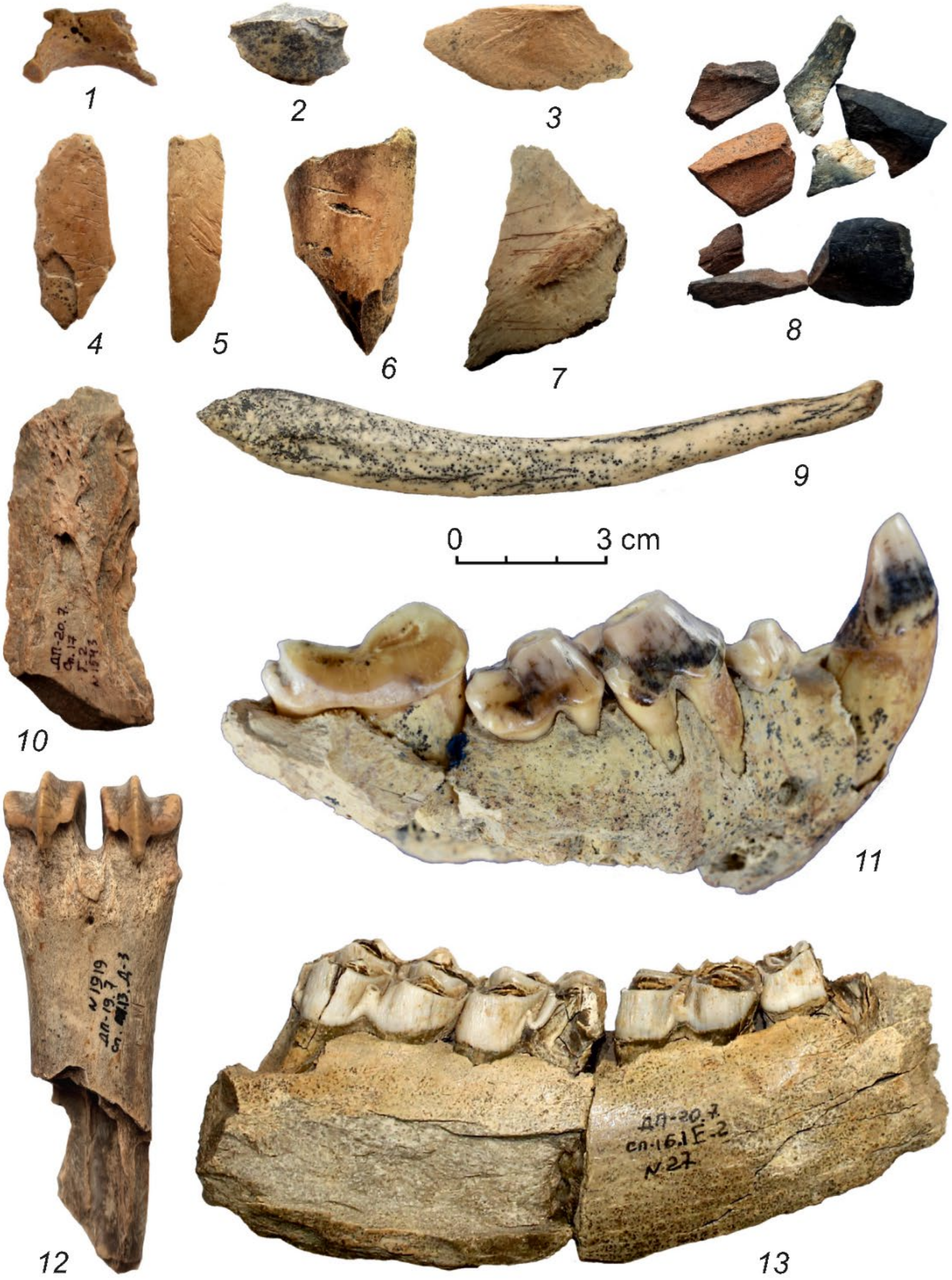

Supplementary Fig. 11 | Large mammal remains from the Pleistocene deposits in South Chamber. *1* – diaphysis of cave lion (*Panthera leo spelaea*), second phalanx with traces of digestion[27]; *2, 3* – bone flakes; *4–7* – bone fragments with cutmarks; *8* – burnt bones; *9* – baculum of brown bear (*Ursus arctos*)[27]; *10, 12* – bones with traces of splitting; *11* – mandibula fragment of cave hyaena (*Crocuta crocuta spelaea*); *13* – mandibula fragment of giant deer (*Megaloceros giganteus*)[26]. Reproduced with permission from the Institute of Archaeology and Ethnography of the Siberian Branch of the Russian Academy of Sciences.

1.5 Pleistocene record of small fauna in South Chamber (Alexander K. Agadjanian, Michael V. Shunkov and Maxim B. Kozlikin)

Bone remains of small vertebrates were collected from Pleistocene deposits in the central part of South Chamber[28,29]. A total of 30,327 specimens were examined from layers 19 to 11 (Supplementary Data 5) and the community structure was determined from 21,489 bones of small vertebrates. Species and genera were identified for 19,638 specimens. To further assess the dynamics of the ecological composition of small terrestrial fauna at high resolution, we excluded the bone remains of bats, birds and fish (which represent 25–60% of the total number of specimens in the Pleistocene layers). As a result, a total of 16,669 specimens were used to determine the composition of fossil communities of small vertebrates (Supplementary Data 6) and the corresponding local climatic conditions relative to the current, moderately continental, climate outside Denisova Cave (mean temperatures of −16 °C and 18 °C in January and July, respectively). Overall, the dynamics of the taxonomic composition of small vertebrates reflect ecological changes associated with periods of relatively cold or warm climate during the Middle and Late Pleistocene.

The community of small mammals from layer 19 includes shrews of the genus *Sorex*, the mole *Asioscalops*, the red-backed vole *Clethrionomys*, the chipmunk *Cricetus cricetus*, and the flying squirrel *Pteromys*, which are indicators of relatively favourable environmental conditions. The mountain vole *Alticola* is well represented in the fossil assemblage from this layer, and populations of the narrow-headed vole *Stenocranius gregalis*, the ground squirrel *Spermophilus* and the marmot *Marmota* are also recorded. The last two species hibernate during winter, lowering their body temperature and metabolic rate. Reducing their activity during periods of low temperature and soil freezing allows them to survive until spring, and has enabled ground squirrels to spread along river valleys north to the Arctic Circle and for marmots to inhabit high mountain plateaus. They cannot live in tall grass meadows with dense turf, preferring open landscapes with sparse grass in cold climates. Layer 19 also yielded bones of the lemming *Lemmus*, a boreal component of the fauna, and Siberian zokor *Myospalax myospalax*, the latter in relatively low abundance. Remains of the mole vole *Ellobius*, a rodent that lives underground and feeds on underground parts of plants, were not recovered from this layer. The mole vole and zokor are active all year round and cannot exist in conditions of long-term and deep freezing of the soil, so their low abundance or absence in the fossil assemblage reflects intense cryogenic processes in the soil. The composition of the main ecological groups of small mammals is, therefore, consistent with the formation of layer 19 towards during the marine isotope stage (MIS) 8 glacial period (Fig. 10d).

The main groups of small vertebrates in the fossil assemblages from layers 18 and 17 have similar characteristics to those in layer 19, including *Sorex* shrews, moles, ground squirrels and mole voles. The proportions of steppe lemming *Lagurus* and voles (the flat-headed *Alticola strelzowi*, the narrow-headed, root vole *Microtus oeconomus*, and the North Siberian vole *Microtus hyperboreus*) are also similar in all three layers. The proportions of forest voles *Clethrionomys*, Siberian zokor, Baraba hamster *Cricetulus barabensis* and Eversmann's hamster *Allocricetulus eversmanni* increase slightly from layer 18 to layer 17. Remains of the shrews *Crocidura* and *Neomys*, the marmot and the wood mouse *Apodemus* (*Alsomys*) appear in layer 17, but not in layer 18. Among the arboreal squirrels are a chipmunk (a representative of the taiga biotope group) and a flying squirrel that lives in tall birch forests. These fauna indicate that layers 18 and 17 formed under relatively warm conditions, consistent with the accumulation of these layers during the MIS 7 interglacial period (Fig. 10d).

The ecological composition of the small vertebrate community in layer 16 includes the shrews *Soricidae*, the mole and, among the arboreal squirrels, the chipmunk, the squirrel *Sciurus vulgaris* and the flying squirrel. The presence of the jerboa *Allactaga* in layers 17 and 16 suggests a disruption of the continuous grass cover and the emergence of local areas of dry steppe. The proportions of forest voles and steppe voles are variable in layer 16, whereas rock voles increase in abundance. The reappearance of the lemming *Lemmus*, a representative of the boreal biotope group, and the relatively low proportions of Siberian zokor and pikas *Ochotona* imply a deterioration in climatic conditions at

this time. The overall faunal composition of layer 16 suggests a relatively cold climate and fluctuating humidity, consistent with deposition near the end of the MIS 6 glacial period (Fig. 10d).

The faunal remains in layers 15–13 signal the next stage in development of the natural environment in the vicinity of Denisova Cave. The fossil assemblage in layer 15 is characterized by a consistently high proportion of forest voles, the presence of squirrels and flying squirrels, and the highest proportion of moles among insectivores. The relatively low proportions of Baraba hamster and Eversmann's hamster indicate a reduced abundance of cereal herbs due, most likely, to an increase in forested area, and the proportions of ground squirrels and flat-headed, narrow-headed and North Siberian voles (inhabitants of rocky or dry biotopes) likewise decrease. The distribution of root voles, the field vole *Microtus agrestis* and common voles among *Microtini* reflects wet conditions, and the presence of lemmings suggests the development of green moss associations. The increased proportions of Siberian zokor and frogs indicates favourable climatic conditions. In general, the composition of small vertebrates in layer 15 reflects a relatively warm and humid climate, consistent with deposition near the start of the MIS 5 interglacial period (Fig. 10d).

The fossil assemblage in layer 14 has a lower proportion of moles, red-backed voles and frogs, which we attribute to a reduction in forested area and a decrease in overall moisture supply. North Siberian voles, which prefer a boreal climate, increase in abundance, whereas the proportions of ground squirrels, Eversmann's hamster and Baraba hamster (typical representatives of the steppe biotope group) remain fairly stable. These environmental indicators indicate a slight decrease in temperature and humidity. The taxonomic composition of small vertebrates in layer 13 is similar overall to that of layer 14, but moles and forest voles *Clethrionomys* are slightly more abundant in layer 13, the wood mouse is present, and the proportions of Baraba hamster and mole voles are higher than in any other layer. The proportion of ground squirrels is also greater, indicating a larger area of grass forbs, and the stable population size of gray voles suggests the existence of meadow and floodplain associations. The similar ecological characteristics of the faunal communities in layers 14 and 13 indicate relatively warm climatic conditions, with the development of forest and meadow biotope groups during the period of accumulation of layer 13 near the end of MIS 5.

In the fossil assemblage from layer 12, the proportion of shrews falls to a minimum and the relative abundance of *Clethrionomys* and mole voles decreases by half. These changes indicate a significant reduction in areas of tall grass meadows and forests. The proportions of Eversmann's hamster and Baraba hamster, inhabitants of open landscapes, remain relatively stable, whereas the proportions of rock voles and steppe lemmings increase in layer 12, as do those of moles and Siberian zokor, both active diggers that prefer snowy winters with shallow freezing of the soil. Remains of arboreal squirrels were not recovered from this layer and, among the terrestrial squirrels, the proportions of ground squirrels and marmots increased, reflecting a steppe landscape with sparse grass. These data indicate a reduced area of forest and an expanded area of steppe, consistent with climatic cooling during the MIS 4 stadial (Fig. 10d)

The proportions of steppe lemming and Eversmann's hamster increase in layer 11, whereas those of red-backed, flat-headed and narrow-headed voles decrease. The remains of arboreal squirrels in this layer indicate the presence of local forests. The latter, combined with a high proportion of steppe lemmings, suggests the spread of forest-steppe landscapes during a relatively warm and dry period. The reduced proportions of moles and Siberian zokor, and a fall in the abundance of frogs, are also consistent with deposition of layer 11 during a period of relative aridity and mild temperature. We caution that layer 11 could not be stratigraphically subdivided during excavation and a complex formation history is indicated by the multiple age components obtained from optical dating (ages of approximately 80–40 and 30–20 ka) and ^{14}C dating (ages of more than 49 ka BP to as young as ~24 cal. ka BP), and by the recovery of artefacts characteristic of both the early and middle stages of the Upper Palaeolithic. Given these complications, we tentatively associate the upper part of layer 11 with MIS 2, but recognize that the lower part of this layer most likely corresponds to MIS 3.

SUPPLEMENTARY SECTION 2 | Optical dating of sediments in South Chamber (Zenobia Jacobs, Bo Li, Kieran O'Gorman and Richard G. Roberts)

Optical dating provides a means of determining burial ages for sediments and associated artefacts, fossils and other materials deposited at the same time as the sediments and not translocated subsequently[1,30–33]. The latter materials include microscopic fragments of bone and coprolite (fossilized faeces) that represent concentrated sources of ancient hominin and mammalian DNA at Denisova Cave[34]. The method is based on the time-dependent increase in the number of trapped electrons in mineral grains, most commonly quartz and potassium-rich feldspar (K-feldspar), induced by low levels of ionizing radiation from the radioactive decay of natural uranium, thorium and potassium in the deposits surrounding the mineral grains and inside the grains, and from cosmic rays. The time since the light-sensitive electron traps were last emptied can be estimated from determinations of the equivalent dose (D_e)—obtained from measurements of the optically stimulated luminescence (OSL) signal from quartz and the infrared stimulated luminescence (IRSL) and post-infrared IRSL (pIRIR) signals from K-feldspar—and the environmental dose rate for the mineral grains. The latter is estimated from the radioactivity of the sample and the material surrounding it to a distance of ~30 cm, plus the cosmic-ray contribution. The burial age of a sample, in calendar years since the date of collection, is obtained by dividing the D_e by the environmental dose rate.

In a previous study, we reported optical ages for 11 sediment samples from South Chamber[1]. A further 49 samples have been dated as part of the present study. For 55 of these 60 samples (92%), the D_e was estimated from measurements of individual grains of quartz and/or K-feldspar to gain from the benefits of single-grain dating[1,31,35]. These include the identification and rejection of grains with unsuitable OSL or pIRIR properties before D_e determination, and the analysis of D_e distributions to evaluate potential impacts of depositional and post-depositional processes on age estimation (e.g., insufficient exposure of grains to sunlight before deposition, mixing of grains after deposition). The OSL signal saturates at smaller doses than the pIRIR signal, so samples older than 100–150 ka were dated using only K-feldspar. As the pIRIR signal is less light-sensitive than the OSL signal, we made single-grain measurements on 35 samples using both minerals to check that the sediments had been exposed to sufficient sunlight (bleached) at the time of deposition. For five samples older than 250 ka (from layer 19), we used a multiple-aliquot regenerative-dose pIRIR procedure[36] to measure the large D_e values (>700 Gy).

2.1 Sampling strategy and sample descriptions

The locations of the four profiles sampled for optical dating in South Chamber are shown by the red lines labelled A–D in Fig. 1. South Chamber has a long, but interrupted, excavation history. The deposits at the entrance to the chamber were first excavated along the А, Б, В and Г gridlines to depths of ~4.5 m in 1999, 2000 and 2003. During a hiatus in excavation between 2003 and 2017, the profile along the Г gridline (Profile A) collapsed repeatedly, resulting in almost 1 m of sediment being redeposited as talus. The collapsed 2003 profile was cleaned back in 2012 (along the Д gridline in excavation squares 6 and 7) and, again, in 2016 prior to collection of sediment samples for optical dating[1]. The extent of collapse was less extensive than expected, perhaps due to the Pleistocene sediments remaining frozen for most of the year. The effect of freeze–thaw processes is evident from the flame structures and ice cracks that develop annually in the fine-grain and organic-rich Holocene deposits, and from the microscopic features (platy structures and rounded aggregates) observed in the underlying sediments, pdd-9 and pdd-12 (Supplementary Fig. 2). Profile A was cleaned back further in 2017 (almost to the E gridline in squares 6 and 7) prior to sample collection. Minimal collapse of the profile had occurred since 2016, such that the holes made when collecting sediment samples in 2016 were still visible in places.

Excavations resumed in 2017 along the Д, E and Ж gridlines to a depth of ~2 m, including ~0.5 m of Pleistocene deposit, and the З and И gridlines were excavated to a depth of ~2 m in 2018. In 2019, the Д and E gridlines were vertically extended to a depth of ~3.5 m, exposing more of the

Pleistocene deposits, and these sections were further excavated to a depth of ~5.5 m in 2020. The 2019 and 2020 excavations exposed, for the first time, a laterally continuous southeast profile of the lower deposits across the full width of South Chamber (Profile D). The same was achieved for the upper deposits in 2017 (Profile B) and 2018 (Profile C). Excavations have since taken place annually.

Sediment samples for optical dating were collected in 2012, 2016, 2017, 2018 and 2019 from Profiles A (n = 12), B (n = 18), C (n = 14) and D (n = 16). Our aims were to: (1) collect and date multiple samples from each of the layers identified during excavation of Profiles B, C and D; (2) sample across Profiles B and C to assess the impacts of phosphatization and sediment deformation on the stratigraphic integrity of the deposits, and develop a chronostratigraphy for these complex deposits; and (3) collect samples from Profile A to assess the age of the basal deposits (layer 19) and the stratigraphic integrity of the overlying dMP deposits, and establish a chronostratigraphy for these deformed deposits in the absence of a clear lithostratigraphy.

Sample locations were chosen during the day in consultation with the site archaeologist (M.B.K.) and site geologist (V.A.U.). The profiles were examined in white light to identify the layer boundaries and other stratigraphic features of interest, and pins were then used to mark the locations of individual samples prior to collecting them at night using subdued red light for illumination. The use of red light prevents bleaching of the light-sensitive OSL and pIRIR signals during sampling, but makes it difficult to distinguish between some layers, hence the pins. Each sample was assigned a unique sample code, following our previous approach[1] in which the sample code denotes the chamber (DCS for Denisova Cave South Chamber), the year of collection, and a unique identification number (e.g., DCS19-1); the number carries no stratigraphic information.

At night, the profile was first cleaned in and around the sampling location to remove sediment exposed to white light during the day. Samples were then collected using a ~2 cm-diameter coring device and sealed in zip-lock bags, which were wrapped in thick black plastic for transport to the Optical Dating Facility at the University of Wollongong in Australia. Samples were collected either by drilling holes horizontally into the profile (for the thicker layers) or by making smaller (thinner and wider) slots in the profile (for the thinner layers and for layers that contained many rocks and/or were difficult to control for angle of dip).

All holes and slots were inspected the following day (in white light) to check if any layers had been inadvertently cross-cut during sampling or any animal burrows had been intersected. Additional sediment samples were then collected from the back of each of the sample hole or slot for laboratory-based measurements of the prevailing moisture content and radioactivity. Sample holes and slots were enlarged, if necessary, to insert a 1 inch-diameter gamma-ray detector for in situ measurements of the gamma dose rates. Field notes were made about the presence of large limestone clasts and the abundance of rocks in and around the sample locations.

Individual sample locations in Profiles A–D are shown in Fig. 2. Samples collected in 2012, 2016, 2017, 2018 and 2019 are denoted by turquoise, yellow, green, pink and purple coloured squares, respectively, with the corresponding sample numbers enclosed within the squares.

Sample DCS19-1 was collected from a sediment pedestal located midway between Profiles A and D (red star in Fig. 1); a hominin parietal fragment (Denisova 22) was removed from the top of this pedestal in 2019. The sample consists of three parts (DCS19-1a, -1b and -1c), each approximately 3 cm thick and 7 × 10 cm in planform view, collected from directly beneath the fossil in order of increasing depth (Supplementary Fig. 3). The sediments exposed to white light during the day were removed to a depth of ~1 cm in red light at night, and the samples were then carefully excavated with a trowel, sealed in zip-lock bags and wrapped in black plastic.

2.2 Sample preparation and analytical facilities

All samples were prepared using standard procedures to extract quartz and K-feldspar grains for optical dating[32]. Carbonates and organic matter were first removed using HCl acid and H_2O_2 solution, respectively. The remaining sediment was then dried and sieved to obtain quartz grains of 180–212 μm diameter and K-feldspar grains of 150–180 or 180–212 μm diameter, depending on

availability. The quartz and K-feldspar grains were separated using sodium polytungstate solutions with specific gravities of 2.70, 2.62 and 2.58. The quartz grains were etched with 40% hydrofluoric (HF) acid for 45 min to remove the alpha-irradiated surface layer of each grain and dissolve any feldspar grains remaining in the quartz separates. The K-feldspar grains were treated with 10% HF acid for 40 min to remove the alpha-irradiated surface layer of each grain. The acid-etched quartz and K-feldspar grains were rinsed in HCl acid to remove any precipitated fluorides, and then sieved again.

D_e measurements were made using single-grain OSL procedures for all quartz samples, and either single-grain or multiple-aliquot pIRIR procedures for K-feldspars. Details of these D_e measurement procedures have been described previously (Supplementary Table 3 in [1]). All measurements were made on automated Risø TL-DA-20 luminescence readers equipped with focused green (532 nm) and infrared (830 nm) lasers for stimulation of individual grains of quartz and K-feldspar, respectively, and infrared (870 nm) light-emitting diodes for stimulation of multi-grain aliquots of K-feldspar[37]. The OSL and pIRIR signals were detected using Electron Tubes Ltd 9235QA photomultiplier tubes fitted with Hoya U-340 filters or Schott BG-39 and Corning 7-59 filters, respectively. Aluminium discs drilled an array of with 100 holes, each 300 µm in diameter and depth, were used for the single-grain measurements. For the multi-grain aliquot measurements, grains were placed in the centre of a 9.8 mm-diameter stainless steel disc as a 5 mm-diameter monolayer, using 'Silkospray' silicone oil as an adhesive. Beta irradiations were delivered inside the Risø readers using $^{90}Sr/^{90}Y$ sources calibrated using several known-dose gamma-irradiated quartz standards, including various batches of Risø calibration quartz taking into account recent dose corrections[38]. We also accounted for spatial variations in beta dose rate to individual grain positions on the single-grain discs, based on measurements made using the same gamma-irradiated quartz standards. A solar simulator (Dr Hönle model UVACUBE 400) was used to conduct bleaching tests in the laboratory.

2.3 Equivalent dose (D_e) estimation

D_e estimation for quartz

Thirty-five samples from South Chamber were measured using a total of 106,500 individual quartz grains. Aberrant grains were rejected using the same quality-assurance criteria as previously[1]. Supplementary Data 7 lists the numbers of individual grains measured, rejected and accepted for D_e determination for each of the samples, and the reasons for rejection.

We applied two methods to determine D_e values for quartz grains: the conventional single-aliquot regenerative-dose (SAR) procedure[39,40] and the L_nT_n method[41,42]. Both are single-grain methods and use the same L_n, T_n, L_x and T_x values estimated from the first 0.22 s of OSL decay, with the mean count rate recorded over the last 0.3 s of stimulation subtracted as background.

The conventional SAR procedure was used for 29 samples, following the approach described previously[1]. This involved constructing single-grain dose-response curves (DRCs) from measurements of the L_x and T_x OSL signals at each regenerative dose, and fitting a general-order kinetics function[43] to the sensitivity-corrected L_x/T_x ratios. The sensitivity-corrected natural OSL signal (L_n/T_n ratio) for each accepted grain was then projected onto its corresponding DRC to estimate the D_e value by interpolation.

The L_nT_n method was used for the six samples shown in italics in Supplementary Data 7. With this method, the distributions of least-squares (LS-) normalized L_n/T_n ratios (rather than the D_e values) are analyzed for all accepted grains, including those with L_n/T_n ratios identified as saturated (criteria 6 and 7 in Supplementary Data 7). These distributions were then interpreted and the data combined in the same way as for the single-grain D_e values obtained using the conventional SAR procedure. The L_nT_n method relies on all accepted grains having a common DRC—a so-called 'standardized growth curve' (SGC). Individual quartz grains, however, have significantly different DRC shapes and characteristic saturation doses (D_0 values), even quartz grains from the same sample (see Supplementary Figure 1 in [1]), making it necessary to sort the accepted grains into groups according to their DRC shapes[44]. We achieved this by comparing the ratios of the L_x/T_x values measured at two regenerative doses for each of the accepted grains, one dose close to the maximum expected D_e value

of the sample and the other in the lower dose region where most grains have a similar DRC shape[45]. We used the finite mixture model (FMM) to statistically determine the optimal number of groups of grains with similar DRC shapes, and the probability of a grain belonging to a specific group of grains with similar ratios of L_x/T_x values; the number of groups is sample dependent. The LS-normalization procedure[41] was then applied to each group of grains, and group-specific SGCs were constructed by fitting the LS-normalized L_x/T_x values with a general-order kinetics function. The weighted mean LS-normalized L_n/T_n ratio for each group—determined using the FMM, the central age model (CAM)[39,46], or the CAM after removing statistical outliers identified using the normalized median absolute deviation (nMAD CAM)—was then projected onto the group-specific SGCs to estimate the corresponding D_e values (Supplementary Data 10). The latter values were validated by comparing the D_e values derived from the SGCs with those obtained for the same (non-saturated) grains using the individual DRCs constructed as part of the conventional SAR procedure. The group-specific D_e values for each sample were then combined to estimate a final weighted mean D_e value for age determination.

D_e estimation for K-feldspar

We applied the same three methods (Methods A, B and C) and procedures described and tested previously[1] to determine the D_e values for K-feldspar grains from South Chamber and, for consistency, assigned samples to the same three groups (Groups A, B and C).

Methods A and B involve application of a two-step SAR pIRIR procedure to individual K-feldspar grains[47,48]. Single-grain pIRIR signals were measured at 275°C for 2 s using the infrared laser for stimulation, following stimulation at a high temperature (200°C) for 200 s using infrared light-emitting diodes[49]. A preheat of 320°C for 60 s was applied prior to measurement of the natural (L_n), regenerative (L_x) and test dose (T_n, T_x) signals, and an infrared bleach at 325°C for 100 s was given at the end of each measurement cycle. Group A consists of the youngest samples, which were measured using a single-grain SGC procedure[50] (Method A). In this method, a SGC was first constructed using the L_x/T_x ratios obtained for the accepted grains of 44 samples from Denisova Cave[1]. D_e values were then estimated for individual grains by projecting their re-normalized natural signals (L_n/T_n ratios) onto the SGC to obtain a distribution of single-grain D_e values. The D_e distributions were then interpreted and combined using an appropriate statistical model. Samples of intermediate age (Group B) were measured using the single-grain L_nT_n method (Method B)[41,42]. In this method, we first interpreted the distribution of re-normalized L_n/T_n ratios for all grains measured from a particular sample (including grains with L_n/T_n ratios consistent with, or higher than, the saturation level of the corresponding sample DRC), before combining their ratios using an appropriate statistical model and projecting the weighted mean re-normalized L_n/T_n ratio onto the SGC to estimate the sample D_e.

The oldest samples (Group C) were measured using a multiple-aliquot regenerative-dose procedure[36] (Method C). This method is based on the construction of a series of SGCs using the re-normalized L_x/T_x signals from multiple groups of aliquots stimulated at a range of temperatures. The natural and corresponding test dose signals (L_n and T_n), a regenerative dose signal (L_r) and the subsequent test dose signal (T_r) are then measured for a number of individual aliquots, which are bleached in the solar simulator for ~4 hr at the end of each test dose measurement. The single-aliquot L_n/T_n ratios are then re-normalized using their corresponding L_r/T_r ratios, and the weighted mean re-normalized ratio and associated error (calculated for each sample using the CAM) are projected on the MAR SGCs to estimate the corresponding sample D_e values. In the present study, we used the multiple- and single-aliquot SGCs validated in our earlier study[1]. Of the five samples measured using Method C, two (DCS16-6 and DCS19-14) were also measured using Method B.

Residual dose and fading tests were each conducted on individual K-feldspar grains from two samples from Denisova Cave in our previous study[1], in which we showed that the pIRIR signals were bleached to a negligible level and faded negligibly. Accordingly, we made no residual-dose adjustments to the measured D_e values for any of the samples in the present study, and did not apply any fading corrections to the calculated pIRIR ages.

The number of K-feldspar grains measured, rejected and accepted are listed in Supplementary Data 8 for each of the samples measured using single grains, along with the reasons for rejection. Also shown for each sample are the respective Group and method of D_e estimation and, where applied, the 'threshold' T_n signal intensity above which a 'plateau' in D_e values or LS-normalized L_n/T_n ratios was attained.

D_e and L_n/T_n distributions

Supplementary Data 9 contains information about the number of grains measured and used for D_e determination, the overdispersion (OD) values estimated for each of the D_e distributions, and the final D_e values ± 1σ uncertainties for each sample. Samples are organized by profile and by stratigraphic layer within each profile, with coloured shading used to denote the K-feldspar Group assignment. Group A, B and C samples (measured using Methods A, B and C, respectively) are shaded in blue, red and green, respectively. Quartz samples were measured using the single-grain conventional SAR procedure, except for the six samples measured using the L_nT_n method (italicized in Supplementary Data 7).

Supplementary Fig. 12 shows radial plots of the single-grain D_e distributions for all quartz samples measured using the conventional SAR procedure and the single-grain D_e or LS-normalized L_n/T_n distributions for all K-feldspar samples measured using Methods A or B. For the six quartz samples measured using the L_nT_n method, the Group-specific single-grain OSL data are presented in Supplementary Figs. 13–18; and for the five K-feldspar samples measured using Method C, the re-normalized L_n/T_n distributions are displayed in Supplementary Fig. 19.

For each sample, the individual D_e or LS-normalized L_n/T_n values were combined using one of four statistical models to estimate the weighted mean D_e value for age determination (Supplementary Data 9). We based the choice of model on the distribution pattern and extent of overdispersion of the D_e or LS-normalized L_n/T_n values, the sample-specific context (including depositional and post-depositional factors) and other relevant considerations, such as field and micromorphological observations[31]. The following models were used:

1. Central age model (CAM): This model assumes that the D_e or LS-normalized L_n/T_n values for all grains in a sample are centred on a common value, with the estimated standard error taking into account any overdispersion[39,46].
2. nMAD CAM: For this model, the normalized median absolute deviation (nMAD) is first used to identify any statistically significant outliers in the D_e or LS-normalized L_n/T_n distributions, using 1.4826 as the appropriate correction factor for a normal distribution and a cut-off value of 2[42]. After outliers have been removed, the remaining values are combined using the CAM.
3. Finite mixture model (FMM): This model is applied to D_e or LS-normalized L_n/T_n distributions that consist of two or more discrete components. The number of components, the proportions of grains in each component, and the corresponding mean D_e or LS-normalized L_n/T_n value (and associated standard error) of each component is estimated by the FMM[46,51]. The optimal combination of the number of components and extent of overdispersion is determined iteratively from the maximum log-likelihoods and Bayes information criterion (BIC) values obtained from fitting the model[46].
4. Minimum age model (MAM): This model assumes that the individual D_e or LS-normalized L_n/T_n values are derived from a truncated normal distribution, with the lower truncation point corresponding to the mean D_e or LS-normalized L_n/T_n value of the population of grains associated with the target event[39,46]. In this study, the latter corresponds to the population of most fully bleached grains in the distribution. We used the MAM to estimate the final D_e value for three quartz samples (DCS18-14, DCS19-4 and DCS19-12) and one K-feldspar sample (DCS18-1). We interpret the latter distribution as evidence of heterogeneous bleaching of the pIRIR signal, based on a comparison with the distribution obtained for the quartz grains in this sample using the more light-sensitive OSL signal. For all four samples, we added an OD value of 30% in quadrature to each of the single-grain D_e uncertainties before running the model[46].

All data analyses—including curve fitting, SGC construction, LS-normalization, D_e and error estimation, and statistical analysis of D_e values—were made using the functions implemented in R packages *numOSL*[52,53] and *Luminescence*[54,55].

2.4 Environmental dose rate determination

The total environmental dose rate consists of contributions from beta, gamma and cosmic radiation sources external to the grains, plus the internal dose rate derived from radioactive decay of potassium (^{40}K) and rubidium (^{87}Rb) within sand-sized grains of K-feldspar and uranium (U) and thorium (Th) inclusions inside sand-sized grains of quartz. For both minerals, we assumed that the present-day radionuclide activities and dose rates have prevailed throughout the period of sample burial. When beta and gamma dose rates are measured using emission-counting methods, as is the case in this study, errors in the total dose rate of more than 2–3% are unlikely to arise from most time-dependent disequilibria in the U and Th decay chains[31,56,57].

External beta dose rates

We estimated the beta dose rates in the laboratory from low-level beta counting of dried, homogenized and powdered sediment samples using a Risø GM-25-5 multi-counter system[58]. Three sub-samples were measured for each sample. Procedures described previously[59] were used to prepare and measure the samples, analyze the data obtained, and calculate the beta dose rates and their uncertainties. Allowances were made for the effect of sample moisture content, grain size and HF acid etching on beta-dose attenuation[1]. Supplementary Data 9 lists the external beta dose rates for all samples, which range from 0.61 ± 0.04 Gy/ka (DCS18-1) to 2.42 ± 0.15 Gy/ka (DCS18-5).

Samples collected from the right-hand side of Profile D have generally lower beta dose rates (0.78–1.05 Gy/ka) than those from left-hand side of Profile D (0.98–1.43 Gy/ka) and samples from Profile A (0.81–1.48 Gy/ka), Profile B (0.86–1.87 Gy/ka) and Profile C (0.61–2.42 Gy/ka). The highest beta dose rates are for samples from layer 19 at the base of the stratigraphic sequence (>1.34 Gy/ka) and from the phosphatized pdd-9 sediments in Profiles B and C (>1.87 Gy/ka). The latter dose rates reflect the very high K concentration of ~3%, which may be due to the post-depositional formation of taranakite, a phosphate mineral high in K[3,4].

We also measured the U, Th and K contents in 52 samples using inductively coupled plasma mass spectrometry (ICP-MS) for U and Th and inductively coupled optical emission spectroscopy (ICP-OES) for K. The ICP-MS and -OES measurements were made independently in a commercial laboratory (Intertek Genalysis). The dry and unattenuated beta dose rates calculated from elemental concentrations of U, Th and K by ICP-MS/OES are compared in Supplementary Fig. 20a with those measured directly using the GM-25-5 beta counter. The latter counts all beta emissions from the U and Th decay chains and from the radioactive decay of ^{40}K, whereas the dose rates obtained from ICP-MS data are based on measurements of the parental U and Th concentrations and assume secular equilibrium in the decay chains.

The beta dose rates derived from the ICP-MS/OES data are mostly higher than those measured by beta counting, with a mean ratio and standard deviation of 1.08 ± 0.07 for all samples; individual ratios range between 0.95 ± 0.03 (DCS18-2) and 1.29 ± 0.05 (DCS16-1). The mean offset of ~8% is larger than that of ~4% obtained in a previous comparison[1] made using 56 samples from Main (n = 26), East (n = 24) and South (n = 6) Chambers. The size of the offset in the present study depends, however, on the profile sampled in South Chamber. The beta dose rates for samples from Profiles C and D are consistent with an offset of ~4%, whereas samples from Profiles A and B have larger differences: the mean ratio and standard deviation for samples from Profiles A, B, C and D are 1.13 ± 0.05 (n = 18), 1.15 ± 0.06 (n = 6), 1.03 ± 0.06 (n = 14) and 1.05 ± 0.04 (n = 14), respectively. The six samples from South Chamber included in the earlier comparison (DCS16-1 to DCS16-6)[1] were collected from Profile A, and have ratios of 1.07–1.29. The mean ratio and standard deviation of 1.13 ± 0.08 for these samples is consistent with the ratios of 1.09–1.17 and mean ratio of 1.13 ± 0.03 obtained for samples collected from this profile in 2017.

To further investigate the differences between the beta dose rates determined from ICP-MS/OES and beta counting, we measured three sub-samples of 15 samples (DCS17-1 to -15) on a different GM-25-5 beta counter. The mean ratio and standard deviation of the beta dose rates derived from the two data sets is consistent with unity at 2σ (1.03 ± 0.02), so the instrument used is not the cause of these differences.

We also considered the possible effect of differences in the beta dose rate composition of the South Chamber samples and the reference standard (Nussi)[59] used for both beta counters to calculate the GM-25-5 beta dose rates[60]. We calculated the relative contributions of K, U and Th to the beta dose rate using the values obtained from the ICP-MS/OES data. K contributes, on average, ~71% of the total beta dose rate, and U and Th each contribute ~19% and ~10%, respectively, with little variation between samples. The Nussi standard has a dry and unattenuated beta dose rate (1.5399 Gy/ka) similar to that of most samples in this study, but with slightly different relative contributions from K, U and Th (60, 27 and 13%, respectively). These differences are insufficient, however, to explain the differences in beta dose rates determined from ICP-MS/OES and beta counting. We also note that samples with the highest relative contribution from K (75–82%) have beta dose rate ratios (ICP-MS/OES to beta counting) of 1.01–1.09, whereas the two samples with the lowest relative contribution from K (64% and 68%)—the most similar to Nussi—have beta dose rate ratios of 1.20 and 1.15. This result argues against compositional differences between the Nussi standard and the South Chamber samples being the primary cause of the differences in beta dose rate determined from ICP-MS/OES and beta counting. Furthermore, if this were a factor then samples from all four profiles should be affected similarly, whereas much smaller differences between the two data sets were obtained for samples from Profiles C and D.

Another contributory factor may be disequilibrium in the U decay series. ICP-MS provides an estimate of the present-day concentration of parental U in the sample. In the ^{238}U decay series, only ~39% of the beta dose rate derives from the top of the chain (^{238}U to ^{234}U). Most of the beta dose rate (~61%) is derived from the decay of radionuclides much further down the chain[61]. GM-25-5 beta counting measures all beta particles emitted by the U decay series, including beta emissions from radionuclides lower down the chain. A discrepancy between the two methods may, therefore, reflect disequilibrium in the U decay series. A common form of disequilibrium is diffusion of radon (^{222}Rn) gas[31]; losses of 25%, for example, have been reported for sediments deposited in a limestone cave in Australia[57]. To test whether ^{222}Rn loss could account for some of the beta dose rate differences between the ICP-MS/OES and beta counting data sets for the South Chamber samples, we reduced the fractional contributions of ^{222}Rn and radionuclides lower down the chain by 20% and recalculated the ICP-MS/OES beta dose rates. This adjustment was sufficient to account for the 3–5% difference in beta dose rate for samples from Profiles C and D, but was insufficient to explain the 13–15% differences for samples from Profiles A and B; even assuming a ^{222}Rn loss of 90% did not result in a beta dose rate ratio consistent with unity for the ICP-MS/OES and beta counting data sets. More complex issues may, therefore, be at play in Profiles A and B. These are likely associated with the sedimentology of the deposits and the impacts of hydrological processes and, especially in Profile B, post-depositional phosphatization. Deposits on the left-hand side of the central ridge of spalled rocks appear to have been affected to a greater extent than those on the right-hand side.

We consider the most reliable estimates of beta dose rate to be those obtained from GM-25-5 beta counting, as these as take into account the state of (dis)equilibrium in the U and Th decay chains at the time of sample collection. Assuming that present-day conditions have existed throughout the period of sample burial is unlikely to give rise to errors in the total dose rate of more than a few percent[31,56,57].

Beta dose rates determined from GM-25-5 beta counting or ICP-MS/OES measurements represent the average for 'bulk' samples composed of several grams of homogenized sediment. In a recent study[62], we measured two samples from Main Chamber using a Timepix hybrid pixel detector and obtained sample-average beta dose rates consistent with those determined by beta counting or ICP-MS/OES measurements. The Timepix measurements also revealed spatial heterogeneity in beta dose rates at sub-millimetre resolution, with radioactive 'hot' and 'cold' spots giving rise to grain-to-

grain differences in beta dose rate. A comparison of the latter with the spread in single-grain D_e values for these two samples showed that the extent of the scatter in D_e values, and shape of the D_e distributions, could be fully or partly explained by sub-millimetre differences in beta dose rate and other known sources of variability in single-grain D_e values[41,62].

Gamma dose rates

As gamma rays have a range of ~30 cm in most sediments and rocks, we measured the in situ gamma dose rate at each sample location (except for DCS19-1) using a portable gamma-ray spectrometer, to allow for spatial heterogeneity in the surrounding gamma radiation field. Counts were collected for 1 hr with a NaI(Tl) detector (1 inch in diameter) and a few samples were measured for longer periods of time. As in our earlier study[1], the detector was calibrated using the concrete blocks at Oxford University and the gamma dose rates were determined using the 'threshold' technique, which provides an estimate of the combined dose rate from gamma-ray emitters in the U and Th decay chains and from ^{40}K.

Supplementary Data 9 lists the gamma dose rates for all samples, which range from 0.28 ± 0.02 Gy/ka (DCS18-1 and DCS19-9) to 0.99 ± 0.06 Gy/ka (DCS18-6). In general, samples with the lowest gamma dose rates are located closest to the cave walls and individual large rocks or in areas with large quantities of smaller rocks, reflecting the much lower radioactivity of the limestone bedrock compared to the cave sediment. Although the deposits range from clast-supported (e.g., layer 11 in Profiles B and C) to texturally homogeneous silty clay (e.g., layer 19 in Profile A), most have compositions between these extremes and contain scattered clasts of various sizes. The amount of smaller rock shatter (grus) increases towards the cave walls, likely due to mechanical weathering of the limestone as a result of freeze–thaw action over long periods of time.

To illustrate the effect of the presence of limestone on gamma dose rates, we calculated the latter from ICP-MS/OES measurements of K, Th and U concentrations for 45 samples and compared these estimates with those obtained by in situ gamma spectrometry (Supplementary Fig. 20b). The two approaches give similar gamma dose rates for only three of these samples (mean ratio of 1.04 ± 0.01), all from the relatively homogeneous layer 19 sediments at the base of Profile A. The in situ gamma dose rate estimate for sample DCS19-14, from layer 19 in Profile D, is approximately one-third lower than its ICP-MS/OES counterpart and the in situ estimates for the other layer 19 samples. Its proximity to a large boulder (Fig. 2d) illustrates the dilution effect of limestone on the gamma dose rate of the sediment. For the other samples, differences in the gamma dose rate estimates range between ~14% for DCS17-14 (collected from the base of the dMP deposits in Profile A; Fig. 2c) to ~290% for DCS17-4 (collected from the clast-supported layer 11 deposits on the left-hand side of Profile B; Fig. 2a). In situ gamma spectrometry measurements are essential in complex depositional contexts such as these, as the effect of the limestone rocks on the gamma dose rate cannot be modelled without knowing the distribution of rocks in all three dimensions. Only two samples, from layers 5 (DCS18-7) and 8 (DCS18-8) in Profile C (Fig. 2b), have in situ gamma dose rates greater than the ICP-MS/OES estimates. Both of these samples are from the organic-rich Holocene deposits.

Sample DCS19-1 was collected from the cave floor, directly beneath Denisova 22. We could not obtain a meaningful in situ gamma spectrometry measurement for this sample because most of the sediments within its gamma radiation field had already been excavated at the time of sampling. Instead, we estimated the gamma dose rate using a combination of thick-source alpha counting and GM-25-5 beta counting to derive the K, Th and U contents and corresponding dose rates. The gamma dose rate so obtained is statistically consistent with that derived from ICP-MS/OES (ratio of 1.03 ± 0.06). We assessed the extent of spatial heterogeneity in the gamma radiation field at the time of sample collection. The sampled sediment is relatively homogeneous in texture, but a large slab of limestone extended into and below the sediment baulk from which DCS19-1 was collected. Almost 90% of the gamma dose rate delivered to a sample originates from the nearest 10 cm of surrounding deposit[61], and the limestone slab occupied only the outer 10 cm of the gamma sphere on one side of the sample. We therefore consider its impact on the gamma dose rate to be negligible and used the

gamma dose rate estimated from thick-source alpha counting and GM-25-5 beta counting for all three sub-samples of DCS19-1.

Cosmic-ray dose rates

We used published equations[63] to estimate the cosmic-ray dose rates, taking into account the geomagnetic latitude (40.6°) and altitude (~670 m) of Denisova Cave and the density and thickness of sediment overburden and limestone bedrock shielding South Chamber. The latter greatly reduces the cosmic radiation delivered to the cave deposits. We used a cosmic-ray dose rate of 0.030 ± 0.005 Gy/ka for all samples, which represents a trivial fraction of the total dose rate (<2% for quartz and <1% for K-feldspar, on average), owing to the comparatively high external beta and gamma dose rates and the substantial internal dose rate for K-feldspar grains.

Moisture content

The external dose rate components (beta, gamma and cosmic) are affected by the moisture content of a sample and the material surrounding it (up to a distance of a few millimetres for beta particles and ~30 cm for gamma rays). We measured the moisture content of each sample in the laboratory to obtain an estimate of the field value at the time of sample collection. These values range from 12% to 42%, and are listed in brackets in Supplementary Data 9.

For the gamma dose rates, which were measured in situ, we made no adjustment for possible long-term changes in moisture content, as the cave deposits are frozen for most of the year. Seasonal and longer-term variations are, therefore, considered unlikely to be substantial. The beta dose rates, however, are determined from laboratory measurements of dried samples, so it necessary to apply a correction for moisture content. We used the field values as a guide to the average long-term moisture content of each sample and applied a relative error of ± 25% (at 1σ) to accommodate any likely variations over the period of sample burial.

The samples from Profile A (20–29%) were collected in 2017 and have field values very similar to, or slightly drier than, the values obtained in our earlier study[1] for samples collected in 2012 and 2016 from this profile (21–39%). We therefore adopted the same approach as previously and assigned a moisture content of 25 ± 5% to all samples from Profile A.

Samples from Profiles B and C (collected in 2017 and 2018, respectively) have a wider range of moisture contents (12–42%), reflecting marked spatial differences in the sedimentological characteristics within and between these two profiles and compared to the deposits exposed in Profiles A and D. Layers 5, 7, 8, pdd-9 and pdd-12 are silty in texture, in places almost spongy, whereas layer 11 consists of more rocks than sediment, resulting in better drainage. The general increase in abundance of clasts towards the cave walls is accompanied by a decrease in moisture content. We consider this lateral variability in moisture content, therefore, to be a true reflection of spatial differences. Accordingly, we adopted moisture contents of 35 ± 7% for samples from layers 5, 7 and 8, 30 ± 6% for samples from pdd-9, 25 ± 5% for samples from layer 12 and pdd-12, and 15 ± 4% for samples from layer 11.

Samples collected from Profile D in 2019 (and DCS19-1 from the sediment pedestal) have field values of 21–37%, which are similar to those of samples from Profile A. Samples with the lowest moisture contents are typically from the rockiest parts of the profile, whereas layer 14, in particular, is very clay-rich and moist. We assumed long-term moisture contents of 25 ± 5% for layers 12 and 15–17, 30 ± 6% for samples from layers 13 (including DCS19-1) and 19, and 35 ± 7% for samples from layer 14.

The calculated OSL and pIRIR ages are relatively insensitive to variations in the assumed long-term average moisture content, increasing (or decreasing) by ~1% for each 1% increase (or decrease) in moisture content for quartz and about half that for K-feldspar.

Internal dose rates

Quartz is essentially inert, but may contain trace amounts of U and Th that emit alpha and beta particles. We assumed an internal dose rate of 0.03 ± 0.01 Gy/ka for all quartz samples, based on U

and Th concentrations of 0.15 ± 0.03 and 0.35 ± 0.07 μg/g, respectively, and an alpha-efficiency *a*-value of 0.04 ± 0.01[1].

Unlike quartz, K-feldspar grains have a substantial internal beta dose rate due to the radioactive decay of ^{40}K and ^{87}Rb inside the grains. In our earlier study[1], we measured the internal K concentrations of 60 individual K-feldspar grains from two samples (one each from Main and East Chambers) using wavelength-dispersive X-ray spectroscopy. In the present study, we measured the whole-of-grain K concentrations for individual feldspar grains from two of the South Chamber samples, DCS17-3 (n = 62) and DCS17-6 (n = 54), using quantitative evaluation of mineral–energy dispersive spectroscopy (QEM-EDS). Only grains accepted for D_e determination were included in this analysis. Sample preparation, measurement and analytical procedures are described elsewhere[64]. We consider these procedures appropriate for the South Chamber samples because individual feldspar grains from Ust'-Karakol 1, an open-air site located ~2 km southeast of Denisova Cave, were used to establish the QEM-EDS calibration curve and correction factors. Feldspar grains at Denisova Cave are derived from the same geological sources as those at Ust'-Karakol 1, which exhibit perthitic textures and have orthoclase/microcline and albite phases with reasonably strict ranges of compositions[64].

Supplementary Fig. 20c shows the QEM-EDS maps for a feldspar grain from DCS17-3 (left-hand panel) and two grains from DCS17-6 (middle and right-hand panels), which illustrate the range of compositions observed. Almost all of the grains were dominated by orthoclase/microcline (blue phases) with minor proportions of fine albite formed by exsolution (green phases). Only one of the 116 grains contained a considerable (~56%) proportion of albite, and some grains had minor inclusions of quartz (beige phases) and mica (red phases). Individual grains were composed, on average, of ~91% orthoclase/microcline, ~7% albite, ~1% celadonite–muscovite and <1% quartz; ~1% of pixels remained unclassified (Supplementary Data 11).

Supplementary Fig. 20d shows the whole-of-grain average K concentrations plotted against the T_n signal intensities for all 116 grains. Only one grain has a K concentration of less than 9.4 wt%, rendering it low-K according to the classification of [64]. The other 115 grains are K-rich and have arithmetic and T_n-weighted mean K concentrations of 12.5 and 12.4 wt%, respectively (DCS17-3) and 12.4 and 12.5 wt%, respectively (DCS17-6). Combining both data sets yields an arithmetic mean K concentration and standard deviation (at 1σ) of 12.4 ± 0.9 wt%, which we used to calculate the internal dose rates for the K-feldspar grains in this study. The 2σ range (10.6–14.2 wt%) incorporates the whole-of-grain average K concentrations of 113 of the grains (97%) and the sample-average K concentration of 12.8 ± 0.5 wt% obtained for the Main and East Chamber samples using wavelength-dispersive X-ray spectroscopy[1].

Since the individual K-feldspar grains accepted for D_e determination are dominated by orthoclase/microcline, we considered it appropriate to also account for the dose rate from ^{87}Rb but assumed that U and Th concentrations are negligible. Using the measured K concentration of 12.4 ± 0.9 wt% and an assumed Rb concentration[65] of 400 ± 100 μg/g yields internal dose rates of 0.714 ± 0.058 and 0.838 ± 0.067 Gy/ka for grains with diameters of 150–180 and 180–212 μm diameter, respectively; we used these values for all K-feldspar samples.

Total environmental dose rates

Supplementary Data 9 lists the external beta and gamma dose rates for each sample, in addition to the internal dose rates, total dose rates, moisture contents and grain sizes used for D_e determination. Samples are presented for each profile and listed in stratigraphic order (top to bottom).

The total dose rates for samples measured using K-feldspar grains vary by a factor of 2.4, from 1.76 ± 0.08 Gy/ka (DCS18-1) to 4.26 ± 0.17 Gy/ka (DCS18-5). For the 35 samples measured using quartz grains, the total dose rates vary by a factor of 3.6, from 0.96 ± 0.05 to 3.46 ± 0.16 Gy/ka for the same two samples. We identify three main clusters in the data set of 60 samples. Forty-nine of the samples (82%) have total dose rates that vary by only 59% for K-feldspar and 53% for quartz. The other two clusters have systematically higher total dose rates: six samples from each of pdd-9 and layer 19. The latter samples were measured only using K-feldspar grains, which have total dose rates

of between 2.68 ± 0.11 and 3.18 ± 0.12 Gy/ka. The samples from pdd-9 have total dose rates that range from 3.43 ± 0.14 to 4.26 ± 0.17 Gy/ka for K-feldspar, and from 2.74 ± 0.12 to 3.46 ± 0.16 Gy/ka for quartz: these dose rates reflect the very high K concentrations (~3%) associated with the post-depositional formation of phosphates that likely include taranakite, which is rich in K.

Supplementary Data 12 lists the average percentage contribution and standard deviation of each dose rate component that contributes to the total dose rates for the quartz and K-feldspar grains. For the quartz samples, the total dose rate is dominated, on average, by the external beta dose rate (~66%), followed by the gamma dose rate (~29%) and the cosmic-ray and internal alpha dose rates (<4% combined). Two different grain sizes of K-feldspar were measured. For the largest grains (180–212 μm in diameter), the external beta dose rate accounts for an average of ~45% of the total dose rate, followed by the internal beta dose rate (~34%), the gamma dose rate (~20%) and the cosmic-ray dose rate (~1%). The proportions are similar for the smaller K-feldspar grains (150–180 μm in diameter), with the internal beta dose rate accounting, on average, for ~31% of the total dose rate.

Importantly, the substantial internal beta dose rate of K-feldspar grains is not affected by spatial heterogeneity in the distribution of radionuclides in the surrounding deposits or by time-dependent changes in external beta dose rate associated with, for example, variations in moisture content and any disequilibria in the U or Th decay series. As a result, the spread in K-feldspar D_e values is less affected by variations in external beta dose rate than are quartz D_e values. When combined with accurate and precise estimates of internal K concentration, optical dating of K-feldspar grains is of particular benefit in depositional environments with complex histories, such as South Chamber. Furthermore, as K-feldspar and quartz grains are affected to differing extents by any spatial or temporal variations in external beta dose rate, increased confidence in the reliability of optical age estimates can be inferred from similar ages being obtained for both minerals in the same sample.

2.5 Age estimates and Bayesian models A and B

The final optical ages for all samples are listed in Supplementary Data 9, together with the associated D_e and dose rate estimates and other supporting data. Uncertainties on the ages are given at 1σ (the standard error of the mean) and were estimated by combining in quadrature all known and estimated sources of random and systematic error. Ages are listed separately for Profiles A–D and in stratigraphic order within each profile.

Paired single-grain OSL ages for quartz and single-grain pIRIR ages for K-feldspar were obtained for 35 samples, of which 28 had D_e distributions from which reliable ages (ranging between ~5 and ~150 ka) could be obtained for comparison. For the remaining 7 samples (most from Profile C), one or both minerals had widely scattered distributions of D_e or LS-normalized L_n/T_n values, from which reliable ages could not be calculated, or the distributions consisted of multiple, discrete D_e components with no dominant component. Although the D_e values for quartz and K-feldspar have largely independent errors, the two minerals have several shared dose rate components and errors. The shared error terms need to be taken into account when comparing the ages obtained from the two minerals for the same sample, and if they are then combined to estimate a weighted mean age. Accordingly, in the comparison of paired OSL and pIRIR ages and when calculating the weighted mean ages, all shared errors are omitted and only the total random error associated with the D_e values and the error associated with the internal beta dose rate are included.

The 28 pairs of OSL and pIRIR ages are presented in Supplementary Fig. 21a. Each pair of ages is consistent at 2σ, and the mean ratio for all 28 pairs is 1.016 with a standard deviation of 0.078 and a standard error of 0.015. The ratios are randomly spread around the 1:1 line, suggesting no systematic trend over the 5–150 ka interval. We estimated the weighted mean age of these 28 samples using the CAM, weighting the ages by their total random-only errors (Supplementary Data 13). For all three chambers, 71 samples have paired single-grain OSL and pIRIR ages (Supplementary Fig. 21b). Each pair of ages is likewise consistent at 2σ, exhibit no systematic trend with age, and yield a mean ratio consistent with unity (0.998 ± 0.010). As the pIRIR traps do not bleach as rapidly or completely as those responsible for the fast-decay component of the OSL signal[1,31,32,66,67], the consistency between

the paired ages implies that the sediments in Denisova Cave were bleached sufficiently by sunlight at the time of deposition to reset both dating signals, at least over the last 150 ka.

Two Bayesian statistical age models—models A and B—were constructed for the South Chamber samples using the OxCal platform (OxCal 4.4.4)[68,69]. Ages for 31 samples (52% of the total number of samples dated, n = 60) were included in model A, and ages for 50 samples (83%) in model B (Supplementary Fig. 22). Ages were input as C_dates in calendar years before AD 1950 with an associated 1σ error; these are the so-called likelihood estimates; only the unshared errors (shown in brackets in Supplementary Data 9) were included in the model. Samples included in both models had D_e distributions dominated by a single population of grains (after removing statistically significant outliers identified using the nMAD) or that consist of two or three discrete D_e components, one of which contains more than 70% of the grains (Supplementary Data 9). Model A included only ages for samples from stratigraphic layers that could be clearly defined and placed securely in the lithostratigraphic sequence of sediment layers; this excludes samples from the dMP deposits in Profile A (Fig. 2c). Model B included not only the ages in model A but also ages that could be assigned with confidence to an archaeological phase (but not to a specific stratigraphic layer); this includes samples from the dMP deposits in Profile A with D_e distributions dominated by a single population of grains or by a component that contains at least 70% of the grains (Supplementary Fig. 22e,f).

In Bayesian analysis, the likelihood estimates are analyzed with respect to prior information, and the modelled ages (the posterior distributions) are estimated. We used the lithostratigraphy as prior information for model A and the archaeological phases as prior information for model B. Each of these sediment layers or archaeological phases was modelled as a *Phase*, in which the measured ages are assumed to be unordered and uniformly distributed; any mixing within a sediment layer or archaeological phase will, therefore, not affect the model. *Boundaries* were placed at the start and end of each *Phase*, and the corresponding age estimates for the start and end of each *Phase* were obtained from the modelled probability distributions of the *Boundaries*. The *Phases* were then arranged into a *Sequence* in which the lithostratigraphic (model A) or archaeological (model B) *Phases* are assembled in chronological order.

We cannot assume that sediments have accumulated continuously in South Chamber. Time gaps in the stratigraphy may arise due to erosional events, periods of little or no sediment deposition, or the absence of samples from these layers. Where major time gaps were apparent between two sediment layers or archaeological phases, we inserted an *Interval* and assigned each gap an assumed duration and uncertainty. To estimate the durations, we used the difference between the measured ages for each pair of layers as starting values for the model. The likelihood of there being a time interval was then estimated by the model, and the results incorporated into the modelled start and end ages of each *Phase*. The timing of the modelled gaps and their estimated durations are shown as *Intervals* between the grey bands in Supplementary Data 14 and 15.

We used a general *t*-type outlier model[70] to assess the likelihood of each age being consistent with the fitted model, and assigned a prior outlier probability of 5% to all ages in both models. The posterior outlier probabilities are estimated by the model and the ages weighted accordingly.

The Bayesian chronology for model A is presented in Fig. 5 (Supplementary Fig. 23 includes the individual age distributions for all six samples from layer 19) and for model B in Supplementary Fig. 24. The corresponding data are given in Supplementary Data 14 and 15, respectively, and the CQL codes used to generate these data are listed in Supplementary Codes 1 and 2, respectively. The modelled age ranges are shown at 68.2% and 95.4% probability, and the uncertainties on the start and end ages of each *Phase* are based on the total unshared component of error only. To compare the modelled ages with independent chronologies, it is necessary to include the total shared component of error, which is, on average, 4.00 ± 0.03% of the sample age for the data set of 60 samples; the median value is also 4.00%. This represents, on average, ~41% of the total (unshared plus shared) age uncertainty. To estimate the total uncertainties associated with the start and end ages of each *Phase* and *Interval*, we combined (in quadrature) the total unshared errors obtained from models A and B with the average total shared error of 4%; the latter age uncertainties should be used in comparisons with independent ages. The Bayesian model ages for the sediment layers, archaeological

phases and time gaps in the stratigraphic sequences in Main, East and South Chambers are shown on a common time scale in Supplementary Fig. 25 (total uncertainties at the 95% confidence interval).

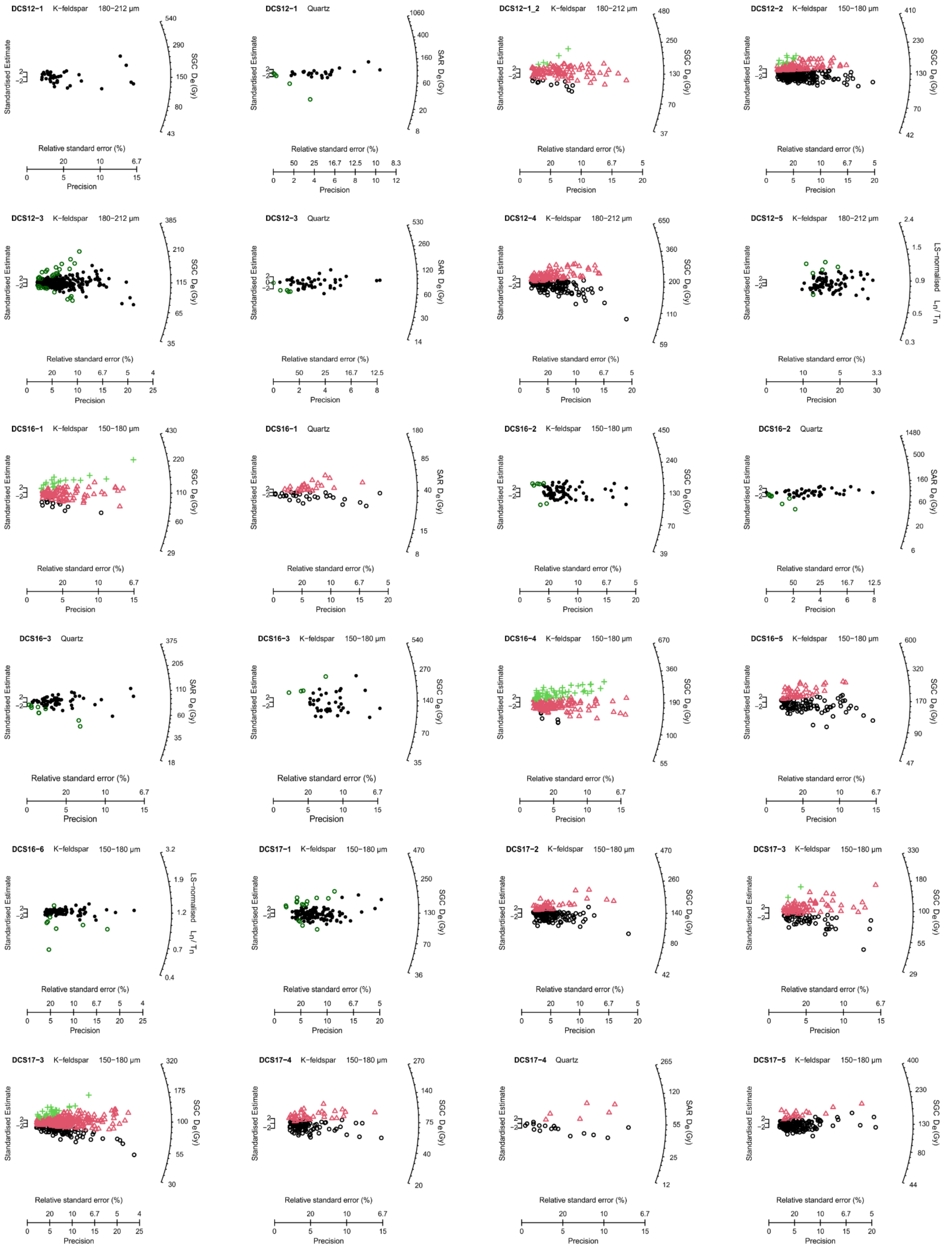

Supplementary Fig. 12 | Caption on page 40

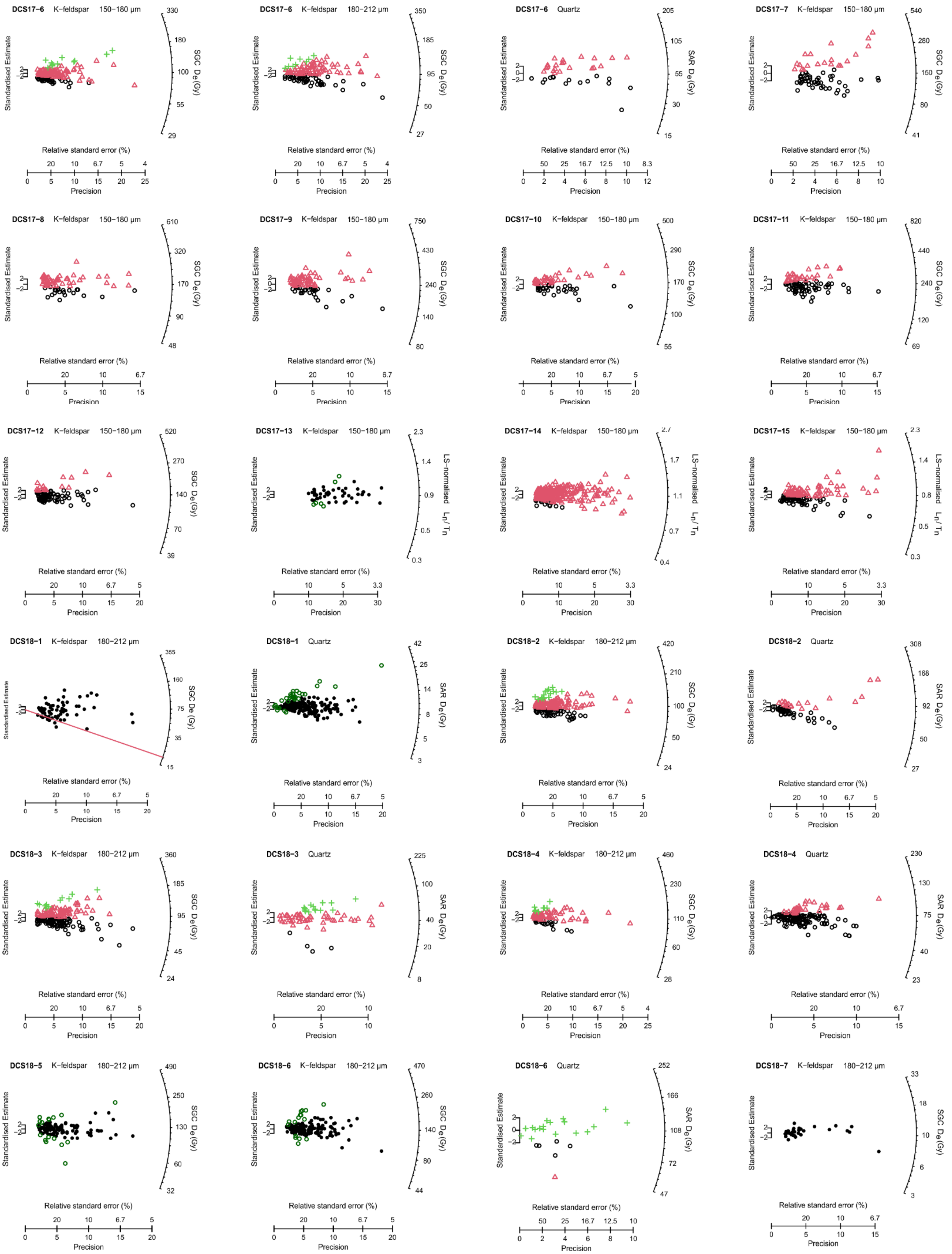

Supplementary Fig. 12 continued

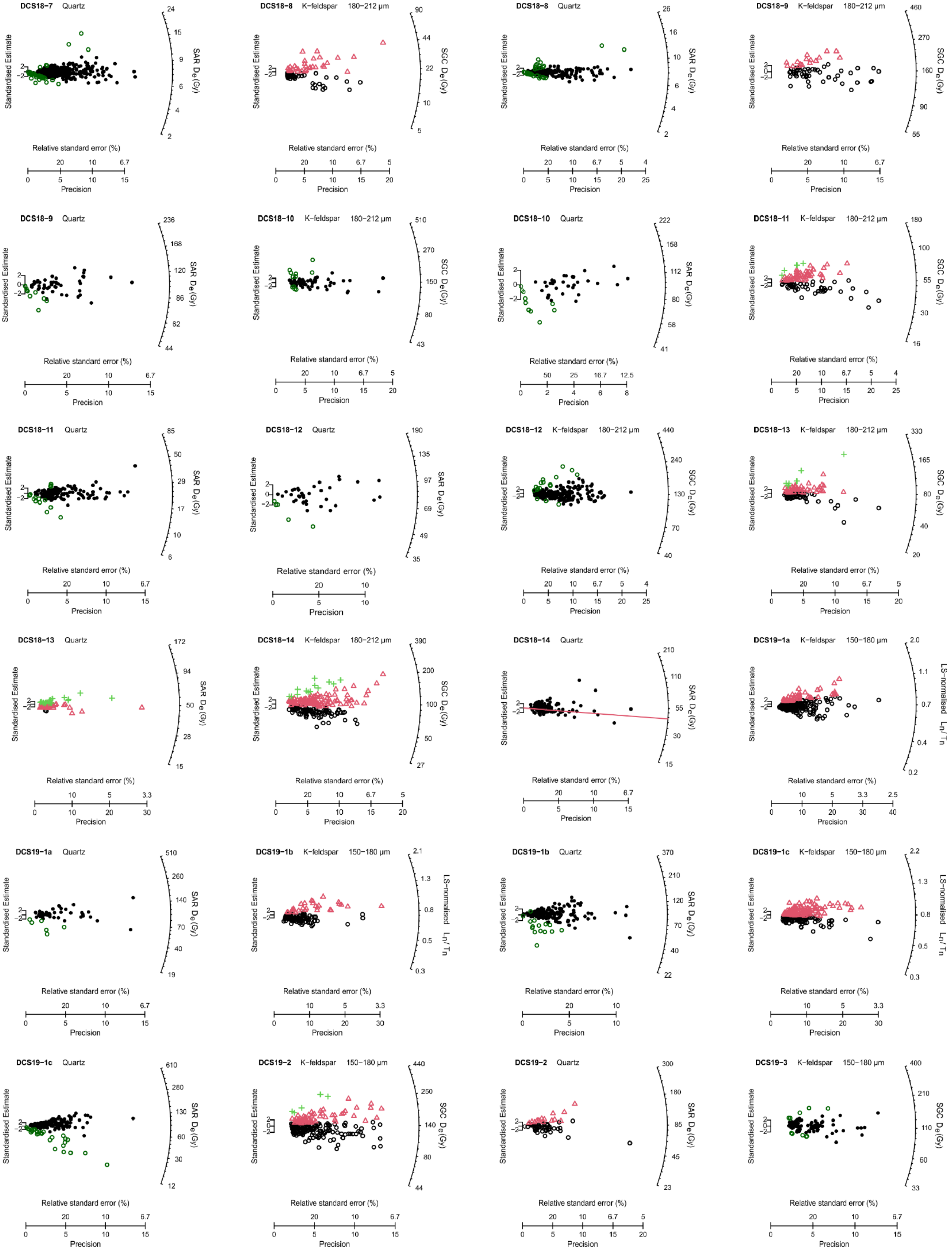

Supplementary Fig. 12 continued

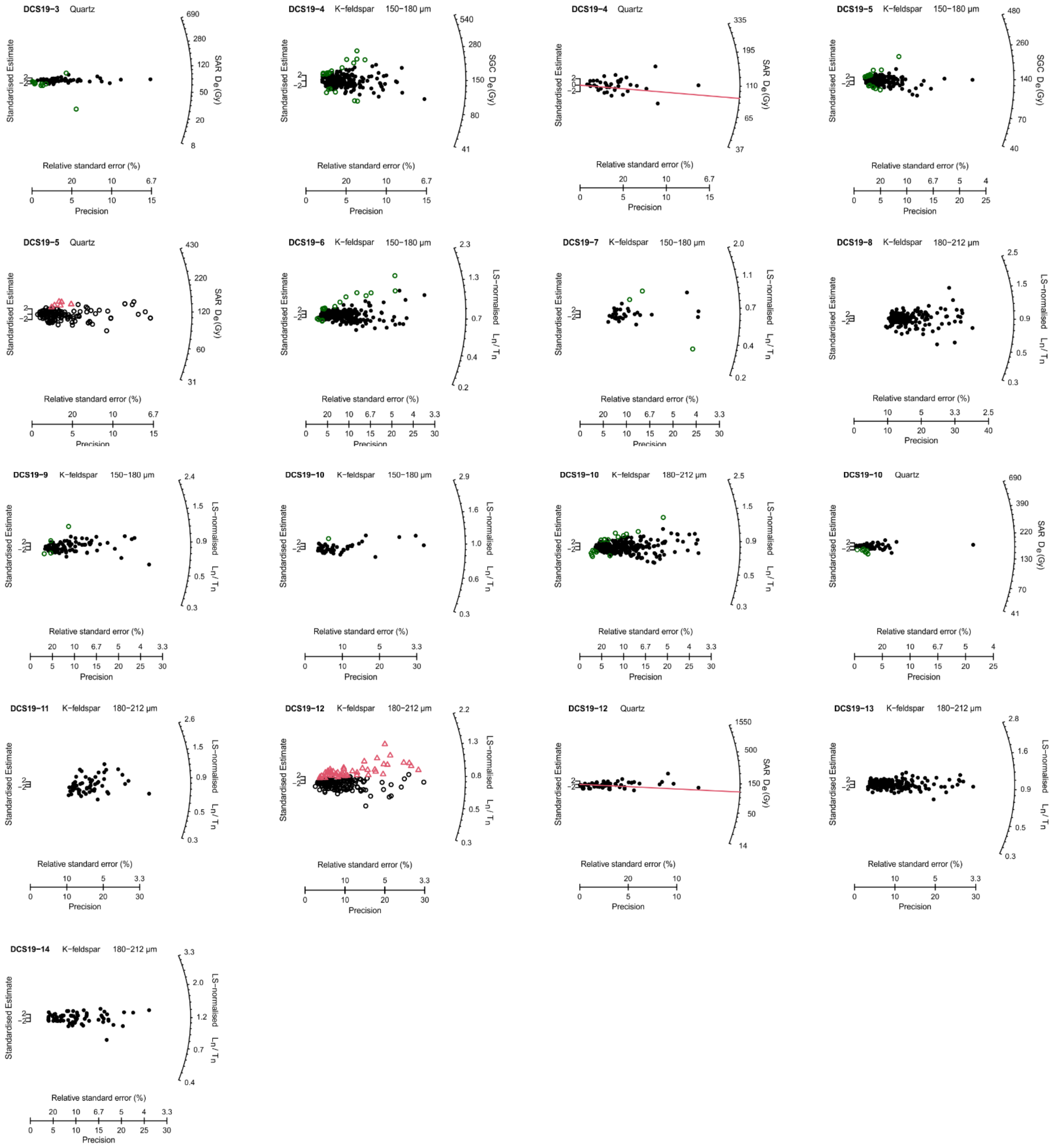

Supplementary Fig. 12 | Distributions of D_e values or least-squares normalized L_n/T_n values for individual grains of quartz and K-feldspar in sediment samples from South Chamber. The radial plots are arranged by year (starting with samples collected in 2012) and in numerical order within each year. Data are shown for grains with diameters of 180–212 µm (quartz and K-feldspar) or 150–180 µm (K-feldspar). K-feldspar distributions precede the quartz distributions for samples dated using both minerals. Symbols are as follows: values used for D_e estimation using the CAM or nMAD CAM (filled black circles); values identified as outliers using the nMAD (open green circles); values optimally fitted as 2 or 3 discrete components using the FMM (smallest to largest: open black circles, open red triangles, green crosses). For the multi-component distributions, only components containing more than 70% of grains were used for age determination (highlighted in bold in Supplementary Data 9). Red lines indicate the MAM estimates of D_e for samples DCS18-1 (K-feldspar), DCS18-14 (quartz), DCS19-4 (quartz) and DCS19-12 (quartz).

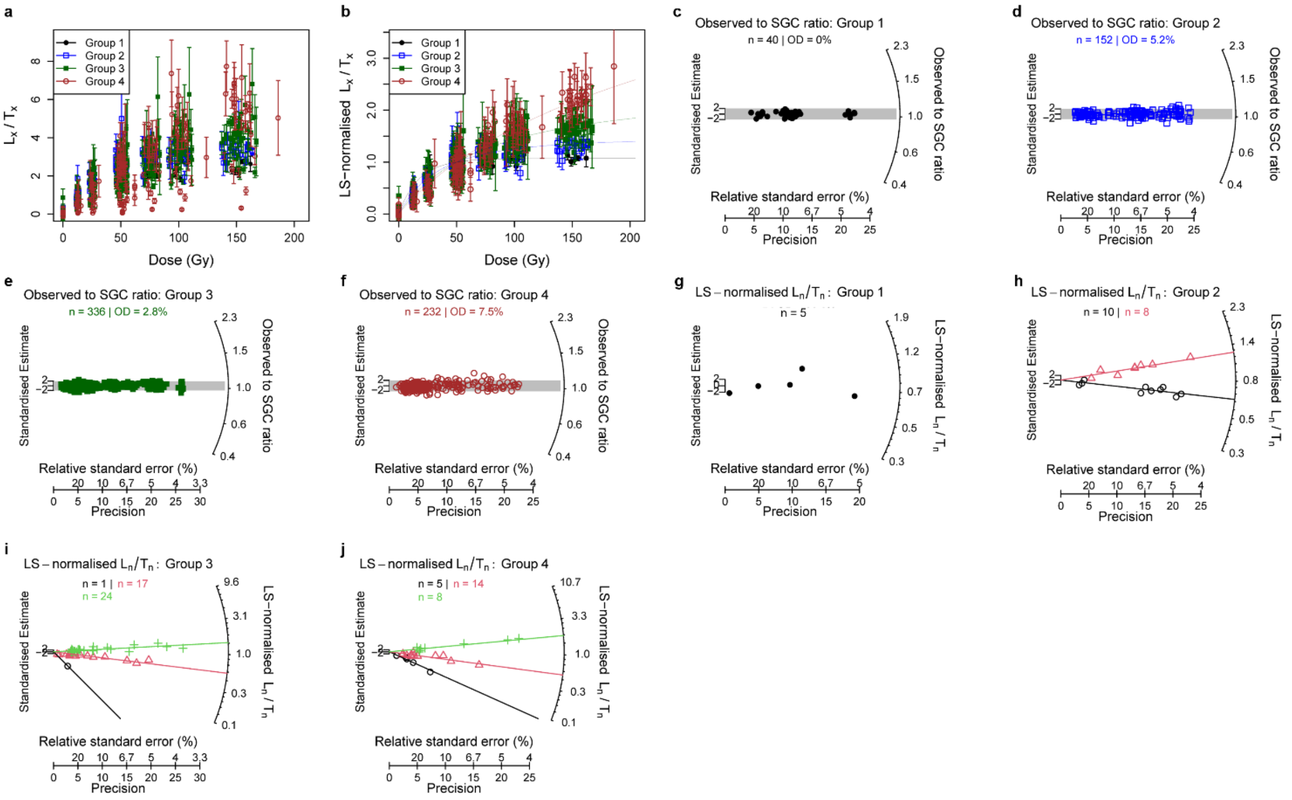

Supplementary Fig. 13 | L_x/T_x and L_n/T_n data for individual quartz grains from sample DCS18-5. a, L_x/T_x values for all accepted grains in each group. The number of groups was determined from fitting the FMM to the ratios of single-grain L_x/T_x values measured at two regenerative doses. **b**, Least-squares normalized L_x/T_x values for each group of grains. Standardized growth curves (SGCs) were fitted to these values using a general-order kinetics function and normalized to unity at a dose of 50 Gy. **c–f**, Plots of the ratios of measured (observed) L_x/T_x values to expected (SGC) L_x/T_x values. Shaded bands denote ± 2 standardized estimates. The total number of grains (n) and extent of overdispersion (OD) is shown for each group. **g–j**, Plots of the least-squares normalized L_n/T_n values for each group. Symbols as in Supplementary Fig. 12 for groups with multi-component distributions; lines indicate the FMM estimates of the mean value for each component. 'n' denotes the number of grains in each distribution (**g**) or FMM component (**h–j**).

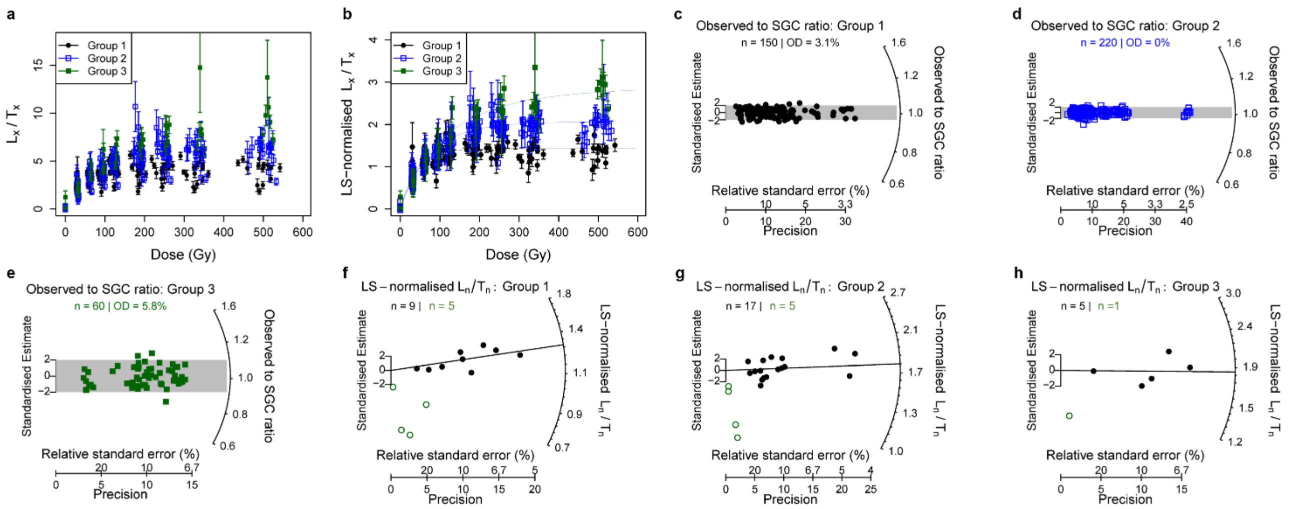

Supplementary Fig. 14 | L_x/T_x and L_n/T_n data for individual quartz grains from sample DCS19-6. **a**, L_x/T_x values for all accepted grains in each group. **b**, Least-squares normalized L_x/T_x values for each group of grains. **c–e**, Plots of the ratios of measured (observed) L_x/T_x values to expected (SGC) L_x/T_x values. **f–h**, Plots of the least-squares normalized L_n/T_n values for each group. Values used for D_e estimation using the CAM are shown as filled black circles. Lines indicate the CAM estimates of the mean value after removing outliers identified using the nMAD (open green circles).

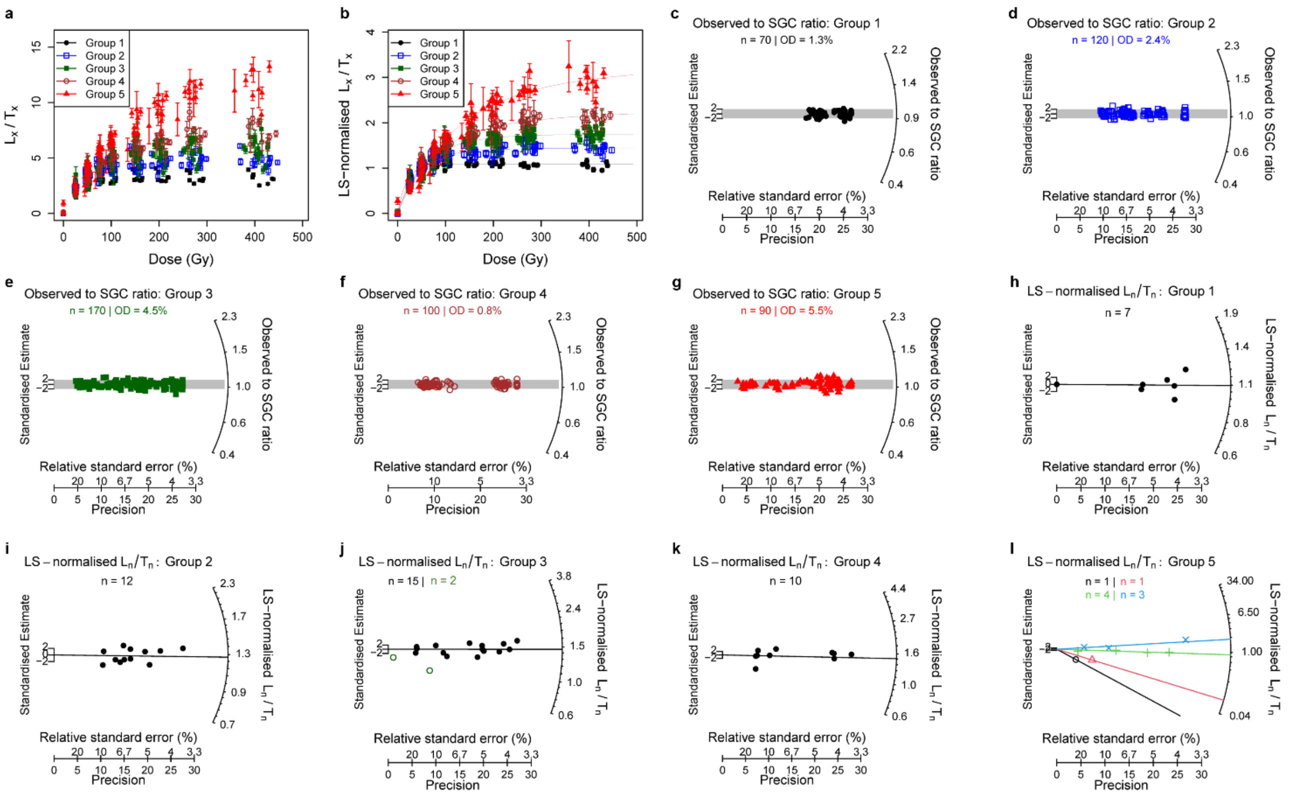

Supplementary Fig. 15 | L_x/T_x and L_n/T_n data for individual quartz grains from sample DCS19-7. a, L_x/T_x values for all accepted grains in each group. **b**, Least-squares normalized L_x/T_x values for each group of grains. **c–g**, Plots of the ratios of measured (observed) L_x/T_x values to expected (SGC) L_x/T_x values. **h–l**, Plots of the least-squares normalized L_n/T_n values for each group. Symbols as in Supplementary Figs. 13 and 14.

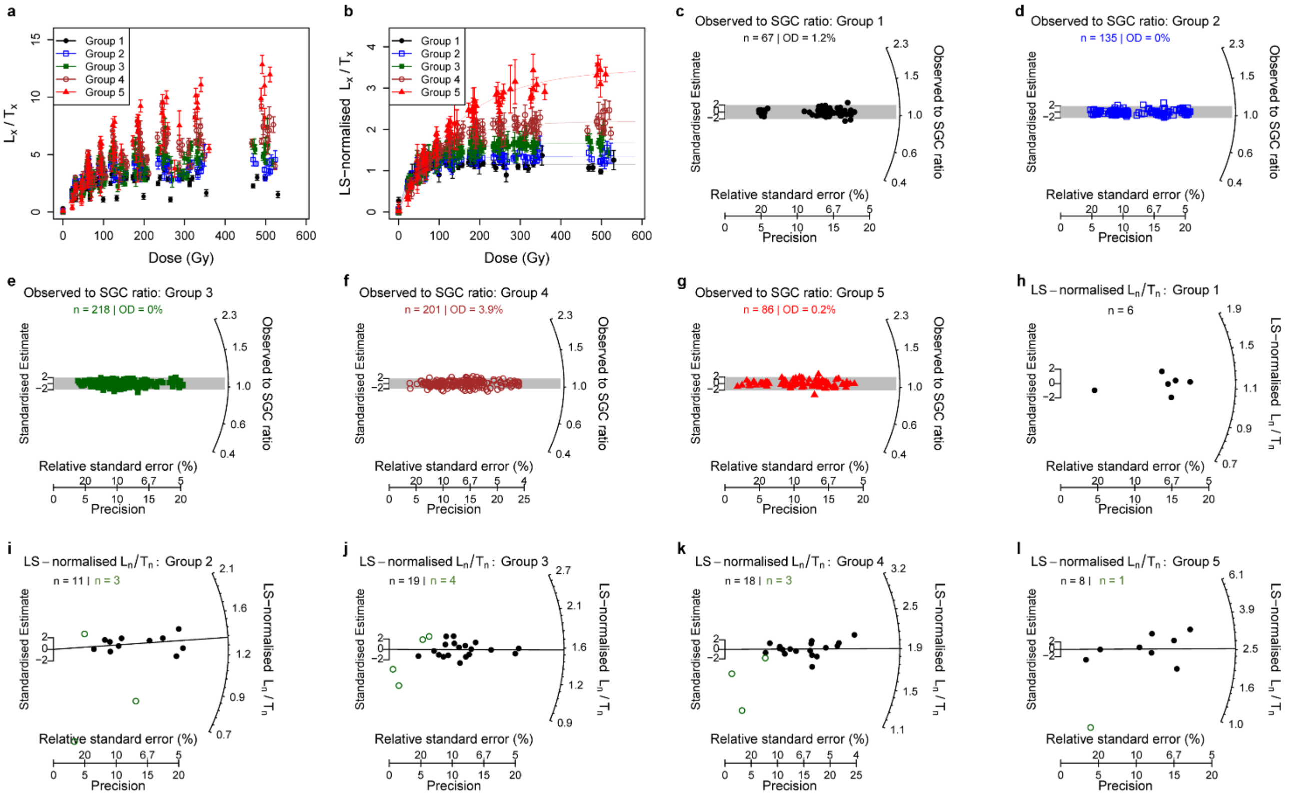

Supplementary Fig. 16 | L_x/T_x and L_n/T_n data for individual quartz grains from sample DCS19-8. a, L_x/T_x values for all accepted grains in each group. **b**, Least-squares normalized L_x/T_x values for each group of grains. **c–g**, Plots of the ratios of measured (observed) L_x/T_x values to expected (SGC) L_x/T_x values. **h–l**, Plots of the least-squares normalized L_n/T_n values for each group. Symbols as in Supplementary Fig. 14.

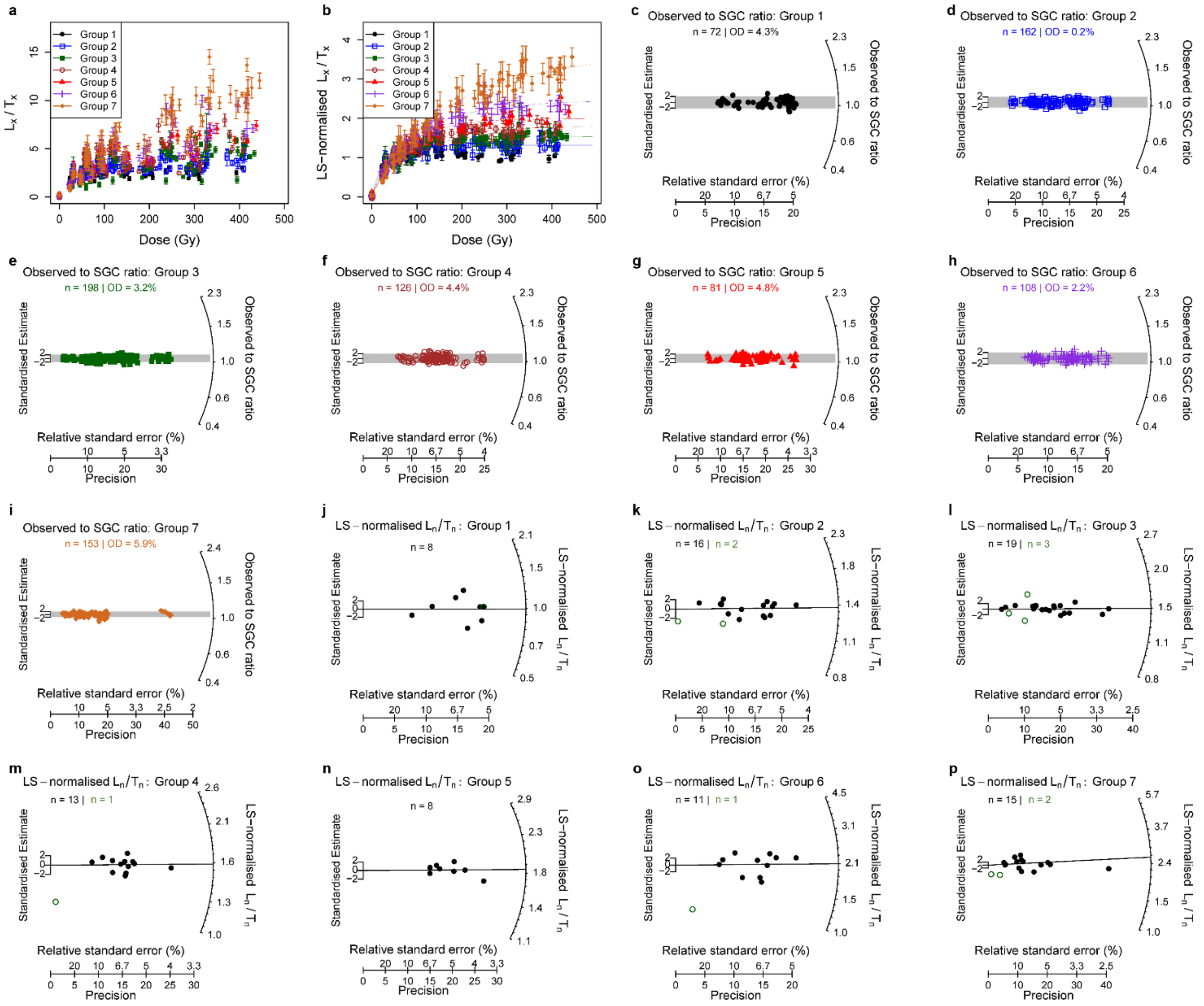

Supplementary Fig. 17 | L_x/T_x and L_n/T_n data for individual quartz grains from sample DCS19-9. a, L_x/T_x values for all accepted grains in each group. **b**, Least-squares normalized L_x/T_x values for each group of grains. **c–i**, Plots of the ratios of measured (observed) L_x/T_x values to expected (SGC) L_x/T_x values. **j–p**, Plots of the least-squares normalized L_n/T_n values for each group. Symbols as in Supplementary Fig. 14.

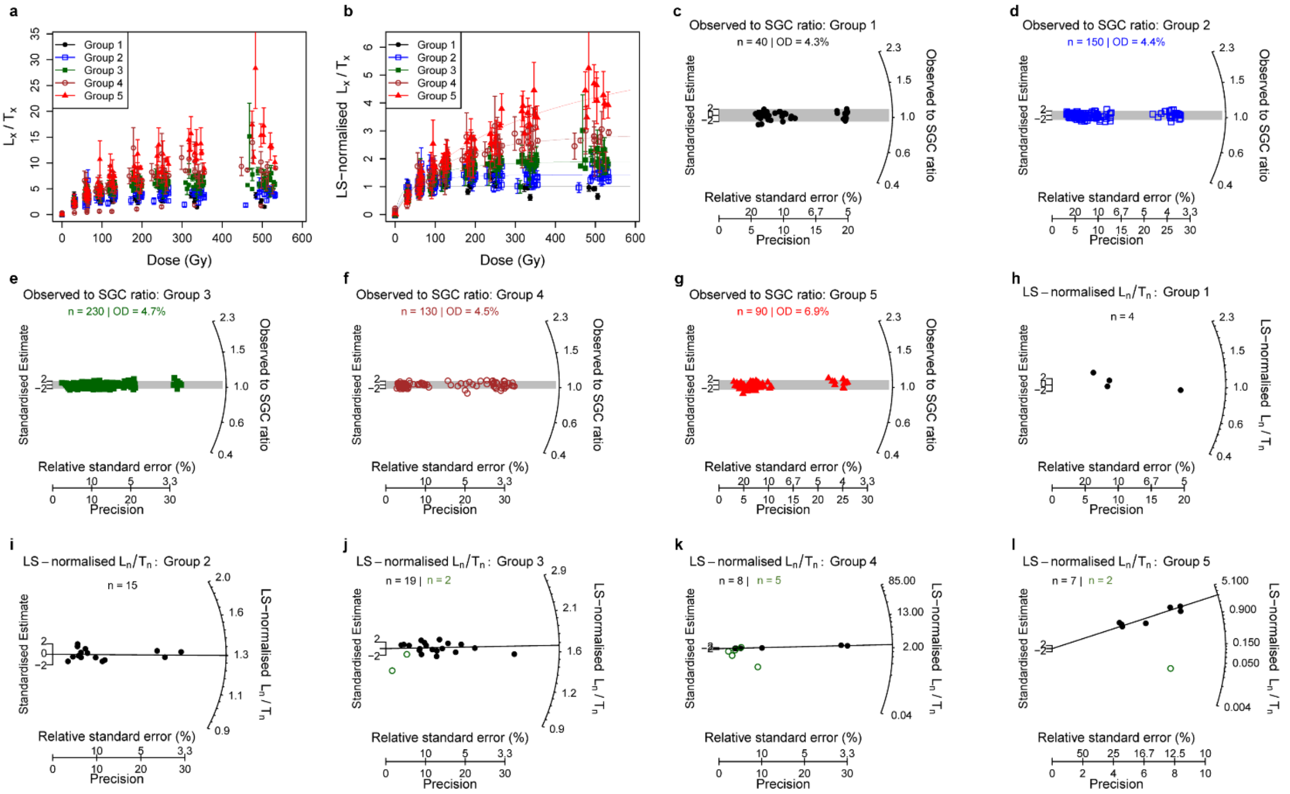

Supplementary Fig. 18 | L_x/T_x and L_n/T_n data for individual quartz grains from sample DCS19-11. a, L_x/T_x values for all accepted grains in each group. **b**, Least-squares normalized L_x/T_x values for each group of grains. **c–g**, Plots of the ratios of measured (observed) L_x/T_x values to expected (SGC) L_x/T_x values. **h–l**, Plots of the least-squares normalized L_n/T_n values for each group. Symbols as in Supplementary Fig. 14.

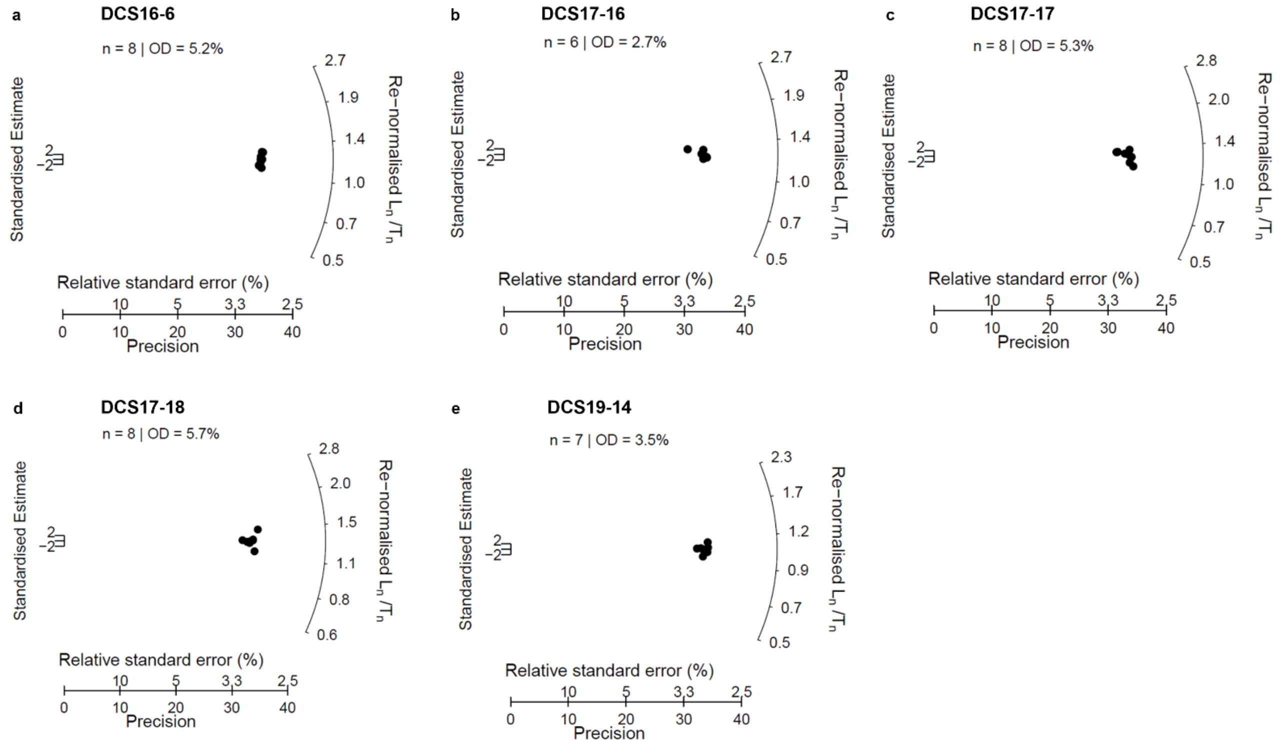

Supplementary Fig. 19 | Distributions of re-normalized L_n/T_n values for K-feldspar samples measured using the multiple-aliquot regenerative-dose (MAR) procedure. The sample codes are shown at the top of each panel (**a–e**), together with the number of aliquots and extent of overdispersion (OD) of the re-normalized L_n/T_n values, which were obtained from the pIRIR signals measured at a stimulation temperature of 275°C. The weighted mean was calculated for each distribution using the CAM and projected onto the 275°C MAR SGC to estimate the corresponding sample D_e. All five samples are from layer 19.

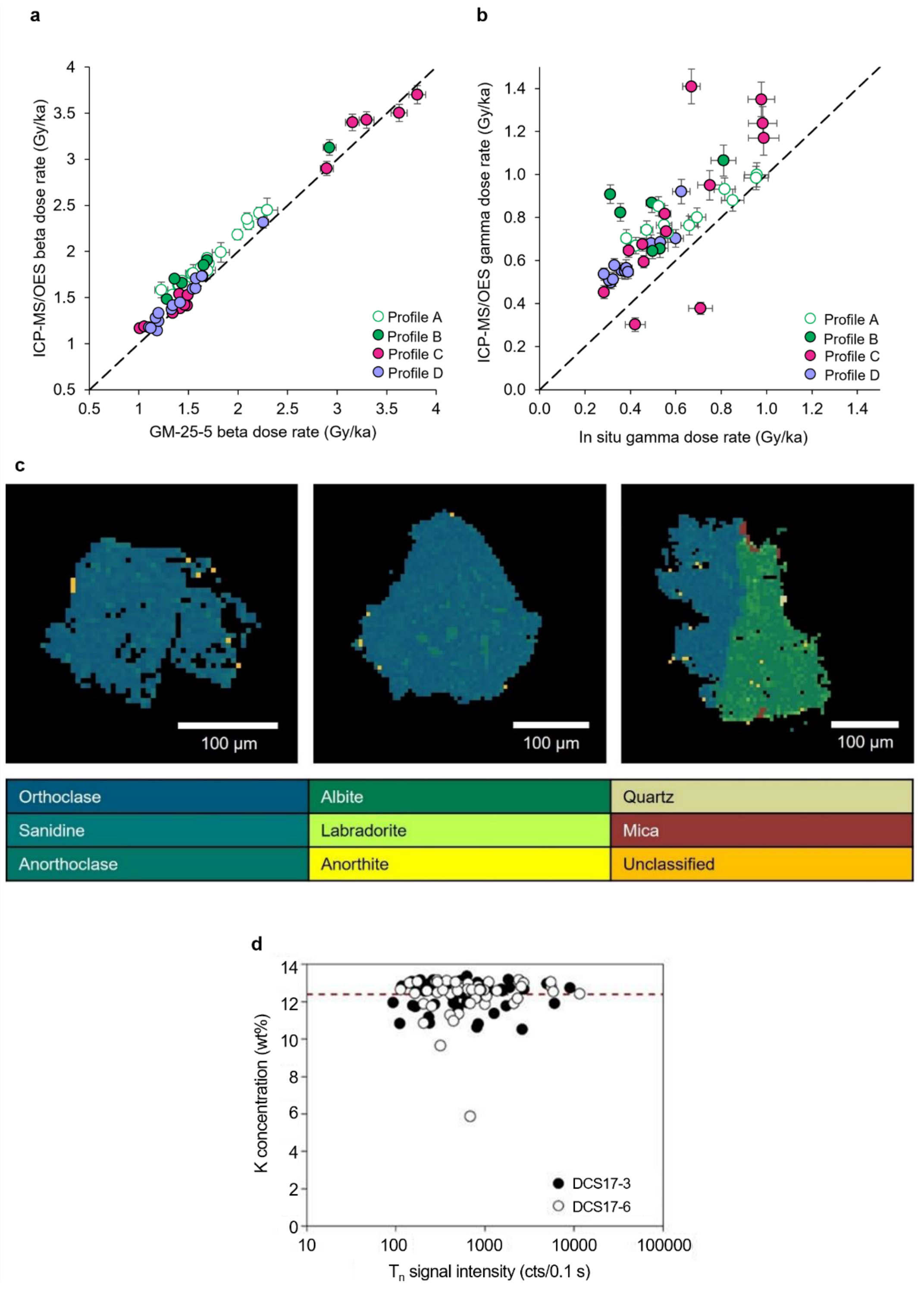

Supplementary Fig. 20 | Dose rate comparisons for sediment samples from South Chamber and internal dose rates of individual feldspar grains. **a**, Comparison of dry and unattenuated external beta dose rates determined from GM-25-5 beta counting and ICP-MS/OES measurements (n = 52). The dose rate uncertainties are at 2σ and the dashed line indicates the 1:1 ratio. **b**, Comparison of gamma dose rates determined from in situ gamma spectrometry and ICP-MS/OES measurements (n = 45). The dose rate uncertainties are at 1σ and the dashed line indicates the 1:1 ratio. **c**, QEM-EDS maps of three individual feldspar grains accepted for D_e determination from samples DCS17-3 (left-hand panel) and DCS17-6 (middle and right-hand panels). **d**, Whole-of-grain average K concentrations of individual feldspar grains from samples DCS17-3 (filled circles, n = 62) and DCS17-6 (open circles, n = 54), plotted as a function of T_n signal intensity. The dashed line indicates the arithmetic mean K concentration (12.4 wt%).

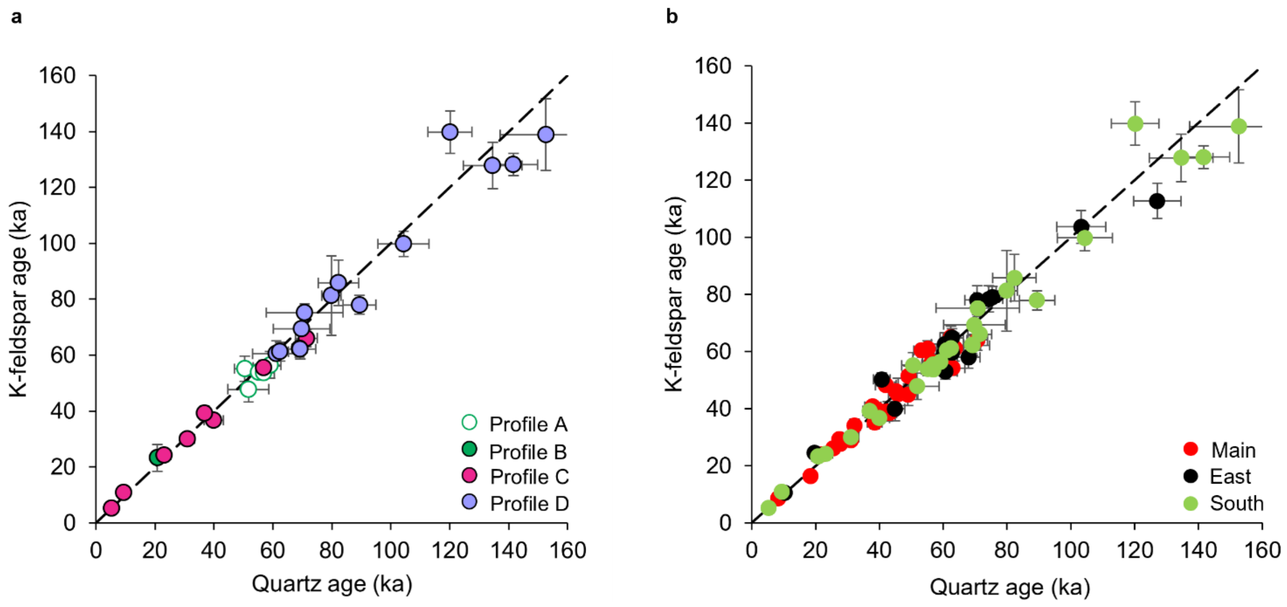

Supplementary Fig. 21 | Comparisons of single-grain OSL and pIRIR ages for sediment samples from South Chamber and all three chambers. a, Weighted mean ages for 28 samples from South Chamber measured using both quartz and K-feldspar (Supplementary Data 12). **b**, Weighted mean ages for 71 samples measured using both quartz and K-feldspar from Main, East and South Chambers (n = 28, 15 and 28 samples, respectively); data for Main and East Chambers from [1]. In each plot, the age uncertainties are at 1σ and the dashed line indicates the 1:1 ratio.

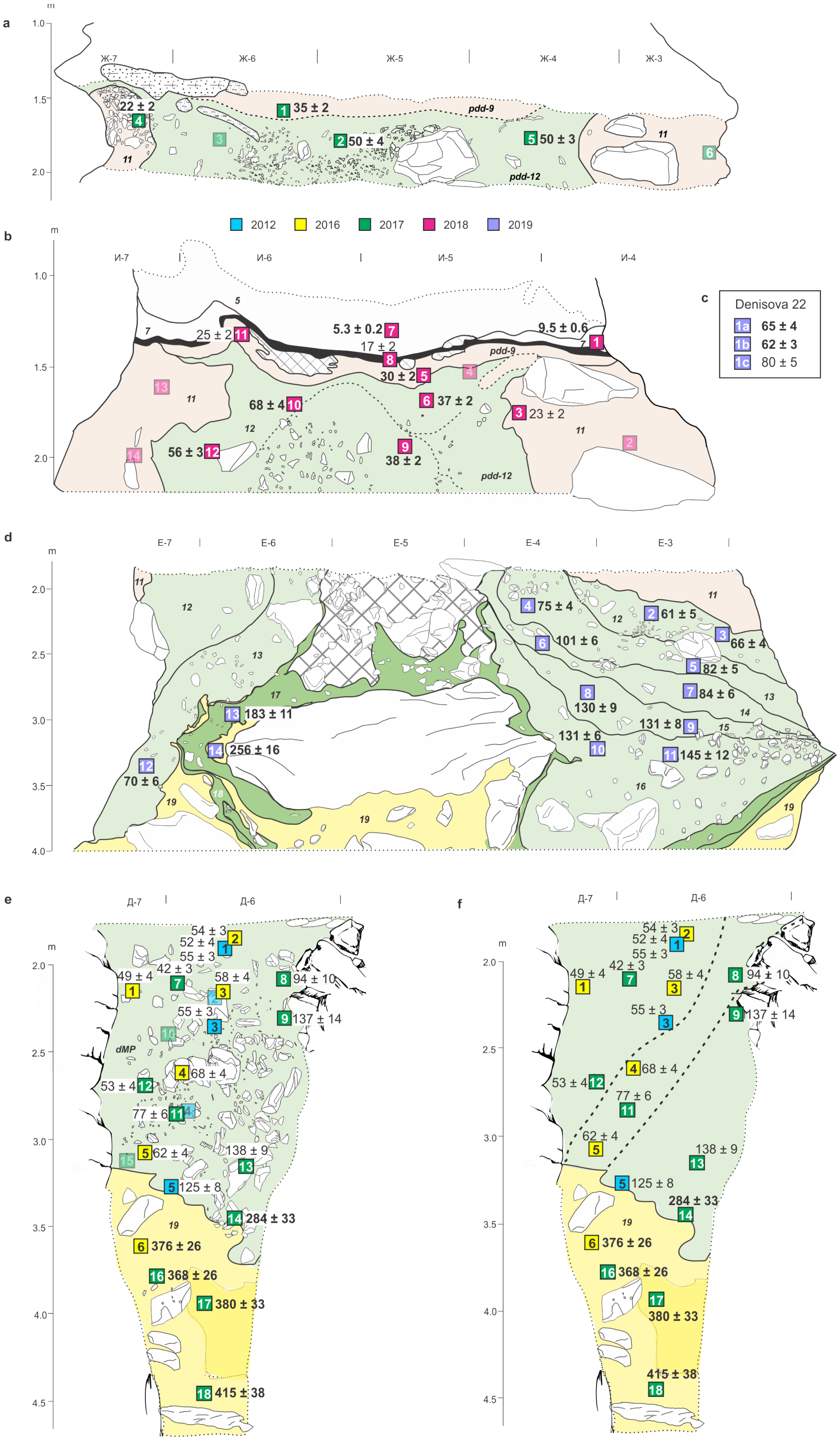

a
b
c
d
e
f
m
1.0
1.5
2.0
2.5
3.0
3.5
4.0
4.5
Ж-7
Ж-6
Ж-5
Ж-4
Ж-3
22 ± 2
35 ± 2
50 ± 4
50 ± 3
pdd-9
pdd-12
11
2012
2016
2017
2018
2019
И-7
И-6
И-5
И-4
25 ± 2
5.3 ± 0.2
17 ± 2
9.5 ± 0.6
30 ± 2
68 ± 4
37 ± 2
23 ± 2
56 ± 3
38 ± 2
5
7
11
12
Denisova 22
1a 65 ± 4
1b 62 ± 3
1c 80 ± 5
Е-7
Е-6
Е-5
Е-4
Е-3
75 ± 4
61 ± 5
101 ± 6
66 ± 4
82 ± 5
130 ± 9
84 ± 6
183 ± 11
131 ± 8
131 ± 6
256 ± 16
145 ± 12
70 ± 6
13
14
15
16
17
18
19
Д-7
Д-6
54 ± 3
52 ± 4
55 ± 3
49 ± 4
42 ± 3
58 ± 4
94 ± 10
55 ± 3
137 ± 14
dMP
68 ± 4
53 ± 4
77 ± 6
62 ± 4
138 ± 9
125 ± 8
284 ± 33
376 ± 26
368 ± 26
380 ± 33
415 ± 38

Supplementary Fig. 22 │ Optical ages of sediment samples included in Bayesian age models. Age estimates and standard errors (in ka) for samples collected from the southeast faces of Profile B in 2016 (**a**), Profile C in 2018 (**b**), Profile D (including three samples beneath Denisova 22) in 2019 (**c**, **d**) and Profile A in 2012, 2016 and 2017 (**e**, **f**). Layer colours as in Fig. 6. D_e values and ages of all samples are listed in Supplementary Data 9, together with the proportion of grains in each component for samples with multi-component distributions. Ages in bold font (n = 31) are considered the most reliable estimates of time of deposition of the relevant layers and are included in Bayesian age model A (Fig. 5; Supplementary Figs. 23 and 25d). These ages correspond to samples with single-component D_e distributions, or samples with multi-component distributions in which the main component contains at least 70% of grains. An additional criterion for samples with multi-component distributions obtained from both quartz and K-feldspar grains is that the OSL and pIRIR ages for the main component are statistically consistent; we calculated the weighted mean age for these samples. The ages shown in regular font (n = 19) are included as additional age estimates for the archaeological phases in Bayesian age model B (Supplementary Figs. 24 and 25e). Samples indicated by faint squares with no associated ages (n = 10) have multi-component D_e distributions with fewer than 70% of grains in the main component; or if both quartz and K-feldspar grains were measured, the OSL and pIRIR ages for the main component are statistically inconsistent. These samples are not included in either Bayesian model and are not displayed in **f**. The dashed lines in **f** demarcate the three broad age clusters that correspond to ages in Profiles B–D of layers 16 and 15 (lower right), 14 to 12 (middle), and pdd-9/12 and 11 (upper left).

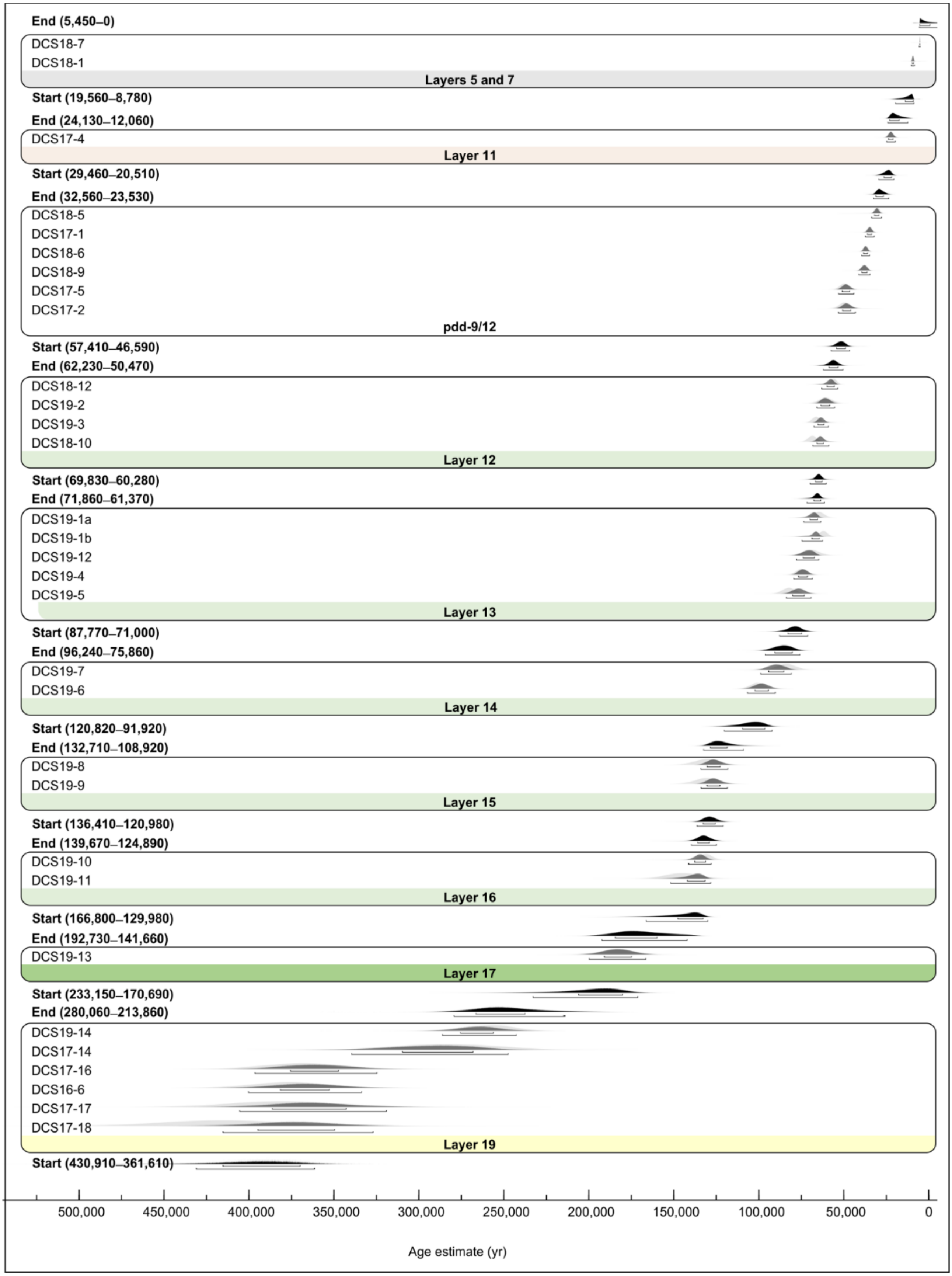

Supplementary Fig. 23 | Bayesian age model A for South Chamber, including age distributions of samples from layer 19. This plot shows the individual age distributions of the six samples from layer 19 in addition to the age distributions displayed in Fig. 5. Symbols and notation as in Fig. 5.

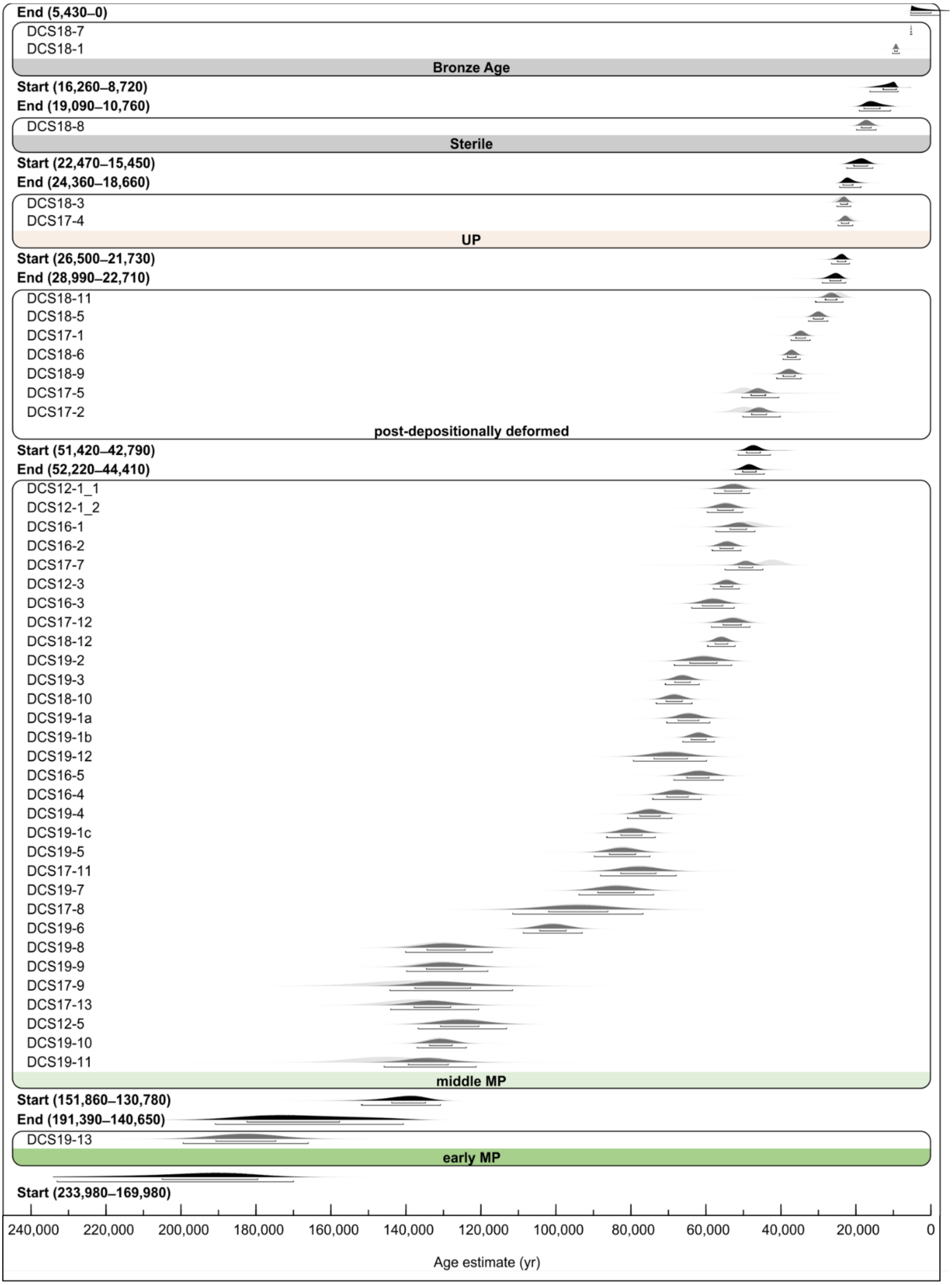

Supplementary Fig. 24 | Bayesian age model B for South Chamber. The ages included in this model (n = 50) include those for dMP samples with D_e distributions dominated by a single population of grains or by a component that contains more than 70% of the grains. We used the sequence of archaeological phases as prior information to further constrain the start and end ages of the Middle and Upper Palaeolithic in South Chamber. Symbols and notation as in Fig. 5.

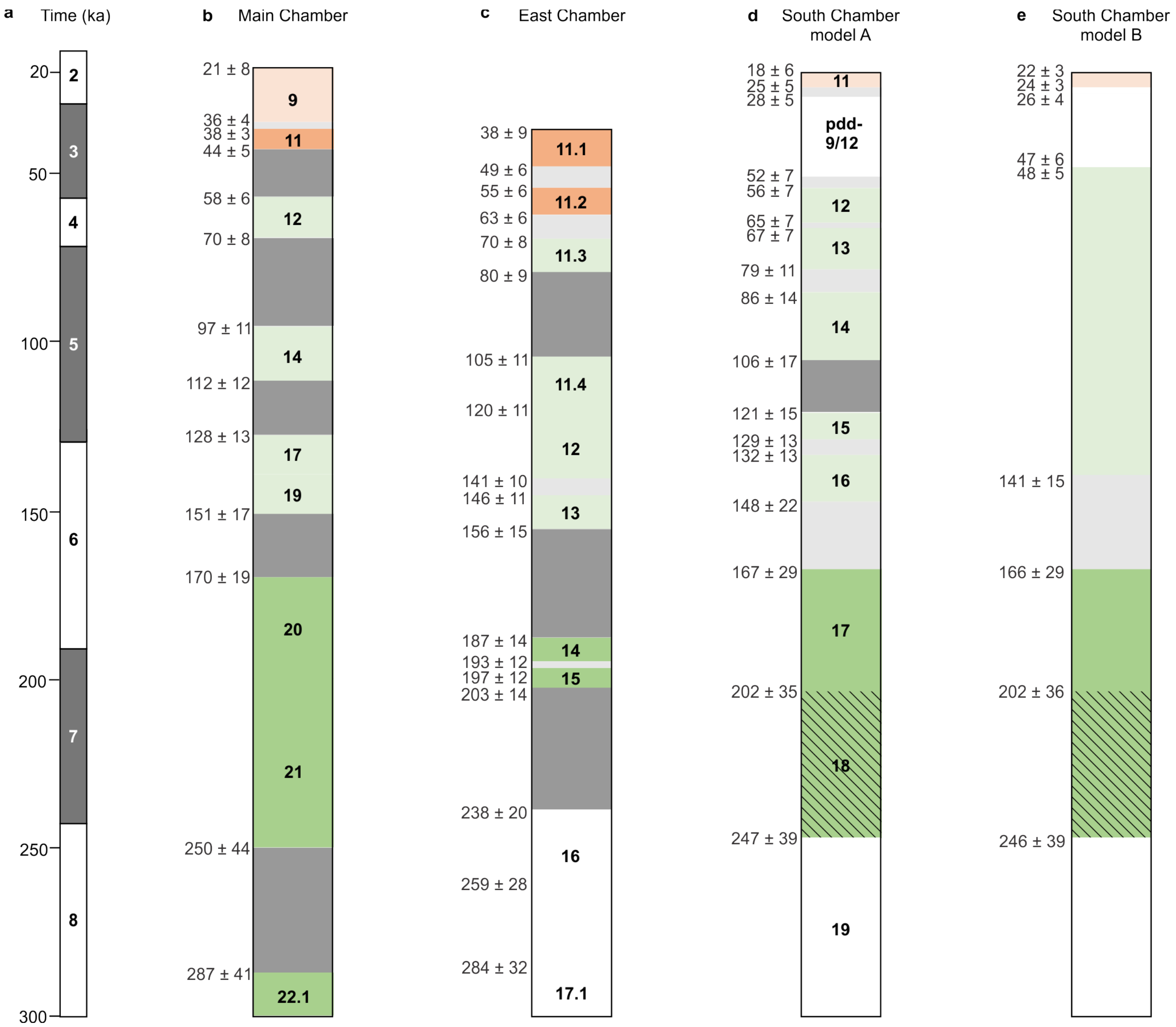

Supplementary Fig. 25 | Bayesian model ages for sediment layers and archaeological phases at Denisova Cave. **a**, Common time scale for Main, East and South Chambers, with boundary ages of marine isotope stages from [77]. **b**, **c**, Stratigraphic sequences in Main and East Chambers, respectively, with modelled start and end ages for layers and time gaps from [1] (total uncertainties at the 95% confidence interval, including all known and estimated sources of random and systematic error). Layer colours denote archaeological phases: early Middle Palaeolithic (dark green), middle Middle Palaeolithic (light green), Initial Upper Palaeolithic (dark orange) and Upper Palaeolithic (light orange). **d**, **e**, Stratigraphic sequence in South Chamber, with start and end ages based on Bayesian model A (**d**) or model B (**e**). Layer 18 was not sampled for optical dating. Model A (n = 31 samples) provides age estimates for each layer or combination of layers (Fig. 5; Supplementary Fig. 23), whereas model B (n = 50 samples) provides age estimates for the early Middle Palaeolithic, middle Middle Palaeolithic and Upper Palaeolithic archaeological phases (Supplementary Fig. 24).

SUPPLEMENTARY SECTION 3 | Sediment DNA of ancient hominins and fauna in South Chamber (Elena I. Zavala, Stéphane Peyrégne, Viviane Slon, Svante Pääbo, Janet Kelso and Matthias Meyer)

Sediment samples were collected for ancient DNA analysis from Profiles A and B in 2017[2] and from Profiles C and D in 2019. Supplementary Data 16 contains summaries of the samples collected in 2019 that underwent genetic analysis, and the mammalian mtDNA and hominin mtDNA data so obtained are summarized in Supplementary Data 17 and 18, respectively. Individual DNA sample locations are shown in Supplementary Fig. 26 for Profiles C and D and in Supplementary Fig. 3 of [2] for Profiles A and B. The locations of individual optical dating samples collected from Profiles C and D (in 2018 and 2019, respectively) are also shown in Supplementary Fig. 26.

3.1 Analysis of ancient mammalian mtDNA in sediments

Almost all of the sediment samples collected from Profiles C and D were tested for the preservation of ancient mammalian mtDNA by hybridization capture with mammalian mtDNA probes. The two samples not tested (DCS19-161 and -164) produced low efficiency estimates in library preparation based on the quantification of a spike-in oligonucleotide[71] (Supplementary Data 16), presumably due to the presence of inhibitory substances in the DNA extracts. Both of these samples were collected from the heavily phosphatized layers (pdd-9 and -12, respectively), which have previously shown poorer ancient DNA preservation than samples from other layers[2].

For the 228 sediment samples enriched for mammalian mtDNA, 155 (68%) yielded evidence for the presence of DNA from at least one mammalian family. This success rate is lower than in previous work on samples from Main and East Chambers[2], likely due to a larger proportion of the samples (100 of 230, 43%) originating from pdd-9 and -12 and the guano-rich Holocene deposits[3,4]. The sediments adhering to the deer tooth pendant[72] and the samples collected near the mammoth ivory figurine[23] and from directly beneath Denisova 22 all contained ancient mammalian mtDNA.

To evaluate changes in the composition of mammalian DNA through the South Chamber stratigraphy, we combined the data set for the 2019 samples with the previously published data set for the sediment samples collected from Profiles A and B[2], resulting in a total of 432 sediment samples. In addition, five sediment samples (three from beneath Denisova 22, one from near the ivory figurine, and one from the tooth pendant) were enriched for mammalian mtDNA; the resulting data are presented in Supplementary Fig. 27.

The relative abundance of mtDNA fragments from different mammalian families was calculated based on the average proportion of mtDNA fragments assigned to each family per sample per layer. These abundances were then compared to the newly compiled palaeontological data sets for large mammals (Supplementary Section 1.4) and small vertebrates (Supplementary Section 1.5). Layer 19, the oldest layer, contained mostly ursid fragments, whereas the genetic and skeletal data sets are dominated by bovid fragments in the younger layers. Interestingly, while the proportions of hyaena mtDNA fragments recovered from individual layers in Main and East Chambers are typically higher than those of hyaena skeletal remains[2], the relative abundances are more similar in South Chamber (Fig. 9b). Although no hyaena skeletal remains were found in layer 19, we recovered hyaena mtDNA from the sediments in this layer. These differences may reflect different patterns of use of the chambers and highlight the need for a deeper understanding of sediment DNA taphonomy to interpret the genetic and skeletal abundance data.

We also evaluated the presence of different species of bear and types of hyaena in layers 19 and 17–11 using previously defined mtDNA diagnostic positions[2]. This analysis resulted in 76 samples identified as containing ancient bear mtDNA (40–11,694 fragments) and 94 samples with ancient hyaena mtDNA (111–15,055 fragments). Cave and brown bear mtDNA was detected in layers 19 and 17–15), but only brown bear mtDNA was detected in layers 12 and 11 (Supplementary Figs. 28a–c and 29). Hyaena mtDNA from haplogroup D was detected in layer 17, while haplogroup A was

detected in layers 16–11 (Supplementary Figs. 28d–f and 29). These patterns are consistent with the records of bears and hyaenas in Main and East Chambers[2] (Fig. 10).

3.2 Identification of ancient hominin mtDNA in sediments

All of the sediment samples in which ancient mtDNA was detected in the mammalian mtDNA capture (and, in addition, sample DCS19-1) were also enriched specifically for hominin mtDNA. Of these 156 samples, 36 (23%) yielded evidence of ancient hominin mtDNA (43–1474 unique hominin mtDNA sequences per sample, or 15–482 sequences when limiting the analysis to putatively deaminated fragments based on C-to-T substitutions on the three terminal bases). Ancient hominin mtDNA was also identified in sediments removed from the previously published deer tooth pendant[72] and in the samples collected near the mammoth ivory figurine and beneath Denisova 22 (Supplementary Data 18).

Previously defined diagnostic positions between hominin mtDNA lineages[2] were used to evaluate the support for different hominin groups. This permitted the detection of Neanderthal, Denisovan or early modern human mtDNA in 31 of the 36 samples. Sample DCS19-19 in layer 13 contained support for both Neanderthal and Denisovan mtDNA (Fig. 6d). The five samples for which we could not determine a mtDNA lineage contained 43–166 mtDNA fragments (16–38 putatively deaminated fragments), indicating a lack of power for lineage assignment.

Lastly, we attempted to further differentiate specific Neanderthal and Denisovan mtDNA types using kallisto[73] to classify mtDNA fragments as previously described[2,74]. For this evaluation, we examined samples with at least 250 endogenous hominin reads after accounting for estimated modern human contamination based on the support for modern human mtDNA among diagnostic positions. This allowed us to identify 12 samples with Denisova 3- or 4-like mtDNA in layers 14 or 13, in addition to sample DCS17-127 from the dMP deposits in Profile A[2], and one sample with Denisova 2- or 8-like mtDNA in layer 17 (Fig. 6c,d; Fig. 7; Supplementary Fig. 30).

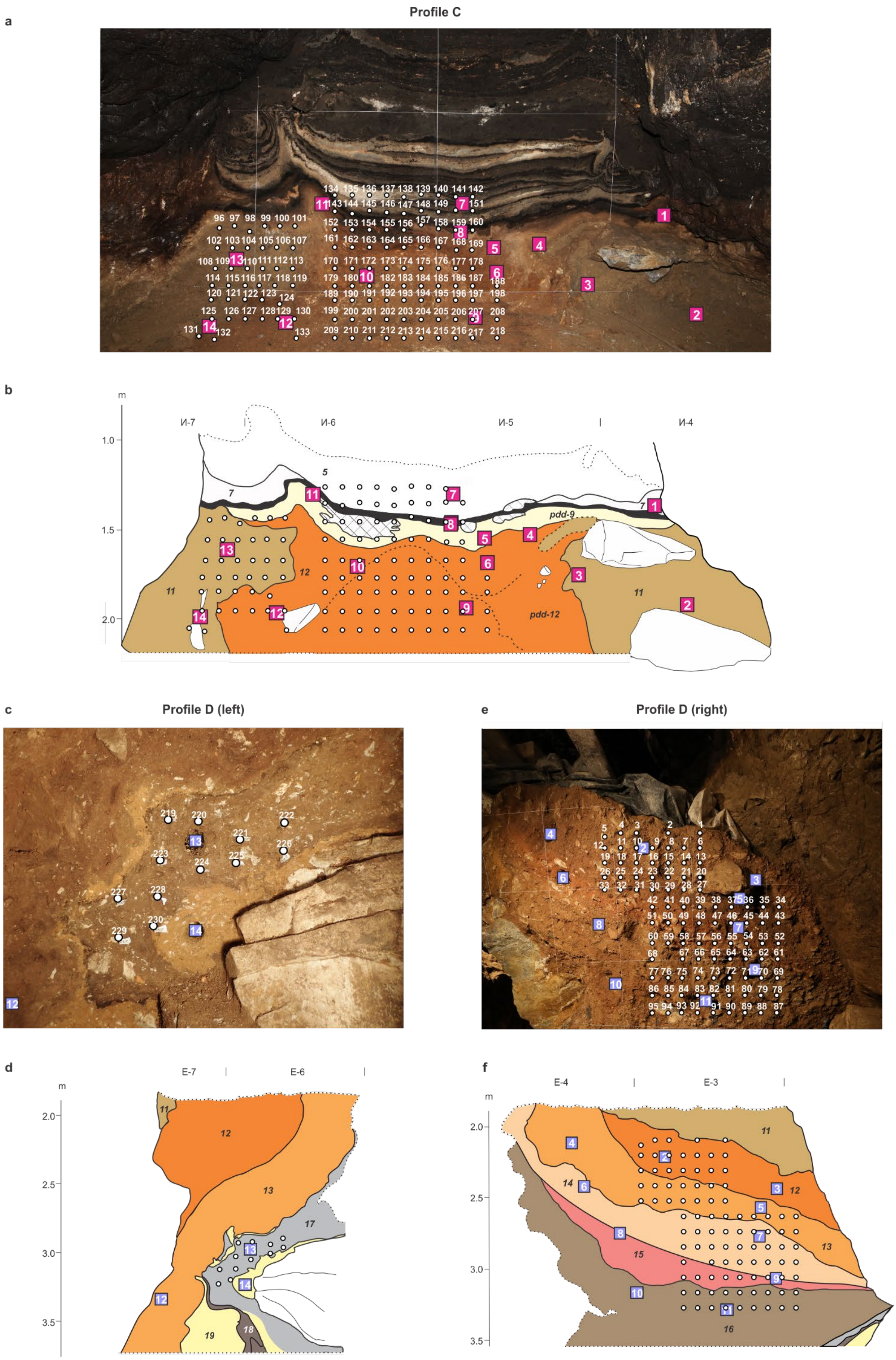
a
Profile C
b
m
И-7
И-6
И-5
И-4
pdd-9
pdd-12
c
Profile D (left)
e
Profile D (right)
d
E-7
E-6
f
E-4
E-3

Supplementary Fig. 26 │ Sediment samples collected for ancient DNA analysis and optical dating from Profiles C and D. Southeast face of Profile C after excavation in 2018 (**a**, **b**) and southeast faces of Profile D (**c**, **d**, left side; **e**, **f**, right side) after excavation in 2019. Colours and numbers of individual layers and optical dating samples as in Fig. 2. White circles and numbers denote individual DNA samples.

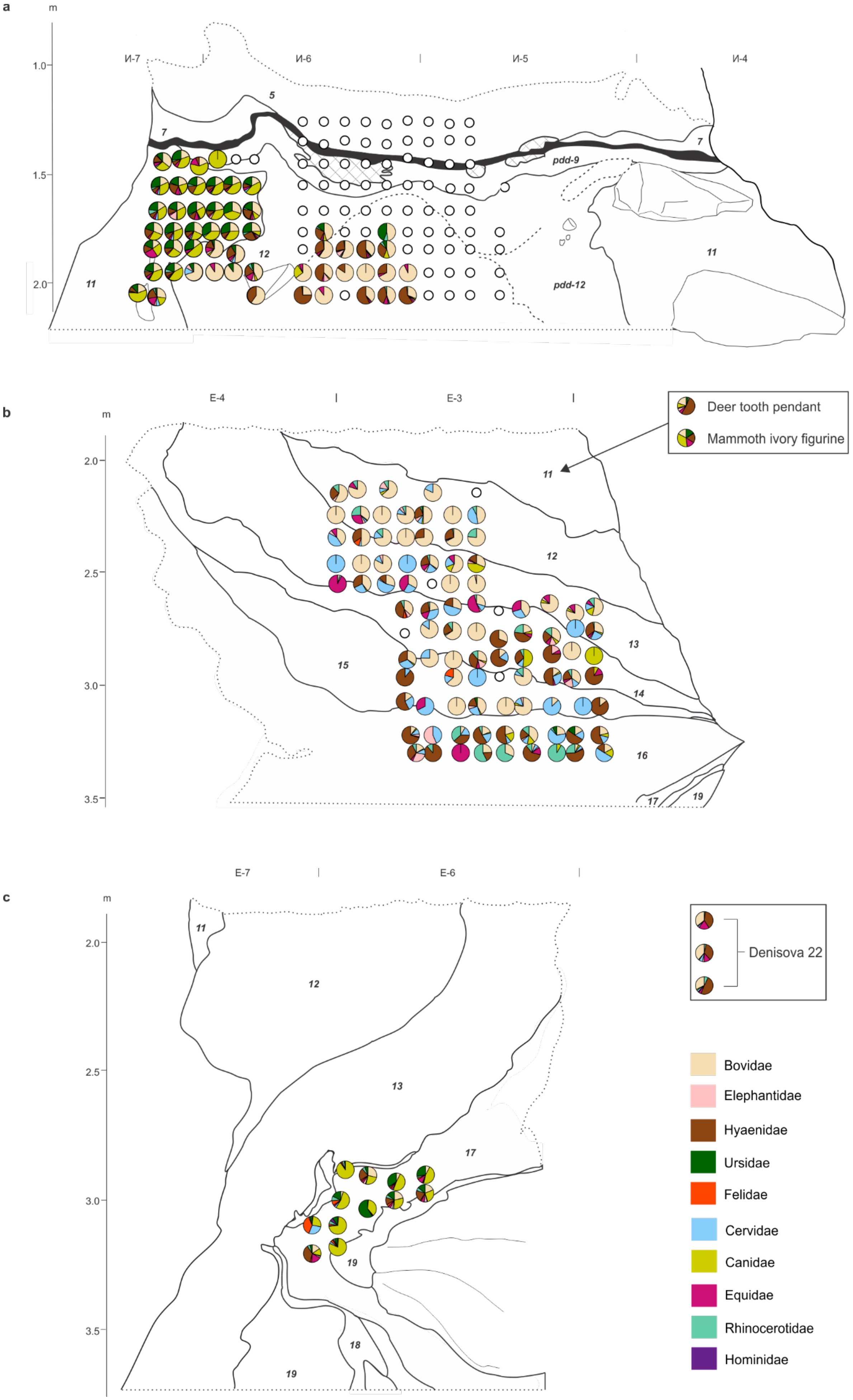

a
m
1.0
1.5
2.0
И-7
И-6
И-5
И-4
5
7
7
pdd-9
pdd-12
11
11
12
b
m
2.0
2.5
3.0
3.5
E-4
E-3
Deer tooth pendant
Mammoth ivory figurine
11
12
13
14
15
16
17
19
c
m
2.0
2.5
3.0
3.5
E-7
E-6
11
12
13
17
18
19
19
Denisova 22
Bovidae
Elephantidae
Hyaenidae
Ursidae
Felidae
Cervidae
Canidae
Equidae
Rhinocerotidae
Hominidae

Supplementary Fig. 27 │ Ancient mammalian DNA in sediment samples from Profiles C and D. a, Results for samples from Profile C. Pie charts show individual sample locations and proportions of mtDNA fragments assigned to specific families of large mammals. White circles denote no ancient mtDNA detected. **b**, Results for samples from Profile D (right side) and the sediments attached to the deer tooth pendant and mammoth ivory figurine. **c**, Results for samples from Profile D (left side) and the sediments directly beneath Denisova 22. The corresponding data for samples from Profiles A and B are shown in Supplementary Fig. 27 of [2].

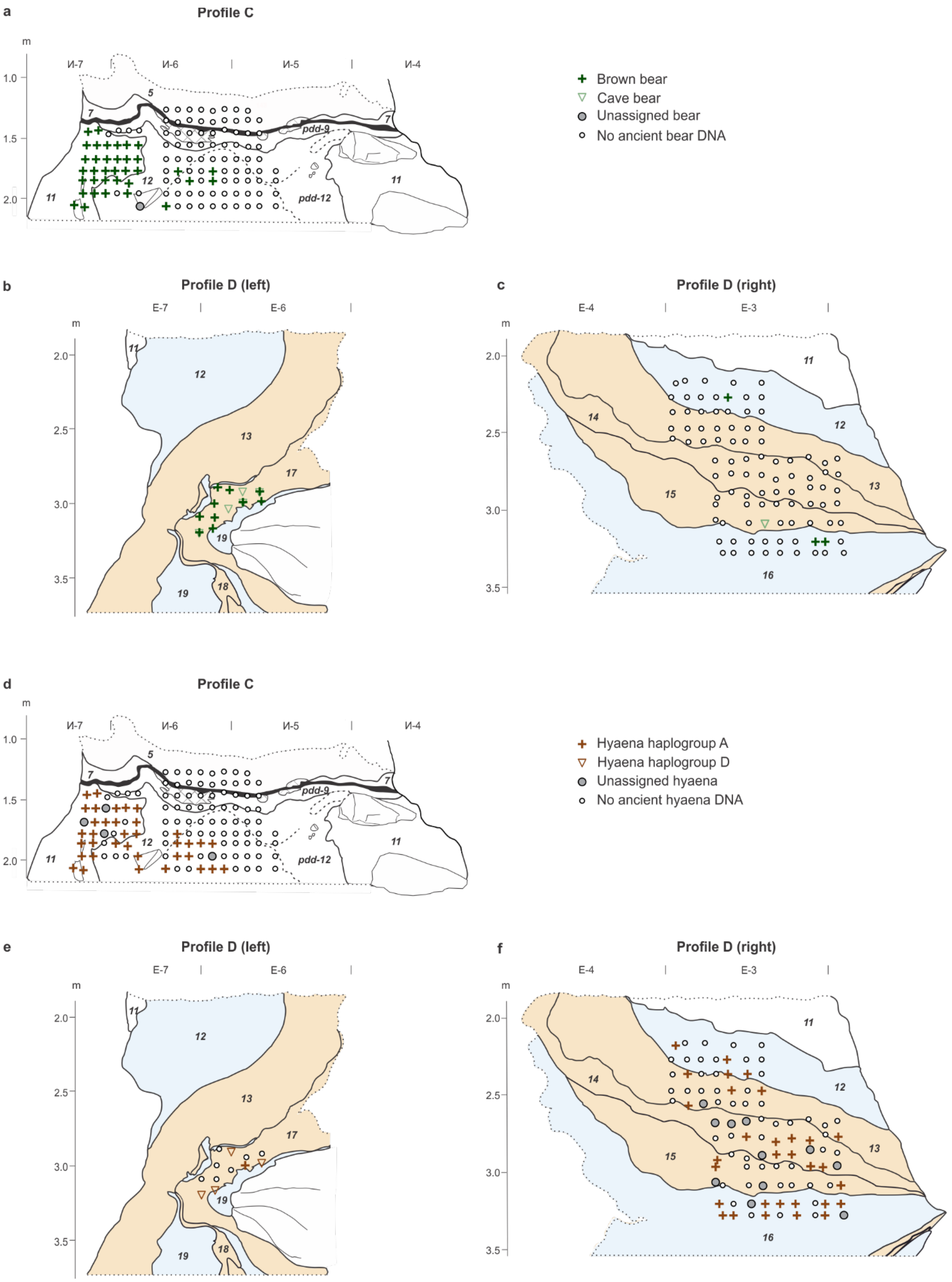

Supplementary Fig. 28 │ Ancient ursid and hyaenid DNA clades in sediment samples from Profiles C and D. a–c, Samples assigned to specific mtDNA groups of ursids in Profiles C, D (left side) and D (right side), respectively: brown bear (dark green crosses), cave bear (light green inverted triangles), unassigned ursid (grey circles) and no ancient ursid DNA (white circles). **d–f**, Samples

assigned to different mtDNA haplogroups of cave hyaenas in Profiles C, D (left side) and D (right side), respectively: haplogroup A (brown crosses), haplogroup D (brown inverted triangles), unassigned hyaenid (grey circles) and no ancient hyaenid DNA (white circles). Layer colours denote relatively warm (orange) or relatively cold (blue) conditions, as in Fig. 10. Layer 11 and all layers in Profile C (**a**, **d**) are shown in white.

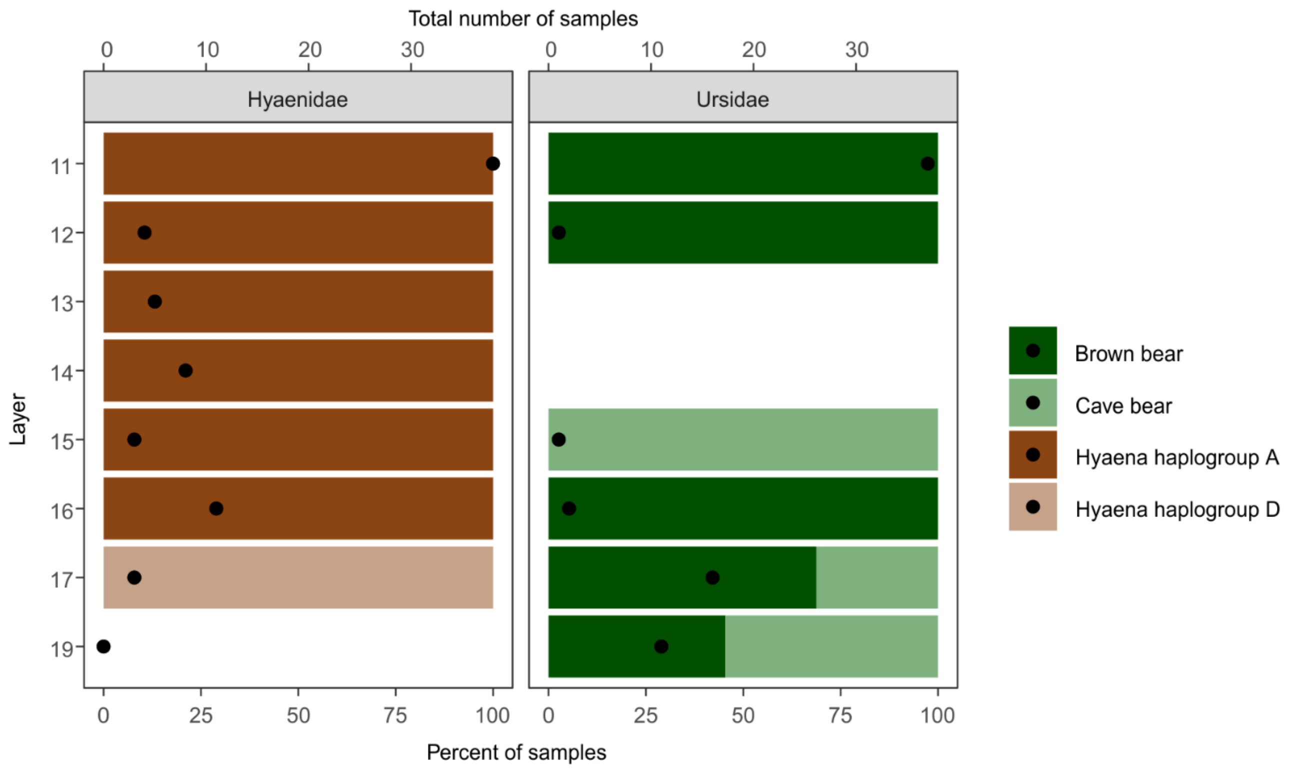

Supplementary Fig. 29 | Ursid and hyaenid mtDNA clades identified in sediment samples from Profiles C and D. Proportions of ancient mtDNA fragments assigned to bear and hyaena clades obtained by averaging across the percentages of fragments assigned to each clade in all samples from a specified layer. Bars are colour-coded by the proportion of samples assigned to each clade: brown bear (dark green), cave bear (light green), cave hyaena haplogroup A (dark brown) and cave hyaena haplogroup D (light brown). Black circles denote the total number of samples assigned to a specific mtDNA clade.

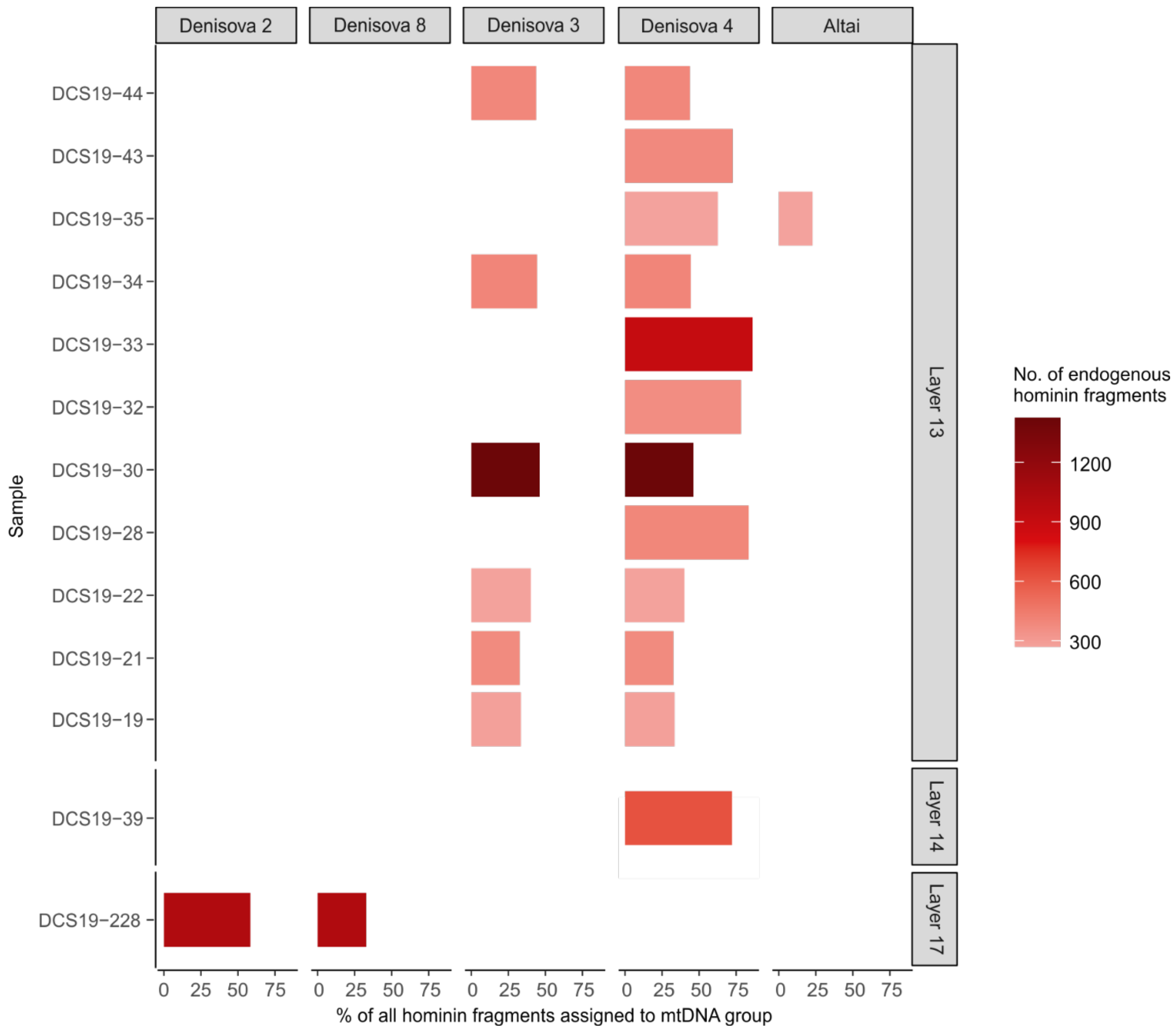

Supplementary Fig. 30 | Denisovan and Neanderthal mtDNA lineage affinities identified in sediment samples from Profile D. Lineages were identified using kallisto[73], based on the assignment of at least 20% of the total identified fragments for samples with at least 250 endogenous hominin sequences. 'Altai' refers to the so-called Altai Neanderthal (Denisova 5), a proximal pedal phalanx recovered from layer 11.4 in East Chamber[75,76].

SUPPLEMENTARY REFERENCES

1. Jacobs, Z. et al. Timing of archaic hominin occupation of Denisova Cave in southern Siberia. *Nature* **565**, 594–599 (2019).
2. Zavala, E. I. et al. Pleistocene sediment DNA reveals hominin and faunal turnovers at Denisova Cave. *Nature* **595**, 399–403 (2021).
3. Shunkov, M. V. et al. The phosphates of Pleistocene–Holocene sediments of the Eastern Gallery of Denisova Cave. *Dokl. Earth Sci.* **478**, 46–50 (2018).
4. Sokol, E. V. et al. Phosphate record in Pleistocene–Holocene sediments from Denisova Cave: Formation mechanisms and archaeological implications. *Minerals* **12**, 553 (2022).
5. Deev, E. et al. Large earthquakes in the Katun Fault zone (Gorny Altai): Paleoseismological and archaeoseismological evidence. *Quat. Sci. Rev.* **203**, 68–89 (2019).
6. Kulik, N. A. et al. Manifestations of neotectonics in karst cavities: Identification experience on the example of Denisova Cave in Gorny Altai. *Theory Pract. Archaeol. Res.* **35**, 193–211 (2023).
7. Morley, M. W. et al. Hominin and animal activities in the microstratigraphic record from Denisova Cave (Altai Mountains, Russia). *Sci. Rep.* **9**, 13785 (2019).
8. Shunkov, M. V. et al. New evidence on the early Middle Paleolithic from Denisova Cave. In *Problems of Archaeology, Ethnography, Anthropology of Siberia and Neighboring Territories* Vol. 26 (eds Derevianko, A. P. et al.) 330–336 (Institute of Archaeology and Ethnography, Siberian Branch of the Russian Academy of Sciences, Novosibirsk, 2020).
9. Shunkov, M. V., Kozlikin, M. B., Chekha, A. M. & Fedorchenko, A. Yu. Paleolithic complexes of the South Chamber of Denisova Cave: 2022 materials. In *Problems of Archaeology, Ethnography, Anthropology of Siberia and Neighboring Territories* Vol. 28 (eds Derevianko, A. P. et al.) 407–414 (Institute of Archaeology and Ethnography, Siberian Branch of the Russian Academy of Sciences, Novosibirsk, 2022).
10. Derevianko, A. P. et al. Recent data on the Middle and Upper Palaeolithic stone tool industries from the Southern Gallery at Denisova Cave. In *Problems of Archaeology, Ethnography, Anthropology of Siberia and Neighboring Territories* Vol. 24 (eds Derevianko, A. P. et al.) 82–86 (Institute of Archaeology and Ethnography, Siberian Branch of the Russian Academy of Sciences, Novosibirsk, 2018).
11. Shunkov et al. Lithic industries of the Middle and Upper Paleolithic from the South Chamber of Denisova Cave: Evidence of 2019. In *Problems of Archaeology, Ethnography, Anthropology of Siberia and Neighboring Territories* Vol. 25 (eds Derevianko, A. P. et al.) 299–305 (Institute of Archaeology and Ethnography, Siberian Branch of the Russian Academy of Sciences, Novosibirsk, 2019).
12. Shunkov, M. V., Kozlikin, M. B. & Mikhienko, V. A. The Middle Paleolithic assemblage from the South Chamber of Denisova Cave: New evidence. In *Problems of Archaeology, Ethnography, Anthropology of Siberia and Neighboring Territories* Vol. 26 (eds Derevianko, A. P. et al.) 322–329 (Institute of Archaeology and Ethnography, Siberian Branch of the Russian Academy of Sciences, Novosibirsk, 2020).
13. Derevianko, A. P. et al. Studies of the Middle and Upper Paleolithic technocomplexes of Denisova Cave in 2021. In *Problems of Archaeology, Ethnography, Anthropology of Siberia and Neighboring Territories* Vol. 27 (eds Derevianko, A. P. et al.) 105–113 (Institute of Archaeology and Ethnography, Siberian Branch of the Russian Academy of Sciences, Novosibirsk, 2021).
14. Derevianko, A. P. et al. New research findings of the Upper Paleolithic assemblage from the South Chamber of Denisova Cave. In *Problems of Archaeology, Ethnography, Anthropology of Siberia and Neighboring Territories* Vol. 23 (eds Derevianko, A. P. et al.) 103–107 (Institute of Archaeology and Ethnography, Siberian Branch of the Russian Academy of Sciences, Novosibirsk, 2017).

15. Shunkov, M. V., Fedorchenko, A. Yu. & Kozlikin, M. B. Bone tools and personal adornments of the Early Upper Paleolithic from the South Chamber of Denisova Cave (collection of 2019). In *Problems of Archaeology, Ethnography, Anthropology of Siberia and Neighboring Territories* Vol. 25 (eds Derevianko, A. P. et al.) 306–312 (Institute of Archaeology and Ethnography, Siberian Branch of the Russian Academy of Sciences, Novosibirsk, 2019).
16. Baumann, M., Kozlikin, M. B., Plisson, H. & Shunkov, M. V. Early Upper Paleolithic unshaped bone tools from the Southern Chamber of Denisova Cave. In *Problems of Archaeology, Ethnography, Anthropology of Siberia and Neighboring Territories* Vol. 23 (eds Derevianko, A. P. et al.) 50–54 (Institute of Archaeology and Ethnography, Siberian Branch of the Russian Academy of Sciences, Novosibirsk, 2017).
17. Baumann, M., Fedorchenko, A. Yu., Kozlikin, M. B., Plisson, H. & Shunkov, M. V. The Middle and Upper Paleolithic bone tools from South Chamber of Denisova Cave. In *Problems of Archaeology, Ethnography, Anthropology of Siberia and Neighboring Territories* Vol. 24 (eds Derevianko, A. P. et al.) 32–36 (Institute of Archaeology and Ethnography, Siberian Branch of the Russian Academy of Sciences, Novosibirsk, 2018).
18. Shunkov, M. V., Kozlikin, M. B. & Fedorchenko, A. Yu. Bone tools in the Middle and Upper Paleolithic complexes of Denisova Cave: 2021 materials. In *Problems of Archaeology, Ethnography, Anthropology of Siberia and Neighboring Territories* Vol. 27 (eds Derevianko, A. P. et al.) 355–361 (Institute of Archaeology and Ethnography, Siberian Branch of the Russian Academy of Sciences, Novosibirsk, 2021).
19. Shunkov, M. V., Fedorchenko, A. Yu. & Kozlikin, M. B. Early Upper Paleolithic ivory bracelet from Denisova Cave. In *Problems of Archaeology, Ethnography, Anthropology of Siberia and Neighboring Territories* Vol. 23 (eds Derevianko, A. P. et al.) 255–258 (Institute of Archaeology and Ethnography, Siberian Branch of the Russian Academy of Sciences, Novosibirsk, 2017).
20. Shunkov, M. V., Fedorchenko, A. Yu. & Kozlikin, M. B. Early Upper Paleolithic objects of bone from the South Chamber of Denisova Cave (collection of 2017). In *Problems of Archaeology, Ethnography, Anthropology of Siberia and Neighboring Territories* Vol. 23 (eds Derevianko, A. P. et al.) 259–262 (Institute of Archaeology and Ethnography, Siberian Branch of the Russian Academy of Sciences, Novosibirsk, 2017).
21. Derevianko, A. P., Shunkov, M. V., Fedorchenko, A. Yu. & Kozlikin, M. B. A Palaeolithic diadem from Denisova Cave. In *Problems of Archaeology, Ethnography, Anthropology of Siberia and Neighboring Territories* Vol. 24 (eds Derevianko, A. P. et al.) 87–90 (Institute of Archaeology and Ethnography, Siberian Branch of the Russian Academy of Sciences, Novosibirsk, 2018).
22. Shunkov, M. V., Fedorchenko, A. Yu. & Kozlikin, M. B. The early Upper Palaeolithic personal ornaments from the Southern Chamber of Denisova Cave. In *Problems of Archaeology, Ethnography, Anthropology of Siberia and Neighboring Territories* Vol. 24 (eds Derevianko, A. P. et al.) 198–202 (Institute of Archaeology and Ethnography, Siberian Branch of the Russian Academy of Sciences, Novosibirsk, 2018).
23. Derevianko, A. P., Shunkov, M. V., Fedorchenko, A. Yu. & Kozlikin, M. B. Paleolithic sculpture from Denisova Cave. In *Problems of Archaeology, Ethnography, Anthropology of Siberia and Neighboring Territories* Vol. 25 (eds Derevianko, A. P. et al.) 103–108 (Institute of Archaeology and Ethnography, Siberian Branch of the Russian Academy of Sciences, Novosibirsk, 2019).
24. Shunkov, M. V., Fedorchenko, A. Yu. & Kozlikin, M. B. Upper Paleolithic ornaments from the South Chamber of Denisova Cave: 2021 collection. In *Problems of Archaeology, Ethnography, Anthropology of Siberia and Neighboring Territories* Vol. 27 (eds Derevianko, A. P. et al.) 362–368 (Institute of Archaeology and Ethnography, Siberian Branch of the Russian Academy of Sciences, Novosibirsk, 2021).
25. Vasiliev, S. K., Kozlikin, M. B. & Shunkov, M. V. Faunal remains from the Pleistocene deposits in the Southern Chamber of Denisova Cave (evidence of 2019). In *Problems of*

Archaeology, Ethnography, Anthropology of Siberia and Neighboring Territories Vol. 26 (eds Derevianko, A. P. et al.) 37–42 (Institute of Archaeology and Ethnography, Siberian Branch of the Russian Academy of Sciences, Novosibirsk, 2020).
26. Vasiliev, S. K., Shunkov, M. V. & Kozlikin, M. B. Bone remains from the Pleistocene deposits in the South Chamber of Denisova Cave: New evidence. In *Problems of Archaeology, Ethnography, Anthropology of Siberia and Neighboring Territories* Vol. 27 (eds Derevianko, A. P. et al.) 83–90 (Institute of Archaeology and Ethnography, Siberian Branch of the Russian Academy of Sciences, Novosibirsk, 2021).
27. Vasiliev, S. K., Shunkov, M. V. & Kozlikin, M. B. New data on Late Pleistocene megafauna from Denisova Cave. In *Problems of Archaeology, Ethnography, Anthropology of Siberia and Neighboring Territories* Vol. 28 (eds Derevianko, A. P. et al.) 82–88 (Institute of Archaeology and Ethnography, Siberian Branch of the Russian Academy of Sciences, Novosibirsk, 2022).
28. Agadjanian, A. K., Shunkov, M. V. & Kozlikin, M. B. Dynamics of small vertebrate communities from the Pleistocene deposits of the Denisova Cave South Chamber. In *Problems of Archaeology, Ethnography, Anthropology of Siberia and Neighboring Territories* Vol. 28 (eds Derevianko, A. P. et al.) 7–12 (Institute of Archaeology and Ethnography, Siberian Branch of the Russian Academy of Sciences, Novosibirsk, 2022).
29. Agadjanian, A. K., Shunkov, M. V. & Kozlikin, M. B. Dynamics of the microtheriofauna ecological composition from Pleistocene deposits in the South Chamber of Denisova Cave. In *Problems of Archaeology, Ethnography, Anthropology of Siberia and Neighboring Territories* Vol. 29 (eds Derevianko, A. P. et al.) 7–11 (Institute of Archaeology and Ethnography, Siberian Branch of the Russian Academy of Sciences, Novosibirsk, 2023).
30. Huntley, D. J., Godfrey-Smith, D. I. & Thewalt, M. L. W. Optical dating of sediments. *Nature* **313**, 105–107 (1985).
31. Roberts, R. G. et al. Optical dating in archaeology: thirty years in retrospect and grand challenges for the future. *J. Archaeol. Sci.* **56**, 41–60 (2015).
32. Murray, A. et al. Optically stimulated luminescence dating using quartz. *Nat. Rev. Methods Primers* **1**, 72 (2021).
33. Mahan, S. A. et al. Guide for interpreting and reporting luminescence dating results. *Geol. Soc. Am. Bull.* **135**, 1480–1502 (2023).
34. Massilani, D. et al. Microstratigraphic preservation of ancient faunal and hominin DNA in Pleistocene cave sediments. *Proc. Natl Acad. Sci. USA* **119**, e2113666118 (2022)
35. Duller, G. A. T. Single-grain optical dating of Quaternary sediments: why aliquot size matters in luminescence dating. *Boreas* **37**, 589–612 (2008).
36. Li, B., Jacobs, Z. & Roberts, R. G. An improved multiple-aliquot regenerative-dose (MAR) procedure for post-IR IRSL dating of K-feldspar. *Anc. TL* **35**, 1–10 (2017).
37. Bøtter-Jensen, L., Andersen, C. E., Duller, G. A. T. & Murray, A. S. Developments in radiation, stimulation and observation facilities in luminescence measurements. *Radiat. Meas.* **37**, 535–541 (2003).
38. Autzen, M., Andersen, C. E., Bailey, M. & Murray, A. S. Calibration quartz: An update on dose calculations for luminescence dating. *Radiat. Meas.* **157**, 106828 (2022).
39. Galbraith, R. F., Roberts, R. G., Laslett, G. M., Yoshida, H. & Olley, J. M. Optical dating of single and multiple grains of quartz from Jinmium rock shelter, northern Australia: Part I, experimental design and statistical models. *Archaeometry* **41**, 339–364 (1999).
40. Murray, A. S. & Wintle, A. G. Luminescence dating of quartz using an improved single-aliquot regenerative-dose protocol. *Radiat. Meas.* **32**, 57–73 (2000).
41. Li, B., Jacobs, Z., Roberts, R. G., Galbraith, R. & Peng, J. Variability in quartz OSL signals caused by measurement uncertainties: Problems and solutions. *Quat. Geochronol.* **41**, 11–25 (2017).
42. Li, B., Jacobs, Z. & Roberts, R. G. Validation of the L_nT_n method for D_e determination in optical dating of K-feldspar and quartz. *Quat. Geochronol.* **58**, 101066 (2020).

43. Guralnik, B. et al. Radiation-induced growth and isothermal decay of infrared-stimulated luminescence from feldspar. *Radiat. Meas.* **81**, 224–231 (2015).
44. Li, B., Jacobs, Z., Roberts, R. G. Investigation of the applicability of standardised growth curves for OSL dating of quartz from Haua Fteah cave, Libya. *Quat. Geochronol.* **35**, 1–15 (2016).
45. Fu, X. et al. Establishing standardised growth curves (SGCs) for OSL signals from individual grains of quartz: A continental-scale case study. *Quat. Geochronol.* **60**, 101107 (2020).
46. Galbraith, R. F. & Roberts, R. G. Statistical aspects of equivalent dose and error calculation and display in OSL dating: An overview and some recommendations. *Quat. Geochronol.* **11**, 1–27 (2012).
47. Thomsen, K. J., Murray, A. S., Jain, M. & Bøtter-Jensen, L. Laboratory fading rates of various luminescence signals from feldspar-rich sediment extracts. *Radiat. Meas.* **43**, 1474–1486 (2008).
48. Blegen, N. et al. Distal tephras of the eastern Lake Victoria basin, equatorial East Africa: correlations, chronology and a context for early modern humans. *Quat. Sci. Rev.* **122**, 89–111 (2015).
49. Li, B. & Li, S.-H. A reply to the comments by Thomsen *et al.* on “Luminescence dating of K-feldspar from sediments: a protocol without anomalous fading correction”. *Quat. Geochronol.* **8**, 49–51 (2012).
50. Li, B., Jacobs, Z., Roberts, R. G. & Li, S.-H. Single-grain dating of potassium-rich feldspar grains: Towards a global standardised growth curve for the post-IR IRSL signal. *Quat. Geochronol.* **45**, 23–36 (2018).
51. Roberts, R. G., Galbraith, R. F., Yoshida, H., Laslett, G. M. & Olley, J. M. Distinguishing dose populations in sediment mixtures: A test of single-grain optical dating procedures using mixtures of laboratory-dosed quartz. *Radiat. Meas.* **32**, 459–465 (2000).
52. Peng, J., Dong, Z., Han, F., Long, H. & Liu, X. R package *numOSL*: numeric routines for optically stimulated luminescence dating. *Anc. TL* **31**, 41–48 (2013).
53. Peng, J. & Li, B. Single-aliquot regenerative-dose (SAR) and standardised growth curve (SGC) equivalent dose determination in a batch model using the R package ‘numOSL’. *Anc. TL* **35**, 32–53 (2017).
54. Kreutzer, S. et al. Introducing an R package for luminescence dating analysis. *Anc. TL* **30**, 1–8 (2012).
55. Kreutzer, S. et al. *Luminescence*: Comprehensive Luminescence Dating Data Analysis. R package version 0.9.21, https://CRAN.R-project.org/package=Luminescence (2023).
56. Olley, J. M., Murray, A. S. & Roberts, R. G. The effects of disequilibria in the uranium and thorium decay chains on burial dose rates in fluvial sediments. *Quat. Sci. Rev.* **15**, 751–760 (1996).
57. Olley, J. M., Roberts, R. G. & Murray, A. S. Disequilibria in the uranium decay series in sedimentary deposits at Allen’s Cave, Nullarbor Plain, Australia: implications for dose rate determinations. *Radiat. Meas.* **27**, 433–443 (1997).
58. Bøtter-Jensen, L. & Mejdahl, V. Assessment of beta dose-rate using a GM multicounter system. *Nucl. Tracks Radiat. Meas.* **14**, 187–191 (1988).
59. Jacobs, Z. & Roberts, R. G. An improved single grain OSL chronology for the sedimentary deposits from Diepkloof Rockshelter, Western Cape, South Africa. *J. Archaeol. Sci.* **63**, 175–192 (2015).
60. Cunningham, A. C., Murray, A. S., Armitage, S. J. & Autzen, M. High-precision natural dose rate estimates through beta counting. *Radiat. Meas.* **120**, 209–214 (2018).
61. Aitken, M. J. *Thermoluminescence Dating* (Academic Press, London, 1985).
62. Fu, X. et al. Beta dose heterogeneity in sediment samples measured using a Timepix pixelated detector and its implications for optical dating of individual mineral grains. *Quat. Geochronol.* **68**, 101254 (2022).
63. Prescott, J. R. & Hutton, J. T. Cosmic ray contributions to dose rates for luminescence and ESR dating: large depths and long-term time variations. *Radiat. Meas.* **23**, 497–500 (1994).

64. O'Gorman, K., Brink, F., Tanner, D., Li, B. & Jacobs, Z., 2021. Calibration of a QEM-EDS system for rapid determination of potassium concentrations of feldspar grains used in optical dating. *Quat. Geochronol.* **61**, 101123 (2021).
65. Huntley, D. J. & Hancock, R. G. V. The Rb contents of the K-feldspars being measured in optical dating. *Anc. TL* **19**, 43–46 (2001).
66. Cheng. T., Zhang, D., Zhao, H., Yang, S. & Li, B. Bleachability of pIRIR signal from single-grain K-feldspar. *Quat. Geochronol.* **71**, 101321 (2022).
67. Zhang. J., Guralnik, B., Tsukamoto, S., Ankjærgaard, C. & Reimann, T. The bleaching limits of IRSL signals at various stimulation temperatures and their potential inference of the pre-burial light exposure duration. *Front. Earth Sci.* **10**, 933131 (2023).
68. Bronk Ramsey, C. Bayesian analysis of radiocarbon dates. *Radiocarbon* **51**, 337–360 (2009).
69. Bronk Ramsey, C. & Lee, S. Recent and planned developments of the program OxCal. *Radiocarbon* **55**, 720–730 (2013).
70. Bronk Ramsey, C. Dealing with outliers and offsets in radiocarbon dating. *Radiocarbon* **51**, 1023–1045 (2009).
71. Gansauge, M. T., Aximu-Petri, A., Nagel, S. & Meyer, M. Manual and automated preparation of single-stranded DNA libraries for the sequencing of DNA from ancient biological remains and other sources of highly degraded DNA. *Nat. Protocols* **15**, 2279–2300 (2020).
72. Essel, E. et al. Ancient human DNA recovered from a Palaeolithic pendant. *Nature* **618**, 328–332 (2023).
73. Bray, N. L., Pimentel, H., Melsted, P. & Pachter, L. Near-optimal probabilistic RNA-seq quantification. *Nat. Biotechnol.* **34**, 525–527 (2016).
74. Vernot, B. et al. Unearthing Neanderthal population history using nuclear and mitochondrial DNA from cave sediments. *Science* **372**, eabf1667 (2021).
75. Mednikova, M. B. A proximal pedal phalanx of a Paleolithic hominin from Denisova Cave, Altai. *Archaeol. Ethnol. Anthropol. Eurasia* **39/1**, 129–138 (2011).
76. Prüfer, K. et al. The complete genome sequence of a Neanderthal from the Altai Mountains. *Nature* **505**, 43–49 (2014).
77. Lisiecki, L. E. & Raymo, M. E. A Pliocene–Pleistocene stack of 57 globally distributed benthic $\delta^{18}O$ records. *Paleoceanography* **20**, PA1003 (2005).
