# Supplementary material for: Pleistocene chronology and history of hominins and fauna at Denisova Cave
Source: Nat Commun. 2025 May 21;16:4738. doi: 10.1038/s41467-025-60140-6 (PMC12095498; doi:10.1038/s41467-025-60140-6)
Supplement: Supplementary file 2 — Description of Additional Supplementary Files [file 41467_2025_60140_MOESM2_ESM.pdf]

Description of Additional Supplementary Files

SUPPLEMENTARY DATA

Supplementary Data 1 | Stratigraphy and sedimentology of Pleistocene deposits in South Chamber. Layer numbers in parentheses were used previously (Jacobs et al., 2019; Zavala et al., 2021).

Supplementary Data 2 | Number of artefacts recovered from the Pleistocene deposits in South Chamber, 2017–2022. Layer colours denote archaeological phases: early Middle Palaeolithic (dark green), middle Middle Palaeolithic (light green) and Upper Palaeolithic (light orange). Layer 11 could not be divided stratigraphically into different stages of the Upper Palaeolithic during excavation.

Supplementary Data 3 | Proportions (in %) of large mammals in the Pleistocene deposits in South Chamber (Vasiliev et al., 2020, 2021). Dashes denote taxa not represented or with proportions of less than 0.05%.

Supplementary Data 4 | Proportions (in %) of biotope groups of large mammals in the Pleistocene deposits in South Chamber.

Supplementary Data 5 | Proportions (in %) of small vertebrates in the Pleistocene deposits in South Chamber (Agadjanian et al., 2022, 2023). Dashes denote taxa not represented or with proportions of less than 0.05%.

Supplementary Data 6 | Proportions (in %) of small terrestrial vertebrates in the Pleistocene deposits in South Chamber. Dashes denote taxa not represented or with proportions of less than 0.05%. Except for Chiroptera, Aves, Pisces and unspecified Carnivora, the taxa listed here are the same as in Supplementary Data 5 and were used to infer the local climatic conditions, following (Jacobs et al., 2019).

Supplementary Data 7 | Number of individual quartz grains measured, rejected and accepted for D_e determination for each sample from South Chamber, together with the reasons for rejection. The L_nT_n method was used for the six samples shown in italics (additional details in Supplementary Data 10); rejection criteria 6 and 7 do not apply to this method.

Supplementary Data 8 | Number of individual K-feldspar grains measured using an abbreviated pIRIR procedure that included measurements of only the natural signal, a single regenerative-dose signal and their corresponding test-dose signals. The number of grains rejected and accepted for D_e determination, and the reasons for rejection, are also listed. Samples were measured using grains of either 180–212 or 150–180 µm in diameter; the latter samples are marked *. 'T_n threshold' is the T_n signal intensity above which a 'plateau' in D_e values or re-normalized L_n/T_n ratios is reached.

Supplementary Data 9 | Dose rate data, equivalent dose (D_e) and overdispersion (OD) values, and optical ages for quartz (Q) and K-feldspar (KF) samples from Profiles A–D in South Chamber. For each sample, the measured moisture content is shown in brackets after the value (± 1σ uncertainty) used for dose rate and age determination. Total dose rates include a cosmic-ray contribution 0.030 ± 0.005 Gy/ka for all samples. The number of grains refers to the total number of individual grains accepted for D_e determination after rejecting grains with unsuitable OSL or pIRIR properties. Sample D_e values were estimated using the central age model (CAM), the CAM after rejecting any statistical outliers identified using the normalised median absolute deviation (nMAD CAM), the finite mixture model (FMM) or, for four samples, the minimum age model (MAM) (Galbraith and Roberts, 2012; Galbraith et al., 1999; Jacobs et al., 2019; Li et al. 2020; Roberts et al. (2000). OD values refer to the

overdispersion in D_e or least-squares normalized L_n/T_n values estimated using the CAM, or for the nMAD CAM, following outlier rejection. For the other two models, the OD represents the optimal value obtained from fitting the FMM (as determined using the Bayesian Information Criterion) or the value added in quadrature to each of D_e measurement uncertainties before running the MAM. FMM components containing at least 70% of accepted grains are highlighted in bold. Samples highlighted in blue, red and green represent Group A, B and C samples, respectively (see Supplementary Section 2.3 for details). For all samples, the age uncertainties shown after the ± symbol represent the total uncertainties (random plus systematic errors) at 1σ and include a systematic error of 2% to allow for any bias associated with calibration of the laboratory beta source; the uncertainties shown in brackets are the total random-only errors at 1σ.

Supplementary Data 10 │ Summary data for quartz samples measured using the L_nT_n method. For each group of grains, 'N' is the number of grains accepted for initial D_e determination (see Supplementary Data 7) and 'n' is the number used for final D_e estimation using the CAM, nMAD CAM or FMM (Supplementary Figs. 13–18). OD values refer to the overdispersion in D_e values estimated using the CAM or nMAD CAM, or the optimal value obtained from fitting the FMM.

Supplementary Data 11 | Area proportions (in %) of mineral phases within grains for samples DCS17-3 and DCS17-6 derived from QEM–EDS. Phases shown in italics are the estimated true proportions of feldspar phase. The true proportion of orthoclase/microcline is assumed to be equal to the sum of spectral fitting to the orthoclase and sanidine standards, and the true proportion of albite is assumed to be equal to the sum of spectral fitting to the anorthoclase, albite and labradorite reference standards (O'Gorman et al., 2021).

Supplementary Data 12 | Fractional contributions (mean ± standard deviation, in %) of external dose rate components and internal beta dose rates to the total dose rates for quartz and K-feldspar samples. Data are shown for quartz grains with diameters of 180–212 µm and K-feldspar grains with diameters of 180–212 and 150–180 µm.

Supplementary Data 13 | Weighted mean age estimates for samples with both OSL (quartz) and pIRIR (K-feldspar) ages included in Bayesian age models A and/or B. The age uncertainties are the total random-only errors at 1σ.

Supplementary Data 14 | Bayesian age model A. Optical ages for South Chamber ('Unmodelled age ranges') and corresponding Bayesian model estimates ('Modelled age ranges') at 68.2% and 95.4% probability, obtained using the OxCal4.4.4 platform. Modelled start and end ages for each stratigraphic layer (or combination of layers) are highlighted in bold and italics within the grey bands. Modelled intervals between stratigraphic layers are shown in grey and italics between the grey bands. All ages are given in years and rounded off to the closest decade.

Supplementary Data 15 | Bayesian age model B. Optical ages for South Chamber ('Unmodelled age ranges') and corresponding Bayesian model estimates ('Modelled age ranges') at 68.2% and 95.4% probability, obtained using the OxCal.4.4.4 platform. Modelled start and end ages for each archaeological phase (early Middle Palaeolithic, eMP; middle Middle Palaeolithic, mMP; Upper Palaeolithic, UP) or stratigraphic layers (post-UP deposits) are highlighted in bold and italics within the grey bands. A modelled interval between the end of the pre-occupation deposits and the start of the eMP is shown in grey and italics between the grey bands. All ages are given in years and rounded off to the closest decade.

Supplementary Data 16 | Summary of sediment samples that underwent genetic analysis.

Supplementary Data 17 | Summary statistics for sequencing data after enrichment for mammalian mtDNA.

Supplementary Data 18 | Summary statistics for sequencing data after enrichment for human mtDNA.

SUPPLEMENTARY CODES

Supplementary Code 1 | CQL code for Bayesian age model A in Fig. 5.

Supplementary Code 2 | CQL code for Bayesian age model B in Supplementary Fig. 24.
