# Supplementary material for: Pleistocene chronology and history of hominins and fauna at Denisova Cave
Source: Nat Commun. 2025 May 21;16:4738. doi: 10.1038/s41467-025-60140-6 (PMC12095498; doi:10.1038/s41467-025-60140-6)
Supplement: Supplementary file 22 — Reporting Summary [file 41467_2025_60140_MOESM22_ESM.pdf]

nature portfolio

Corresponding author(s): Zenobia Jacobs, Elena I. Zavala, Matthias Meyer, Richard G. Roberts

Last updated by author(s): May 13, 2025

Reporting Summary

Nature Portfolio wishes to improve the reproducibility of the work that we publish. This form provides structure for consistency and transparency in reporting. For further information on Nature Portfolio policies, see our Editorial Policies and the Editorial Policy Checklist.

Statistics

For all statistical analyses, confirm that the following items are present in the figure legend, table legend, main text, or Methods section.

n/a	Confirmed	
☐	☒	The exact sample size (n) for each experimental group/condition, given as a discrete number and unit of measurement
☐	☒	A statement on whether measurements were taken from distinct samples or whether the same sample was measured repeatedly
☒	☐	The statistical test(s) used AND whether they are one- or two-sided<br>*Only common tests should be described solely by name; describe more complex techniques in the Methods section.*
☐	☒	A description of all covariates tested
☐	☒	A description of any assumptions or corrections, such as tests of normality and adjustment for multiple comparisons
☐	☒	A full description of the statistical parameters including central tendency (e.g. means) or other basic estimates (e.g. regression coefficient) AND variation (e.g. standard deviation) or associated estimates of uncertainty (e.g. confidence intervals)
☒	☐	For null hypothesis testing, the test statistic (e.g. F, t, r) with confidence intervals, effect sizes, degrees of freedom and P value noted<br>*Give P values as exact values whenever suitable.*
☐	☒	For Bayesian analysis, information on the choice of priors and Markov chain Monte Carlo settings
☒	☐	For hierarchical and complex designs, identification of the appropriate level for tests and full reporting of outcomes
☒	☐	Estimates of effect sizes (e.g. Cohen's d, Pearson's r), indicating how they were calculated

Our web collection on statistics for biologists contains articles on many of the points above.

Software and code

Policy information about availability of computer code

Data collection	No software was used for the collection of data
Data analysis	Optical dating data was analyzed and visualised using R version 4.3.2 and two R packages called 'Luminescence' and 'numOSL'. Data was also visualized using CorelDRAW. Sediment DNA data was analysed using the leehom package that is available at https://bioinf.eva.mpg.de/, bam-rmdup package that is available at https://github.com/mpieva/biohazard-tools, MEGAN, BLAST, kallisto and R version 3.5.1.

For manuscripts utilizing custom algorithms or software that are central to the research but not yet described in published literature, software must be made available to editors and reviewers. We strongly encourage code deposition in a community repository (e.g. GitHub). See the Nature Portfolio guidelines for submitting code & software for further information.

Data

Policy information about availability of data

All manuscripts must include a data availability statement. This statement should provide the following information, where applicable:

- Accession codes, unique identifiers, or web links for publicly available datasets
- A description of any restrictions on data availability
- For clinical datasets or third party data, please ensure that the statement adheres to our policy

All data for optical dating are provided in Supplementary Figures and Supplementary Data. Previously published data are provided in Supplementary Figures and Supplementary Tables in 1. All optical dating data and samples are stored in the Optical Dating Facility at the University of Wollongong. Any other relevant data are

available from Z.J. and B.L. upon reasonable request.
All sequence data from the mammalian and human mtDNA captures are available in the European Nucleotide Archive under accession number PRJEB80323 (https://www.ebi.ac.uk/ena/browser/view/PRJEB80323). Previously published data are provided in 3 and are available as follows: mtDNA consensus sequences reported from Main Chamber layers 19 (M65) and 20 (M71), and from East Chamber layers 11.4 (E202) and 11.4/12.1 (E213), are available in the Dryad digital repository (https://doi.org/10.5061/dryad.k3j9kd567), and the raw data for each mammalian mtDNA and human mtDNA enriched library are available in the European Nucleotide Archive under accession number PRJEB44036 (https://www.ebi.ac.uk/ena/browser/view/PRJEB44036). All sediment DNA data and samples

Research involving human participants, their data, or biological material

Policy information about studies with human participants or human data. See also policy information about sex, gender (identity/presentation), and sexual orientation and race, ethnicity and racism.

Reporting on sex and gender	Not applicable
Reporting on race, ethnicity, or other socially relevant groupings	Not applicable
Population characteristics	Not applicable
Recruitment	Not applicable
Ethics oversight	Not applicable

Note that full information on the approval of the study protocol must also be provided in the manuscript.

Field-specific reporting

Please select the one below that is the best fit for your research. If you are not sure, read the appropriate sections before making your selection.

☐ Life sciences ☐ Behavioural & social sciences ☒ Ecological, evolutionary & environmental sciences

For a reference copy of the document with all sections, see nature.com/documents/nr-reporting-summary-flat.pdf

Ecological, evolutionary & environmental sciences study design

All studies must disclose on these points even when the disclosure is negative.

Study description	Sediment samples were collected for optical dating and sediment DNA from South Chamber in Denisova Cave
Research sample	Sediment samples were collected from vertical profiles exposed during excavation of the cave deposits. Samples were collected from stratigraphic layers at suitable locations. Artefacts, hominin (Denisovan and Neanderthal) fossils, and the remains of the fauna and flora have been collected previously, with all relevant references cited in the paper.
Sampling strategy	Our aim was to collect and date samples from as many of the Pleistocene sedimentary layers to obtain a detailed horizontal and vertical coverage of the sediments from South Chamber. Sediment DNA samples were collected in a grid-like fashion from the same vertical profiles.
Data collection	Samples were collected for optical dating by Z.J., R.G.R., B.L and K.O. by removing sediment from profiles using a metal corer under red light at night time. A sub-sample was also collected for measurement of the radioactivity of the sediment. Samples for luminescence data were measured by Z.J., B.L. and K.O. using Risoe TL/OSL readers and analysed by Z.J. and B.L. using a range of different customised software. Samples for measurement of the radioactivity of each samples was conducted by Z.J., R.G.R. and K.O. in the field using a field gamma spectrometer and in the laboratory using a GM-25-5 beta counter. A check on the beta dose rates were also obtained by an independent laboratory through measurements of potassium, uranium and thorium using inductively-coupled plasma mass spectrometry. Data for sediments and stratigraphy, artefacts, hominin (Denisovan and Neanderthal) fossils, and the remains of the fauna and flora have been collected by our Russian co-authors. Soil micromorphology samples were collected by P.G using plaster bandages. Sediment DNA were collected by Z.J., B.L., K.O. and R.G.R in the field and analysed in the laboratory by E.I.Z.
Timing and spatial scale	The project was initiated in 2011 after which a memorandum of understanding was signed between the University of Wollongong and the Russian Academy of Sciences, Siberian Branch. Our first sampling trip under this MOU commenced in 2012. It initially took some time to ship samples from Russia to Australia (~9-12 months from sampling) and we needed time to develop appropriate measurement procedures for the samples. A sampling field trip for collection of additional samples to fill gaps and to target freshly excavated areas then took place in alternate years. So, the whole project was necessarily protracted. The sampling frequency was strategically planned based on the progress of archaeological excavation at Denisova Cave, and the ongoing dating work in the laboratory. Artefacts, hominin fossils, and the remains of the fauna and flora have been collected at various times over the last 40 years.
Data exclusions	All data collected were analysed and contributed to the final conclusions.

Reproducibility	This study involved measurements of multiple, individual quartz and K-feldspar grains, yielding distributions of equivalent-dose I estimates from which the weighted mean was calculated using well-established statistical models. For a few samples, multiple aliquot measurements were required to estimate the equivalent dose. The distributions of equivalent-dose values for all samples are shown in Supplementary Information. Reproducibility of the beta dose rates was also assessed directly using independent methods, as described in Supplementary Information. A comparison of quartz and K-feldspar ages displayed. All attempts at replication were successful.
Randomization	No randomization was done. All samples were evaluated for the presence of ancient faunal and hominin mitochondrial DNA and analysis continued for those that contained ancient DNA. All samples were dated using optical dating techniques.
Blinding	No blinding was performed for this study as it was not relevant. For sediment DNA samples blinding was not relevant for data collection as samples were selected based on their location within the stratigraphy. Blinding was also not relevant for downstream analysis as previously established analysis pipelines was used for the processing of the data and results were interpreted based on expectations from and comparisons to previously published ancient and modern mitochondrial genomes.

Did the study involve field work? ☒ Yes ☐ No

Field work, collection and transport

Field conditions	Samples for optical dating were collected in August 2012, 2016, 2017, 2018 and 2019 from inside Denisova Cave, where conditions were cool and dry. Samples for sediment DNA analyses were collected in 2017, 2018 and 2019 from inside Denisova Cave.
Location	Denisova Cave is located at 51 "23'51.3" N, 84"40'34.3"E and elevated 670 m above sea level.
Access & import/export	Excavations were conducted and samples collected during the 2012, 2016, 2017, 2018 and 2019 field seasons. Permits to conduct archaeological excavations at Denisova Cave were issued to M.V.S. by the Ministry of Culture of the Russian Federation for excavations as follows: permit number 210 (issued 15 May 2012), permit number 646 (issued 31 May 2016), permit number 538 (issued 31 May 2017), permit number 1193 (issued 11 May 2018) and permit number 0432-2019 (issued 6 May 2019).<br><br>Sediment samples were exported from Russia to Australia under appropriate Russian permits issued to Michael V. Shunkov (Institute of Archaeology and Ethnography, Novosibirsk, Russia):<br><br>For samples collected in August 2012<br>Name of issuing authority: the Novosibirsk Customs Office<br>Date of issue: 27/08/2013<br>Identifying information: a stamp from the Novosibirsk Customs Office on the customs declaration, saying "Approved For Release" and showing a release date but not a particular document number.<br><br>For samples collected in August 2016<br>Name of issuing authority: the Novosibirsk Customs Office<br>Date of issue: 18/04/2017<br>Identifying information: a stamp from the Novosibirsk Customs Office on the customs declaration, saying "Approved For Release" and showing a release date but not a particular document number.<br><br>For samples collected in August 2017<br>Name of issuing authority: the Novosibirsk Customs Office<br>Date of issue: 16/04/2018<br>Identifying information: a stamp from the Novosibirsk Customs Office on the customs declaration, saying "Approved For Release" and showing a release date but not a particular document number.<br><br>For samples collected in August 2018 and August 2019<br>Name of issuing authority: the Novosibirsk Customs Office<br>Date of issue: 27/07/2021<br>Identifying information: a stamp from the Novosibirsk Customs Office on the customs declaration, saying "Approved For Release" and showing a release date but not a particular document number.<br><br>All samples are imported into Australia under an appropriate permit for quarantined materials issued to Richard G. Roberts (University of Wollongong, New South Wales, Australia):<br><br>For samples collected in August 2012<br>Name of issuing authority: Australian Government, Department of Agriculture, Fisheries and Forestry<br>Date of issue: 01/03/2012 valid until 01/03/2014<br>Identifying information: permit number IP12003422<br><br>For samples collected in August 2016 and August 2017<br>Name of issuing authority: Australian Government, Department of Agriculture and Water Resources<br>Date of issue: 21/06/2016 valid until 21/06/2018<br>Identifying information: permit number 0000480685<br><br>For samples collected in August 2018 and August 2019<br>Name of issuing authority: Australian Government, Department of Agriculture, Water and the Environment<br>Date of issue: 10/07/2020 valid until 10/07/2022

	Identifying information: permit number 0004378957<br><br>An agreement of scientific cooperation between the Institute of Archaeology and Ethnography, Siberian Branch of the Russian Academy of Sciences and the University of Wollongong for projects in the field of geochronology was first signed on 28 March 2012, with the most recent variation signed on 29 January 2019. An agreement of scientific cooperation between the Institute of Archaeology and Ethnography, Siberian Branch of the Russian Academy of Sciences and the Max Planck Institute for Evolutionary Anthropology for projects in the field of palaeogenetics in North Asia was signed on 25 December 2018, with the most recent variation signed on 18 September 2023.
Disturbance	This site has been excavated continuously by archaeologists. We took OSL samples, micromorphology samples and sediment DNA samples from exposed profiles under the supervision of archaeologists so that disturbance was minimised. The site was closed and protected at the end of each excavation season and there is a full-time caretaker that lives near the site.

Reporting for specific materials, systems and methods

We require information from authors about some types of materials, experimental systems and methods used in many studies. Here, indicate whether each material, system or method listed is relevant to your study. If you are not sure if a list item applies to your research, read the appropriate section before selecting a response.

Materials & experimental systems

n/a	Involved in the study
☒	☐ Antibodies
☒	☐ Eukaryotic cell lines
☐	☒ Palaeontology and archaeology
☒	☐ Animals and other organisms
☒	☐ Clinical data
☒	☐ Dual use research of concern
☒	☐ Plants

Methods

n/a	Involved in the study
☒	☐ ChIP-seq
☒	☐ Flow cytometry
☒	☐ MRI-based neuroimaging

Palaeontology and Archaeology

Specimen provenance	An agreement of scientific cooperation between the Institute of Archaeology and Ethnography, Siberian Branch of the Russian Academy of Sciences and the University of Wollongong for projects in the field of geochronology was first signed on 28 March 2012, with the most recent variation signed on 29 January 2019. An agreement of scientific cooperation between the Institute of Archaeology and Ethnography, Siberian Branch of the Russian Academy of Sciences and the Max Planck Institute for Evolutionary Anthropology for projects in the field of palaeogenetics in North Asia was signed on 25 December 2018, with the most recent variation signed on 18 September 2023.
Specimen deposition	All sediment samples remain stored at the Max Planck Institute for Evolutionary Anthropology in Leipzig, Germany and at the University of Wollongong in Australia.
Dating methods	See section above - Ecological, evolutionary & environmental sciences study design. The manuscript explains in detail the dating methods and results.

☒ Tick this box to confirm that the raw and calibrated dates are available in the paper or in Supplementary Information.

Ethics oversight	All necessary permits for excavations at Denisova Cave were obtained by the Institute of Archaeology and Ethnography, Siberian Branch of the Russian Academy of Science from the Ministry of Culture of the Russian Federation.

Note that full information on the approval of the study protocol must also be provided in the manuscript.

Plants

Seed stocks	Not applicable
Novel plant genotypes	Not applicable
Authentication	Not applicable
